# Benchmarking Vision Encoders for Image Classification in Ophthalmology

**DOI:** 10.34133/csbj.0178

**Published:** 2026-09-01

**Authors:** Jay Zoellin, Colin Merk, Bence György

**Affiliations:** ^1^ Institute of Molecular and Clinical Ophthalmology Basel (IOB), Basel, Switzerland.; ^2^ Department of Ophthalmology, University of Basel, Basel, Switzerland.

## Abstract

Foundation vision encoders are rapidly emerging as the standard for retinal artificial intelligence. Yet, ophthalmology still lacks a comprehensive benchmark, leaving model selection for basic science and clinical translation as guesswork. Here, we present a large-scale comparison of 34 pretrained encoders on 39 classification tasks covering color fundus photography, optical coherence tomography, scanning laser ophthalmoscopy, and ultrawidefield imaging. Using a unified pipeline, we compare frozen-feature evaluation, linear probing, and end-to-end fine-tuning to determine which models translate into strong downstream performance. We show that ophthalmic transfer is highly task dependent: no single encoder dominates, and model rankings vary across datasets. Contrary to common expectations, retina-specific pretraining does not confer an advantage. Instead, several natural-image and cross-domain medical encoders match or surpass ophthalmology-specialized models, with the histopathology-pretrained Virchow achieving the strongest overall performance. In addition, pathology-pretrained encoders consistently place near the top, revealing the value of cross-domain pretraining for ophthalmic applications. We further show that inexpensive proxy evaluations are unreliable substitutes for full fine-tuning. Across fairness analyses, all encoders exhibit similar age- and sex-associated performance gaps, and larger models appear more sensitive to suboptimal learning rates, whereas smaller encoders are robust. Together, these findings provide an objective reference for encoder selection in ophthalmology and show that reliable retinal artificial intelligence depends not only on model scale or domain-specific pretraining but also on careful, protocol-aware evaluation. By releasing our code, splits, and benchmarking pipeline, we aim to establish a transparent foundation for future ophthalmic foundation-model research.

## Introduction

### Motivation

Ophthalmology is a medical specialty in which imaging is fundamental to both clinical care and research [1,2]. Clinicians rely on a variety of imaging modalities, including color fundus photography (CFP), optical coherence tomography (OCT), ultrawidefield imaging (UWF), and scanning laser ophthalmoscopy (SLO), to detect, stage, and monitor various retinal diseases [1–6]. Early and accurate diagnosis enables timely interventions, improves patient outcomes, and supports efficient recruitment for clinical trials [7–10]. Given the high volume of imaging data produced in ophthalmology, automated image analysis has advanced rapidly and is progressing toward routine ophthalmic care [11–13]. Several autonomous artificial intelligence (AI)-based diagnostic systems have already received regulatory approval, underscoring the growing clinical relevance of computer vision in ophthalmology [13–15].

Deep learning is poised to change the field of medical image analysis, with convolutional network models previously representing the state of the art for medical image classification tasks. Recently, the introduction of vision transformer (ViT) architectures has marked a pivotal shift in the field [11,16–19]. In particular, foundation models, vision encoders pretrained on massive and diverse datasets, have redefined the paradigm of transfer learning by enabling generalization across both tasks and modalities [11,20]. While some of these models are tailored specifically for medical imaging or ophthalmology, others originate from unrelated domains such as natural images and require domain-adaptation strategies [17,21–23].

Since the introduction of the first retina-specific foundation model, RETFound [11], numerous ophthalmology-oriented encoders have emerged, including RET-CLIP, BE-DINORET, and other specialized architectures [23–25]. In parallel, an unprecedented number of natural-domain foundation models are being released, many showing strong potential for ophthalmic applications [21,23,26]. However, comparative evaluations remain fragmented, with most studies focusing on limited disease diversities, single imaging modalities, or small-scale datasets. Benchmarking is further complicated by methodological inconsistencies, including differing fine-tuning strategies, hyperparameter settings, and performance metrics. Thus, distinguishing true architectural improvements from experimental variance remains challenging [23,27]. Consequently, despite the proliferation of retina-specific foundation models, the field lacks a systematic, head-to-head benchmark across a wide range of ophthalmic tasks and modalities.

From both clinical and methodological perspectives, it is essential to understand how vision encoders pretrained on diverse domains and with different architectures perform when applied to ophthalmology [11,28]. Key open questions include the following: Which foundation models generalize best to ophthalmic tasks? Are certain architectures or pretraining domains better suited for specific imaging modalities? Do encoders trained on other medical domains, such as histopathology, transfer effectively to retinal image analysis? And to what extent do pretraining data and methodology shape downstream performance in ophthalmology?

To address these questions, we present the first large-scale, systematic, and reproducible benchmark of foundation vision models in ophthalmology. We focus on image classification tasks due to their simplicity, reproducibility, and abundance of publicly available datasets, enabling consistent evaluations across a wide range of encoders. By unifying evaluation pipelines and adaptation strategies, our framework establishes a practical and standardized benchmark in ophthalmology and sets the stage for more rigorous and transparent comparisons in the field.

### Related works

Standardized benchmarks have been instrumental in advancing the field of computer vision. Large-scale datasets such as ImageNet [29] have fueled the development of novel architectures and training paradigms by providing a common ground for evaluating model performance [30]. This success has inspired the creation of benchmarks in specialized domains, including medical imaging [31–33]. In fields like radiology, pathology, or general medical vision tasks, comprehensive benchmarks enable rigorous comparisons of pretrained foundation models [31,32,34–36]. These efforts not only have driven progress but also highlight the importance of domain-specific pretraining and adaptation strategies [31–33]. Ophthalmology, however, despite being a leading field in computer vision, lacks a similar large-scale, multimodal, and cross-disease benchmark [37].

The advent of foundation models, particularly ViT models pretrained on extensive datasets, has shifted the paradigm from training task-specific models from scratch to fine-tuning general-purpose encoders [21,38]. In medical imaging, this approach has been successfully applied to various modalities [11,22]. Models originally trained on natural-domain images have demonstrated remarkable transferability to medical tasks with appropriate fine-tuning [23,37,39]. Concurrently, domain-specific foundation models, pretrained on large medical imaging archives, such as chest x-rays or histology slides, have shown competitive or superior performance, suggesting a benefit from in-domain pretraining data [11,22,40]. This dichotomy between general-domain and medical-specific pretraining raises critical questions about which approach is most effective for highly specialized fields, such as ophthalmology [26,37,39].

DL has achieved substantial success in ophthalmic image analysis, with models demonstrating high-level performance for diagnostics [11,12,19,41]. Many early systems relied on convolutional network models trained for specific tasks [42,43]. More recently, the field has moved toward leveraging foundation models [19,23,37]. The first ophthalmology-specific foundation model, RETFound, demonstrated the potential of pretraining on a large number of retinal images [11]. Since its introduction, several other retina-specific models have been developed, reporting incremental improvements in various downstream tasks [19,23,24,44]. In parallel, studies have shown that natural-domain encoders can achieve strong performance on ophthalmic tasks, often rivaling intradomain pretraining [26,37,39].

Despite this rapid progress, existing studies typically compare a new model against a small set of baselines, with comparisons often limited to a single disease or imaging modality. Performance evaluations are frequently conducted using a limited number of evaluation datasets and inconsistent fine-tuning protocols, making direct and fair comparisons between models difficult. This lack of a standardized evaluation framework creates ambiguity regarding the true state of the art and obscures the factors that drive performance, such as model architecture, pretraining domain, and adaptation strategy. Our work directly addresses this gap by establishing the first large-scale, systematic benchmark to rigorously evaluate and compare a wide array of vision encoders for ophthalmic image classification.

### Contributions

In this work, we present the first large-scale benchmark of vision encoders for ophthalmic image classification. We evaluate 34 vision encoders, spanning medical, natural-domain, histology, radiology, and ophthalmology-specific models, on 39 classification tasks from publicly available datasets covering a wide range of diseases and imaging modalities. Our contributions are 3-fold: (a) We provide a standardized, reproducible evaluation pipeline using publicly available datasets and consistent metrics; (b) we comprehensively analyze encoder performance across domains, modalities, and diseases, offering practical guidance for both research and clinical model selection; and (c) we release our benchmarking code as open-source, facilitating transparent comparison and further research in this area.

## Methods

This section describes the experimental framework, including the vision encoders, evaluation datasets, a unified training protocol, and performance metrics.

### Vision encoders

The benchmark includes 34 vision encoders representing diverse pretraining domains and architectures. The models are grouped into the following categories:

• Natural-domain: Models pretrained on natural images, including DINOv2 [21], SigLIP [45], and CLIP [38].
• Medical cross-specialty: Models from other medical fields to assess transfer learning, including chest-x-ray-pretrained encoders such as RadDINO [46] and histopathology-pretrained encoders such as Virchow [22].
• Ophthalmology-specific: Models trained on retinal images, including models trained on CFP only, such as RetiZero [25] and DINORET [23]; models pretrained on OCT only; and models pretrained on combined CFP and OCT data, for example, UrFound [44].
• Non-ViT baselines: A randomly initialized and an ImageNet-pretrained ResNet-50 [47] are included as classical nontransformer benchmarks.

In addition to these representative models, the benchmark includes DINOv2 variants with register tokens [48], Prov-GigaPath [49], RET-CLIP [24], RETFound DINOv2 variants [50], RETFound MAE variants [11], RETFound Green [51], SigLIP2 [52], ViLReF [53], Virchow2 [40], VisionFM Fundus and VisionFM OCT [19], and an ImageNet-pretrained ViT-B [16]. Table A.1 in Appendix A.2 provides a complete list of all encoders, their pretraining data, and training methods.

### Datasets

The encoders were evaluated on 21 public datasets with 39 classification tasks including CFP, OCT, SLO, and UWF images. The tasks are categorized into multidisease classification; disease-specific classifications, such as diabetic retinopathy (DR), glaucoma, or other retinal pathologies; systemic health predictions; and visual function predictions. We created new stratified data splits of 70% training, 15% validation, and 15% test data. Splits were stratified by participant identity (wherever available), preventing data leakage. All images were resized to 224 × 224 pixels to standardize input. The datasets used are Harvard FairVision [54], RFMiD [55], Open UWF [56], OCTID [57], HRF [58], OCT-XRAY [59], APTOS 2019 [60], DRTiD [61], EyePACS [62], IDRiD [63], BRSET [64], mBRSET [65], Messidor [66], PAPILA [67], Harvard FairGenMed [68], Harvard FairFedMed [34], Harvard Glaucoma Fairness [69], JustRAIGS [70], FIMD [71], GRAPE [72], and AI-READI [73]. DR severity was defined according to the International Clinical Diabetic Retinopathy (ICDR) scale [74], with Messidor ICDR labels derived from the grading framework described by Krause *et al.* [75]. Detailed task definitions, sample selection criteria, class definitions, preprocessing, data leakage assessments, dataset access information, and setup instructions are provided in Appendix A.3 and in the accompanying code repository.

Several datasets include demographic information for fairness analysis. We evaluate model performance across sex, grouped as male and female, and age, binarized into under 60 and 60 and over. Table 1 summarizes the distribution of these attributes for datasets where such information is available.

**Table 1.** Distribution of sensitive attributes in datasets used for fairness analysis. Age groups are binarized as “under 60” and “60 and over”.

Dataset	Attribute	Group 1 (%)	Group 2 (%)
JustRAIGS	Age	59.3 (under 60)	40.7 (60+)
AI-READI	Age	47.0 (under 60)	53.0 (60+)
BRSET	Sex	61.8 (female)	38.2 (male)
mBRSET	Sex	65.0 (female)	35.0 (male)
Harvard FairGenMed	Sex	58.2 (female)	41.8 (male)
Harvard FairFedMed	Sex	57.0 (female)	43.0 (male)
Harvard Glaucoma Fairness	Sex	54.9 (female)	45.1 (male)
Harvard FairVision (all)	Sex	57.1 (female)	42.9 (male)

#### Preprocessing and augmentations

A consistent data augmentation strategy was used during training. Augmentations included random resized cropping with a scale of 0.8 to 1.2, random rotation of ±5°, random horizontal and vertical flipping, and color jittering with factors of 0.2 for brightness and contrast and 0.1 for saturation and hue. All images were normalized using ImageNet statistics unless an encoder’s official pipeline specified otherwise. Grayscale images were converted to 3 channels by replicating the single channel into 3.

### Training protocol

All encoders were trained following a unified protocol. For each transformer encoder, we appended a single linear classification head to the model backbone, receiving the classification (CLS) token embedding as input. For nontransformer models—specifically, the ResNet-50 variants—we used standard output embeddings. Each model was evaluated under 2 distinct training regimes: linear probing, in which the encoder weights were frozen, and full fine-tuning, in which all model weights were updated end to end to adapt the feature representations [27]. All experiments were repeated 3 times with independent random seeds to ensure robustness. For fine-tuning, we used the AdamW optimizer [76] with a learning rate scheduler combining a linear warm-up for the first 10% of steps and a subsequent cosine annealing decay. Training length was standardized to 25 steps per epoch to accommodate different dataset sizes; thus, epoch denotes a bookkeeping unit rather than a full dataset pass. All models were trained for 100 epochs with a batch size of 64. The final model weights were selected based on the lowest validation loss.

#### Class imbalance handling

Logarithmic class weighting was applied during training to mitigate class imbalance. The weight $w_{i}$ for a class *i* with $c_{i}$ samples was calculated as $w_{i}=1\;/\;\log\left(c_{i}+1\right)$ and subsequently normalized. This method up-weights rare classes moderately, which prevents model instability from extreme weights.

#### Hyperparameter tuning

A systematic grid search was performed to identify encoder-specific optimal learning rates for the encoder backbones (${\mathrm{lr}}_{\text{encoder}}$) and the classification heads (${\mathrm{lr}}_{\text{classifier}}$). The search was performed once per encoder for the classification of DR from the CFP images of the AI-READI dataset [73], and the resulting optimal pair was used for all other datasets. The procedure consisted of 2 stages. First, we identified the optimal classifier learning rate, ${\mathrm{lr}}_{\text{classifier}}\in \left\{10^{-4},\;10^{-3},\;10^{-2}\right\}$, using linear probing. In this stage, the encoder was frozen and only the classification head was trained, with one run performed for each candidate classifier learning rate. Second, during full fine-tuning, we kept the selected ${\mathrm{lr}}_{\text{classifier}}$ fixed for the classification head and searched over encoder learning rates, ${\mathrm{lr}}_{\text{encoder}}\in \left\{10^{-7},\;10^{-6},\;10^{-5},\;10^{-4},\;10^{-3}\right\}$. During this second stage, both the encoder and classification head weights were updated; however, only ${\mathrm{lr}}_{\text{encoder}}$ was varied across runs, whereas the classifier learning rate was fixed at the value identified in the first stage. For our sensitivity analysis, we repeated the 2 learning rate sweeps on the EyePACS dataset.

#### Controlling for data leakage

Several encoders included in our benchmark had documented exposure to one or more datasets that were also used for downstream evaluation. To assess whether such overlap influenced our conclusions, we first identified all evaluation datasets that were known to be incorporated into the pretraining corpus of at least one encoder and repeated the analysis on a restricted benchmark excluding these tasks.

In addition, some openly accessible ophthalmic datasets may have been included in large-scale web-derived pretraining corpora (i.e., large internet-derived image datasets used for model pretraining) used by general-purpose vision encoders, even when such overlap is not explicitly documented. We therefore performed a second sensitivity analysis restricted to datasets with a low risk of inadvertent pretraining exposure. Datasets were considered low risk when access required a human-mediated request, approval, or manually granted permission, rather than unrestricted public download. The estimated risk of pretraining data leakage and the presence of definitive data leakage for each classification task are reported in the dataset overview in Appendix A.3.

### Evaluation metrics and analysis

#### Core protocols and metrics

Performance was measured under 3 evaluation protocols: (a) *k*-nearest neighbor (kNN) classification on frozen features, (b) linear probing accuracy, and (c) full fine-tuning accuracy [27]. Reported accuracies for linear probing and full fine-tuning are the mean and standard deviation across 3 seeded runs. The models were ranked on each task, and the mean rank serves as the primary aggregate metric for cross-encoder comparison.

#### Robustness to class imbalance

Robustness was analyzed using 2 parallel training regimes. In the first, models were trained on the original, unbalanced datasets with class weighting and evaluated on the original test set (u-u) and a class-balanced test set created via undersampling the entire dataset once (u-b). In the second approach, models were trained on balanced training sets, created via undersampling, without class weighting and evaluated on a balanced test set (b-b) and the original test set (b-u).

#### In-depth and fairness analysis

Results were stratified by imaging modality, either CFP or OCT, and by clinical task, such as DR or glaucoma classification. Model stability was assessed by measuring sensitivity to learning rates. Fairness was analyzed by measuring performance gaps between demographic subgroups for sex and age.

#### Statistical analysis

All statistical analyses were performed on encoder ranks rather than raw accuracies to enable comparisons across different datasets. For each task, test accuracies were averaged over 3 runs with different random seeds and converted into ranks, where lower ranks indicate better performance. To assess overall differences between encoders across multiple tasks, we applied the Friedman test, followed by the Nemenyi *post hoc* test for pairwise comparisons. Results are visualized using critical difference plots. Agreement between evaluation protocols (full fine-tuning, linear probing, and kNN) and between balanced and unbalanced dataset configurations was quantified using Spearman’s rank correlation coefficient (ρ). To test for differences in performance trends between encoder groups (e.g., grouped by pretraining domain), we used the Kruskal–Wallis test on derived rank-distance measures. All tests were 2-sided, and the statistical significance threshold was set at an alpha of 0.05.

### Explainability

For representative datasets, we selected one test image per class and generated attribution maps from the trained model checkpoints using integrated gradients with Captum’s NoiseTunnel wrapper [77]. Attributions were computed with respect to the model’s predicted class on resized 224 × 224 inputs, using 50 noisy samples and Gaussian noise with a standard deviation of 0.3. The resulting attribution maps were converted to grayscale, contrast-enhanced with CLAHE [78], and rendered as heatmaps. The resulting images are shown in Figs. A.7 to A.9.

## Experiments

### Full fine-tuning

**Fig. 1.**
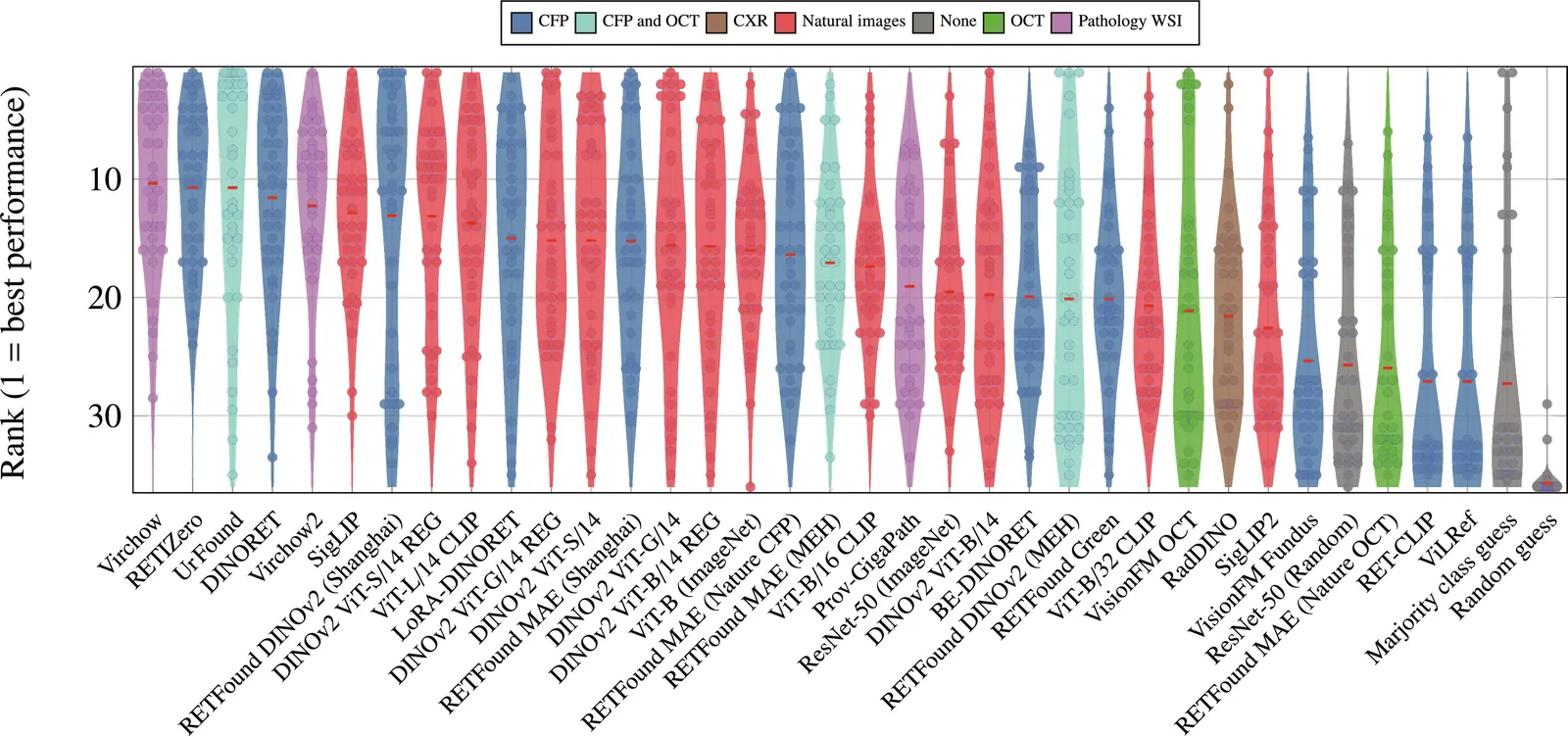
Rank distribution of 34 vision encoders across 39 ophthalmic image classification tasks after full fine-tuning. Lower ranks indicate better performance. Each point represents the rank of a model for a specific task, based on the average test accuracy over 3 runs with different random seeds. Violin plots summarize the distribution of ranks per encoder, with colors indicating pretraining domain: color fundus photography (CFP), optical coherence tomography (OCT), natural images, pathology whole-slide images, or none. Red horizontal lines mark the mean rank of each encoder across all tasks. Models are listed by descending mean performance across all 39 tasks from left to right.

When adapting foundation models for new clinically relevant tasks, a common approach is to jointly train both the encoder and the classification head, a process we refer to as full fine-tuning. This strategy not only is widely used in practice but also serves as the most common evaluation protocol when comparing newly proposed models [11,28]. Applying full fine-tuning across all 34 encoders in our benchmark yielded several surprising and important findings, as shown in Fig. 1. A statistical comparison across encoders is provided in Fig. A.1 in the form of a critical difference plot.

#### In-domain pretraining does not guarantee superior performance

Contrary to expectations, in-domain pretraining does not systematically translate to superior performance. As shown in Fig. 1, no consistent advantage for encoders pretrained on retinal images was observed. For instance, some of the lowest-ranked models include those pretrained on CFP (RET-CLIP), natural images (SigLIP2), and OCT (RETFound MAE [Nature OCT]). Notably, both encoders pretrained exclusively on OCT data exhibit relatively poor mean ranks overall (25.96 ± 8.62 for RETFound MAE [Nature OCT] and 21.13 ± 11.04 for VisionFM OCT), which may reflect the predominance of RGB datasets in our benchmark (Appendix A.3). Notably, we observed a strong performance of encoders pretrained on histopathology images. The best (lowest) mean rank was achieved by Virchow (10.36 ± 7.15), which was pretrained solely on histopathology slides, without exposure to ophthalmic images prior to fine-tuning. Among the top 5-ranked models, 2 (Virchow and Virchow2) were pathology pretrained. Only 3 out of the top 5 encoders were pretrained on retinal images, namely, RetiZero, UrFound, and DINORET, suggesting that the semantically consistent features needed for retinal classification can be learned from other domains, particularly histopathology slides and natural-domain images.

#### Performance is highly task dependent

Encoder performance varies dramatically across tasks, with no single model consistently outperforming the rest. While Virchow achieves state-of-the-art results on several datasets (mean accuracies of 80.82% on OUWWFI and 59.22% on AI-READI OCT), it also ranks among the lowest-performing encoders on others (e.g., mean accuracy of 65.77% on PAPILA). Nearly all encoders rank as both top and bottom performers for different tasks, highlighted by the wide rank distribution in Fig. 1. For instance, DINOv2 ViT-S Reg is the best-performing encoder on 2 datasets (mean accuracy of 74.31% on Harvard FairVision Glaucoma and 65.84% on IDRiD) but simultaneously ranks among the worst 10 encoders for at least 8 other tasks. Overall, the results reveal extremely wide rank distributions across tasks, with encoders excelling on some but failing on others.

#### Controlling for potential data leakage

**Fig. 2.**
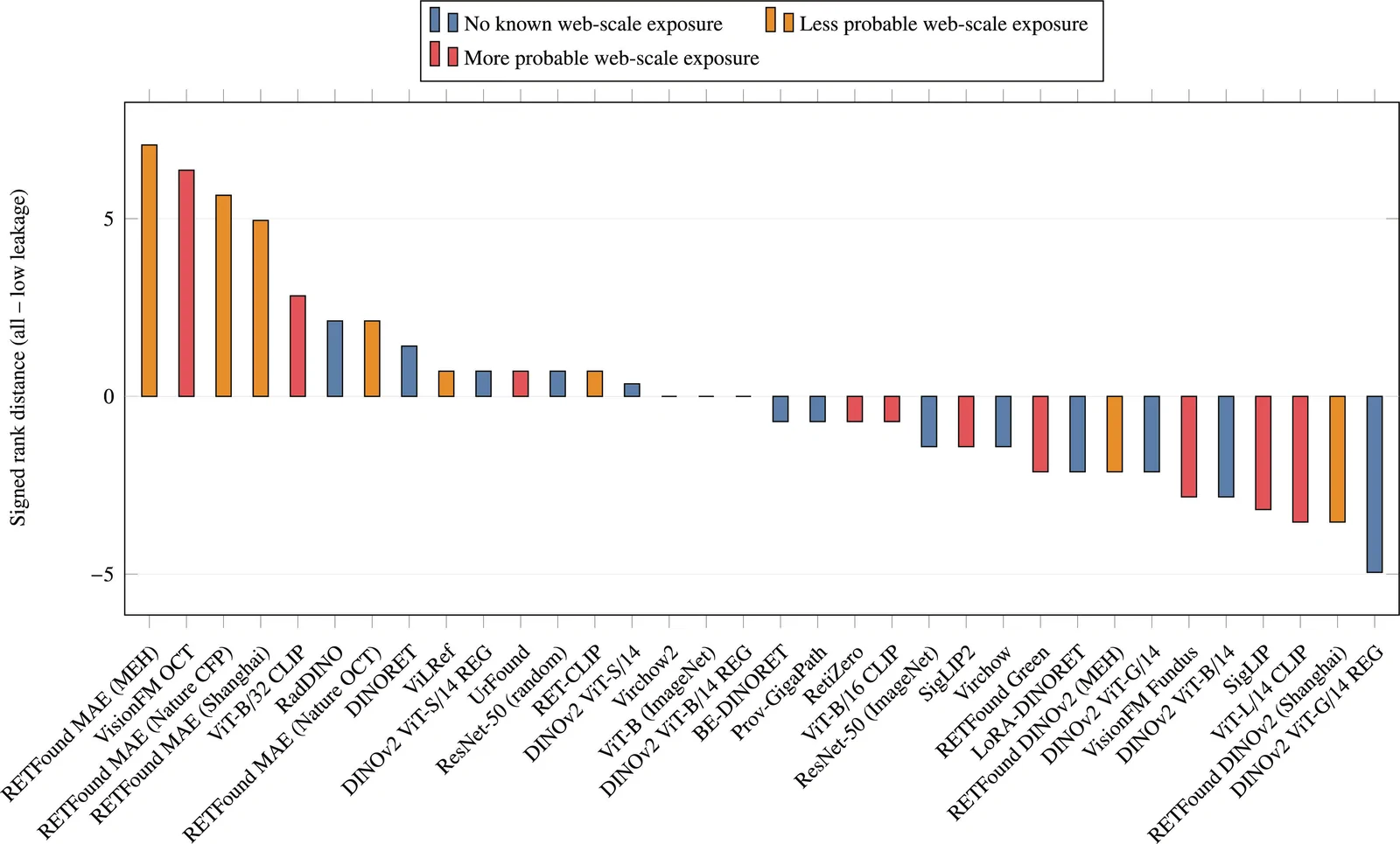
Signed change in encoder ranking when evaluation is restricted to datasets marked as unlikely to have appeared in web-scale training corpora. Positive values indicate improved rank on the low-leakage subset relative to the full mapped benchmark set, while negative values indicate worse ranks. Bar colors denote each encoder’s estimated likelihood of web-scale data exposure.

To account for potential data leakage, where an encoder’s pretraining data might overlap with benchmark datasets, we performed a filtered analysis excluding tasks with known pretraining exposure. As shown in Figs. A.2 and A.4, excluding these datasets did not alter the main findings: RetiZero, the encoder with the highest data leakage risk, remained a top-ranked encoder [25]. Similarly, some evaluation datasets may have appeared in the pretraining data of encoders trained on web-scale corpora. Figure 2 shows how each model’s rank changes when the evaluation is restricted to datasets that are unlikely to be directly scrapeable, for example, because access requires a human request or approval. Surprisingly, encoders with no known web-scale exposure do not systematically exhibit positive signed rank changes, and encoders with potential web-scale exposure do not consistently exhibit negative signed rank changes.

### Feature evaluation with linear probing

Beyond end-to-end fine-tuning, we also assess the representations learned by each encoder using linear probing, where encoder weights are frozen and only a linear classifier is trainable. Contrary to full fine-tuning, which tests how well encoders can be optimized for new tasks, linear probing measures the extent to which discriminative information is already contained in the out-of-the-box feature representations.

#### Full fine-tuning consistently outperforms linear probing

**Fig. 3.**
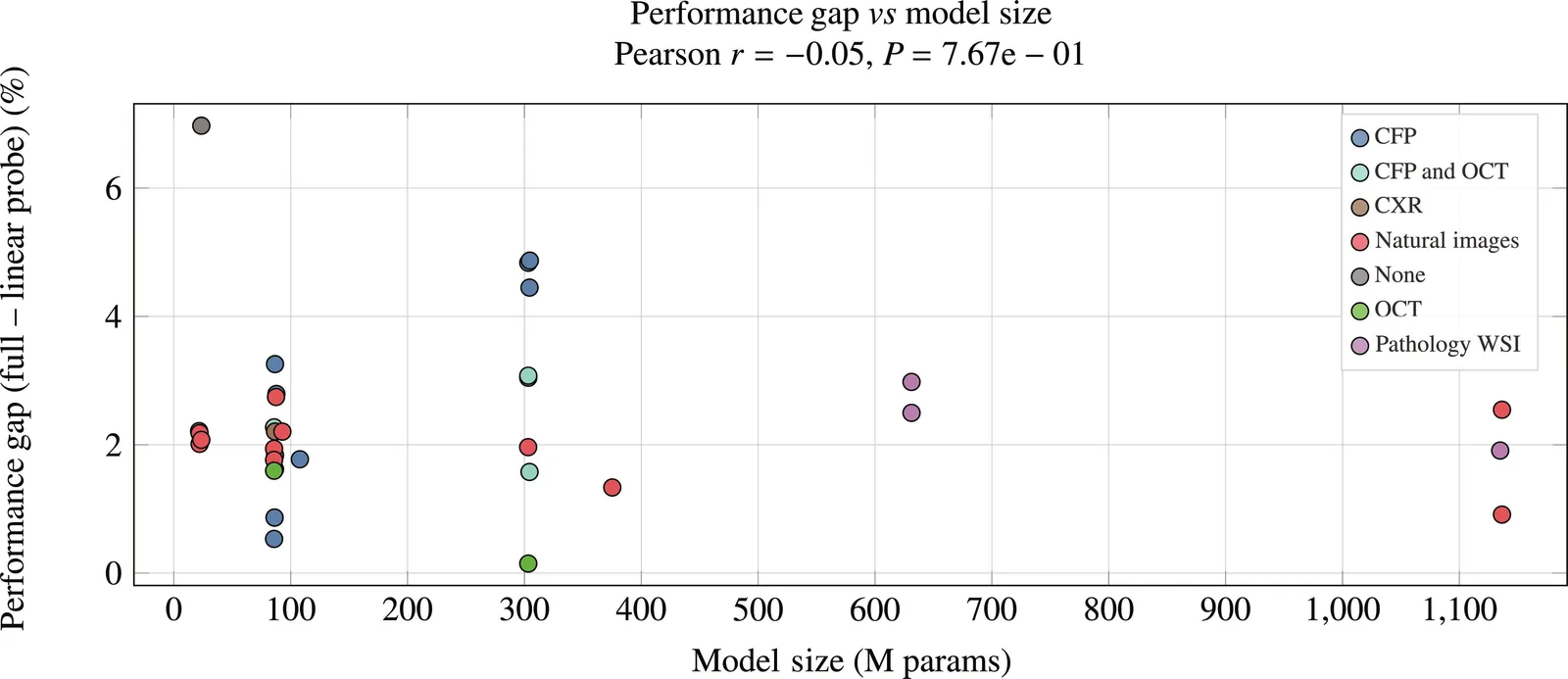
Performance gap between full fine-tuning and linear probing, calculated as the difference in mean accuracy across all datasets. A larger gap indicates a greater benefit from fine-tuning the full network. This plot shows each encoder as one scatter point with the number of parameters indicated by its *x* value and the performance gap denoted by the *y* value. The displayed performance gap represents the mean accuracy gap across all datasets, with 3 replicates per dataset using random seeds.

Comparing full fine-tuning to linear probing provides insight into both the intrinsic separability of features learned during pretraining and the additional improvements achievable through task-specific adaptation. As shown in Fig. 3, all encoders achieve higher mean accuracies with full fine-tuning than with linear probing. Further optimization of encoder weights thus seemingly continues to improve the representation of retinal images beyond what is captured in the base embeddings. As expected, the largest performance gap is observed for a randomly initialized ResNet-50 model, which relies entirely on supervised adaptation to learn discriminative embeddings, as shown in Fig. A.5. Conversely, several models show relatively small accuracy gaps between linear probing and full fine-tuning. For instance, DINOv2 ViT-G/14 exhibits only a modest difference (mean accuracy difference across all datasets of 0.91%), as shown in Appendix A.4 in Fig. A.5. Surprisingly, the gap size is not systematically larger for models pretrained on out-of-domain data compared to those trained on retina-specific images (Fig. 3). Neither model size nor pretraining domain consistently predicts the extent of improvement with full fine-tuning (Fig. 3), and model size does not correlate with performance gains upon unfreezing the backbone.

#### Linear probing reveals a new performance hierarchy

**Fig. 4.**
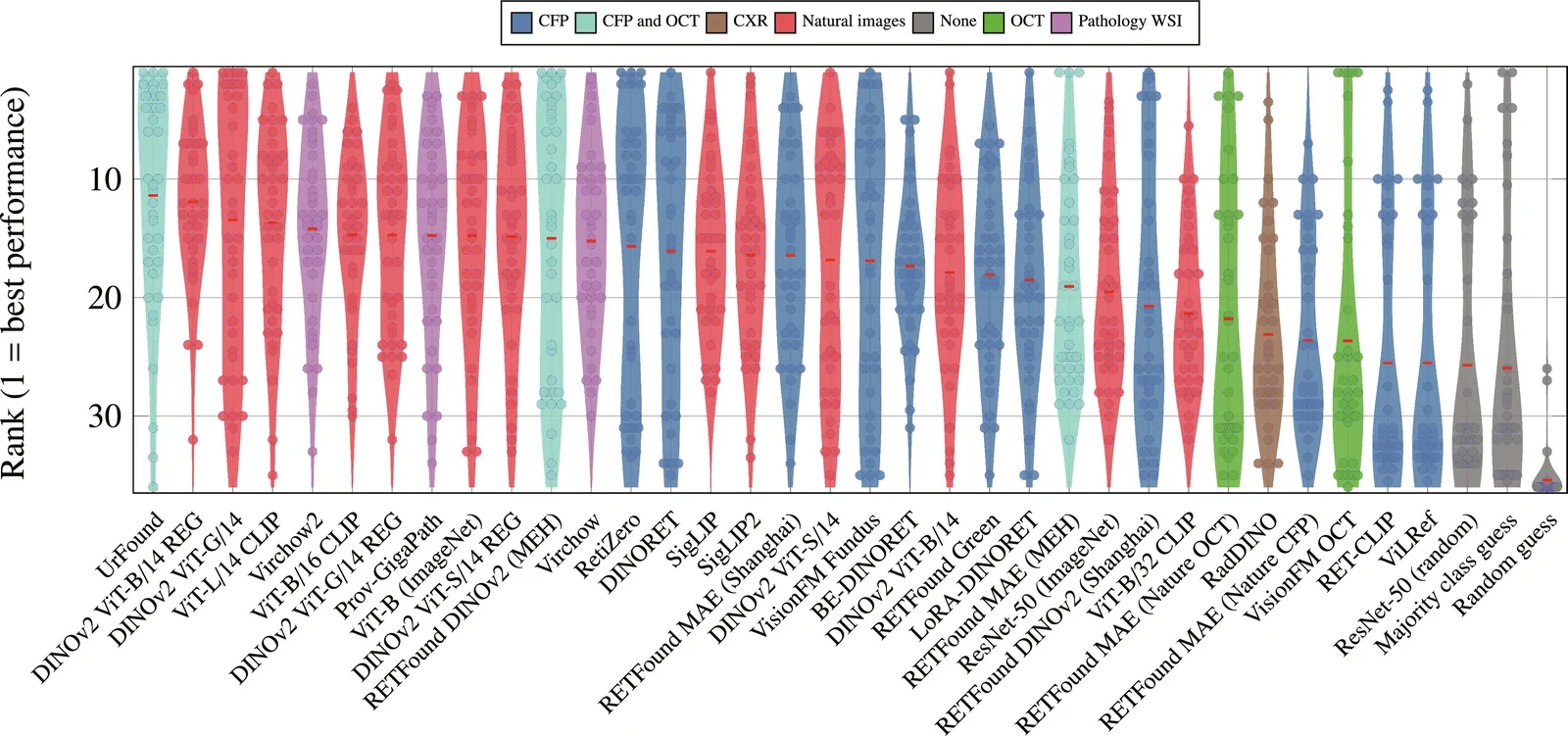
Rank distribution of 34 vision encoders across 39 ophthalmic image classification tasks under the linear probing protocol. Lower ranks indicate better performance. Each point represents the rank of a model on a specific task, based on the average test accuracy over 3 runs with different random seeds. Violin plots summarize the distribution of ranks per encoder, with colors indicating the pretraining domain. Red horizontal lines mark the mean rank of each encoder across all tasks. Models are listed by descending mean performance across all 39 tasks from left to right.

The ranking of models under linear probing, shown in Fig. 4, differs substantially from the results obtained with full fine-tuning. Notably, while Virchow is the top performer in the full fine-tuning setting, it drops to 12th place under linear probing. UrFound, which was pretrained jointly on OCT and CFP data, emerges as the top-performing model under linear probing evaluations, achieving a mean rank of 11.40 ± 9.46. Interestingly, several natural-domain pretrained encoders also appear among the highest performers, such as DINOv2 ViT-B/14 REG (mean rank 11.92 ± 6.53) and ViT-L/14 CLIP (mean rank 13.68 ± 8.35), indicating that general-purpose features learned from natural-domain images can transfer well to retinal tasks. Performance again varies widely across tasks. For one dataset, RFMiD, UrFound, the encoder with the best (lowest) mean rank, ranks as the worst encoder (mean accuracy of 45% on RFMiD). Figure A.3 shows the outcome of hypothesis testing for linear probing evaluations in the form of a critical difference plot.

#### Rankings are unstable across training protocols

**Fig. 5.**
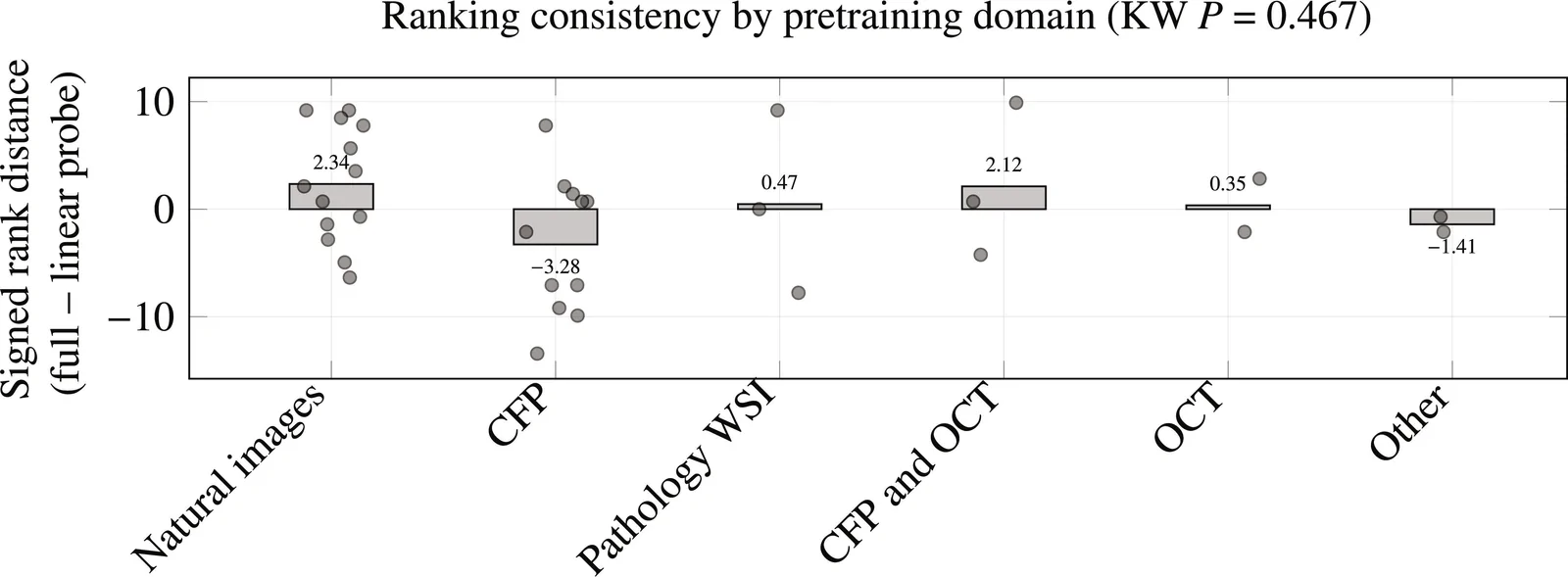
Signed rank distance, showing the change in each encoder’s mean rank between full fine-tuning and linear probing. A positive value indicates a better relative rank under linear probing. The distance is calculated as $\left({\mathrm{rank}}_{\text{Full}}-{\mathrm{rank}}_{\mathrm{LP}}\right)/\sqrt{2}$. This metric geometrically corresponds to the signed perpendicular distance of each model from the y=x diagonal in a scatter plot of the 2 rank types, thus providing a quantitative measure of rank stability. This bar plot shows the mean signed rank distance of all training runs for each pretraining modality. The bars are ordered by the number of encoders contributing to their respective mean. The Other group contains the untrained encoders as well as chest-x-ray-pretrained models. The *P* value derived from the Kruskal–Wallis (KW) test indicates the outcomes of hypothesis testing.

We observed divergent results between full fine-tuning and linear probing outcomes, as shown in Fig. 5. Model rankings shift between full fine-tuning and linear probing. Natural-domain pretrained models generally move up in rank under linear probing, while many CFP-pretrained encoders drop when end-to-end adaptation is not applied, but these differences lack significance. Figure A.6 in Appendix A.4 shows the results for each encoder separately.

### Correlation of evaluation techniques

**Fig. 6.**
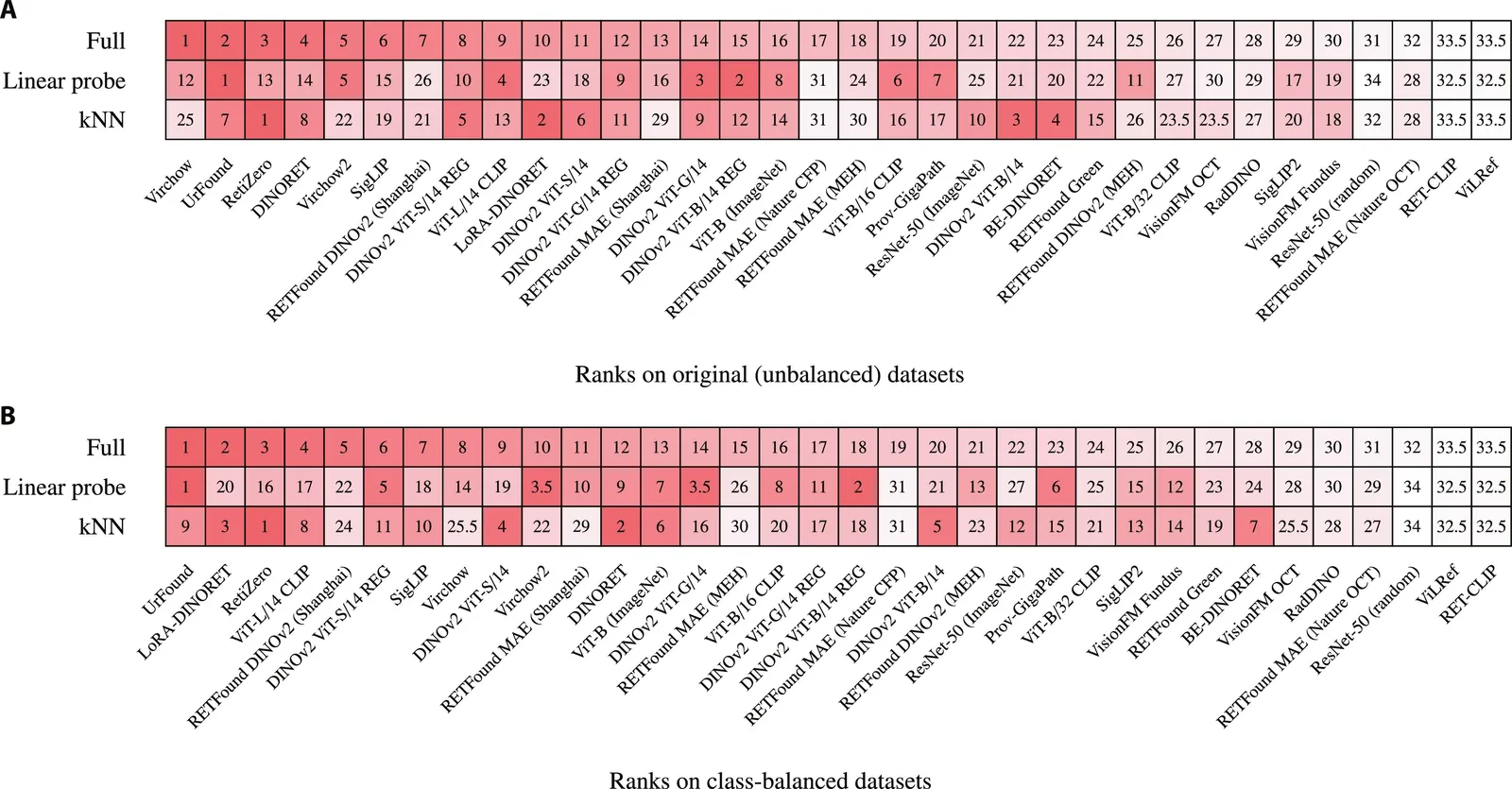
Heatmaps of encoder ranks across 3 evaluation protocols: full fine-tuning (top row), linear probing (middle), and *k*-nearest neighbor (kNN) accuracy (bottom). Brighter colors indicate a better (lower) rank. (A) Ranks on the original (unbalanced) datasets. (B) Ranks on the class-balanced datasets. These visualizations support the quantitative correlation analysis.

**Fig. 7.**
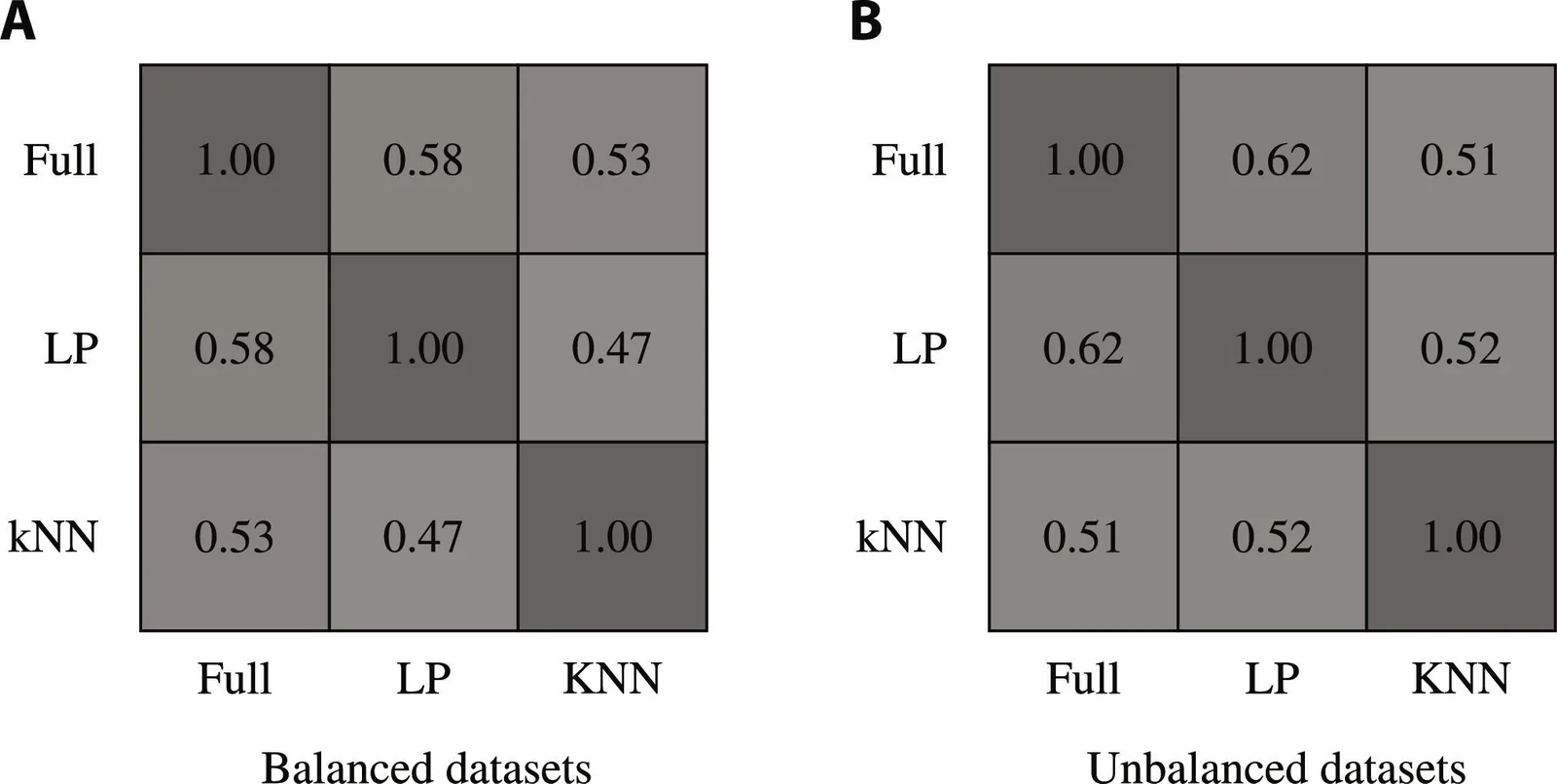
Spearman rank correlation matrix between training and evaluation protocols. Each axis represents model rankings obtained under different protocols, namely, linear probing (LP), full fine-tuning (Full), and *k*-nearest neighbor (kNN). Correlation values indicate the consistency of encoder model rankings across evaluation protocols. (A) shows the correlation values for the balanced datasets, and (B) for the unbalanced datasets.

We next quantified the agreement between different evaluation protocols. A strong correlation implies that computationally inexpensive methods, such as kNN or linear probing, could serve as reliable proxies for full fine-tuning. We compute Spearman’s rank correlations (ρ) between protocols on both the original and class-balanced datasets. The panels in Fig. 6 show the overall mean rankings for each encoder as a heatmap for balanced and unbalanced datasets. Figure 7 shows the corresponding Spearman’s rank correlations.

#### All evaluation methods are moderately correlated

The correlation analysis in Fig. 7 reveals a moderate positive correlation between evaluation protocols. The strongest correlation is consistently found between full fine-tuning and linear probing (ρ≈0.58to0.62). This indicates that models with better out-of-the-box linear separability also tend to achieve higher performance after end-to-end training. The correlations between full fine-tuning and kNN (ρ≈0.51to0.53) and between linear probing and kNN (ρ≈0.47to0.52) are weaker, suggesting that they capture related but distinct properties of the learned representations.

#### Individual model performance highlights the imperfect correlation

The heatmaps in Fig. 6 illustrate the weak correlations between different evaluation strategies. For example, RetiZero is among the top models in full fine-tuning and kNN but ranks poorly in linear probing evaluations. Conversely, models like UrFound rank highly in all evaluation methods.

#### Data balancing has minimal impact on the correlation structure

**Fig. 8.**
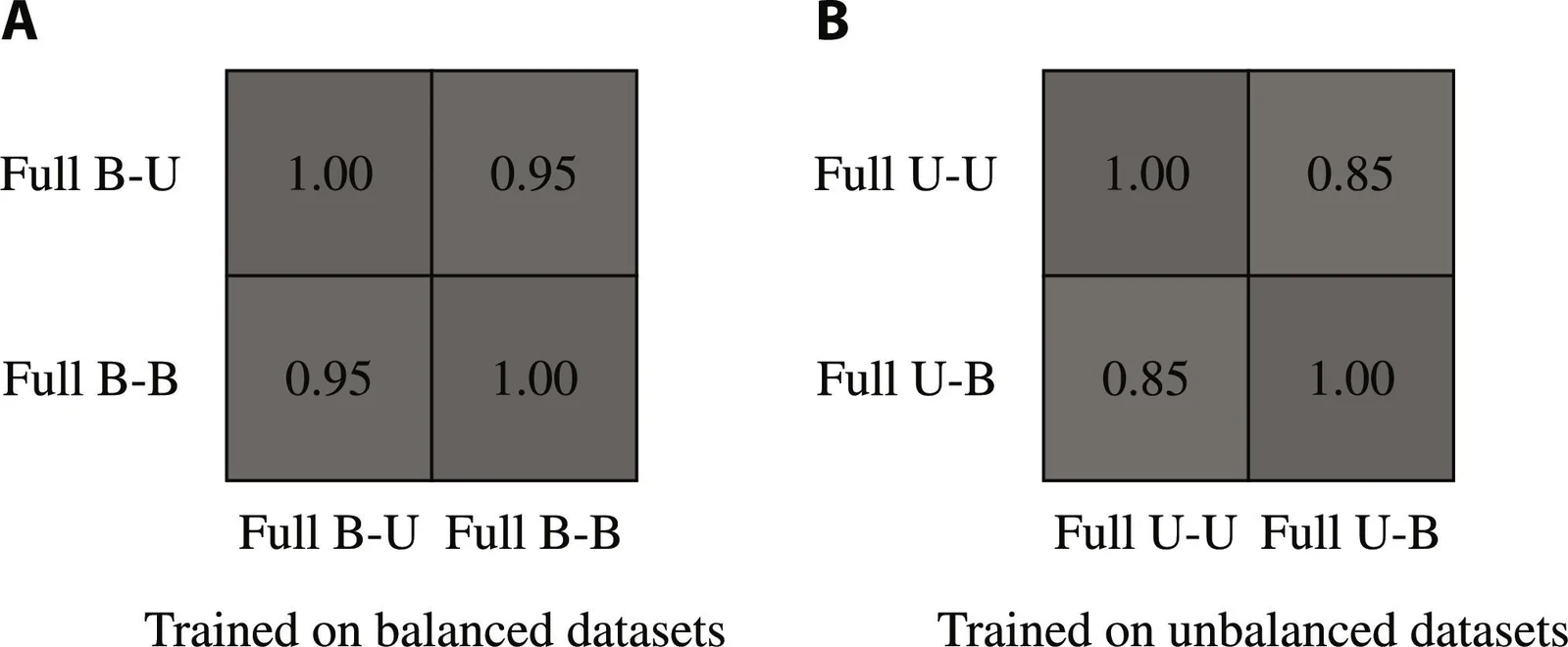
Spearman rank correlation matrices of encoder model rankings between balanced and unbalanced training and test sets. (A) shows results where models were trained on class-balanced datasets, and (B) when models were trained on unbalanced training datasets. Axes correspond to balanced and unbalanced test sets.

Beyond assessing rank consistency across training and evaluation protocols (Fig. 7), we investigated the impact of balancing the training and test sets. Figure 8 shows the Spearman rank correlations across all balanced and unbalanced dataset permutations, with Fig. 8A corresponding to balanced training sets and Fig. 8B to unbalanced training sets. The lowest observed correlation of 0.85 indicates a high degree of rank stability across all configurations. Notably, balanced training leads to slightly more stable rankings when evaluated on unbalanced test sets (Spearman’s ρ=0.95), suggesting that dataset balancing has only a minor influence on the relative performance ordering of models.

### Qualitative interpretability analysis

Because model rankings differed between full fine-tuning and linear probing and because these protocols were only moderately correlated, we performed an additional qualitative interpretability analysis to assess whether selected models relied on visually plausible image regions. We generated NoiseTunnel saliency maps for a general-purpose natural-image pretrained encoder, DINOv2 ViT-B; an ophthalmology-pretrained encoder, RETFound; and a strong pathology-pretrained encoder, Virchow, as shown in Figs. A.7 to A.9. For DR severity grading on APTOS, saliency maps for DINOv2 ViT-B and Virchow frequently highlighted retinal structures within the fundus field, including regions corresponding to retinal vasculature and visible disease-associated lesions such as exudates, hemorrhages, and proliferative changes (Fig. A.7). DINOv2 ViT-B showed relatively similar saliency patterns between full fine-tuning and linear probing, whereas Virchow showed more variable emphasis across adaptation strategies. In contrast, RETFound produced more localized saliency patterns that were less consistently attributable to clearly identifiable anatomical or pathological structures, particularly under linear probing. For OCT disease classification on OCTID, saliency patterns generally aligned with retinal anatomical structures, most clearly for DINOv2 ViT-B and Virchow (Fig. A.8). Similarly, for glaucoma classification on PAPILA, DINOv2 ViT-B and Virchow frequently emphasized the optic nerve head and peripapillary/vascular regions, while RETFound was again less consistently anatomically interpretable (Fig. A.9). The visible checkerboard pattern reflects the underlying ViT patch structure; therefore, these maps were interpreted only at the level of coarse regional patterns rather than individual grid-level activations.

### Domain- and task-specific performance

To determine if model performance is specialized, we analyze how encoder rankings change across subsets of tasks grouped by image modality or disease type. We analyze this for the 2 most abundant modalities, CFP and OCT, and for 2 common tasks, DR staging [74] and glaucoma detection.

**Fig. 9.**
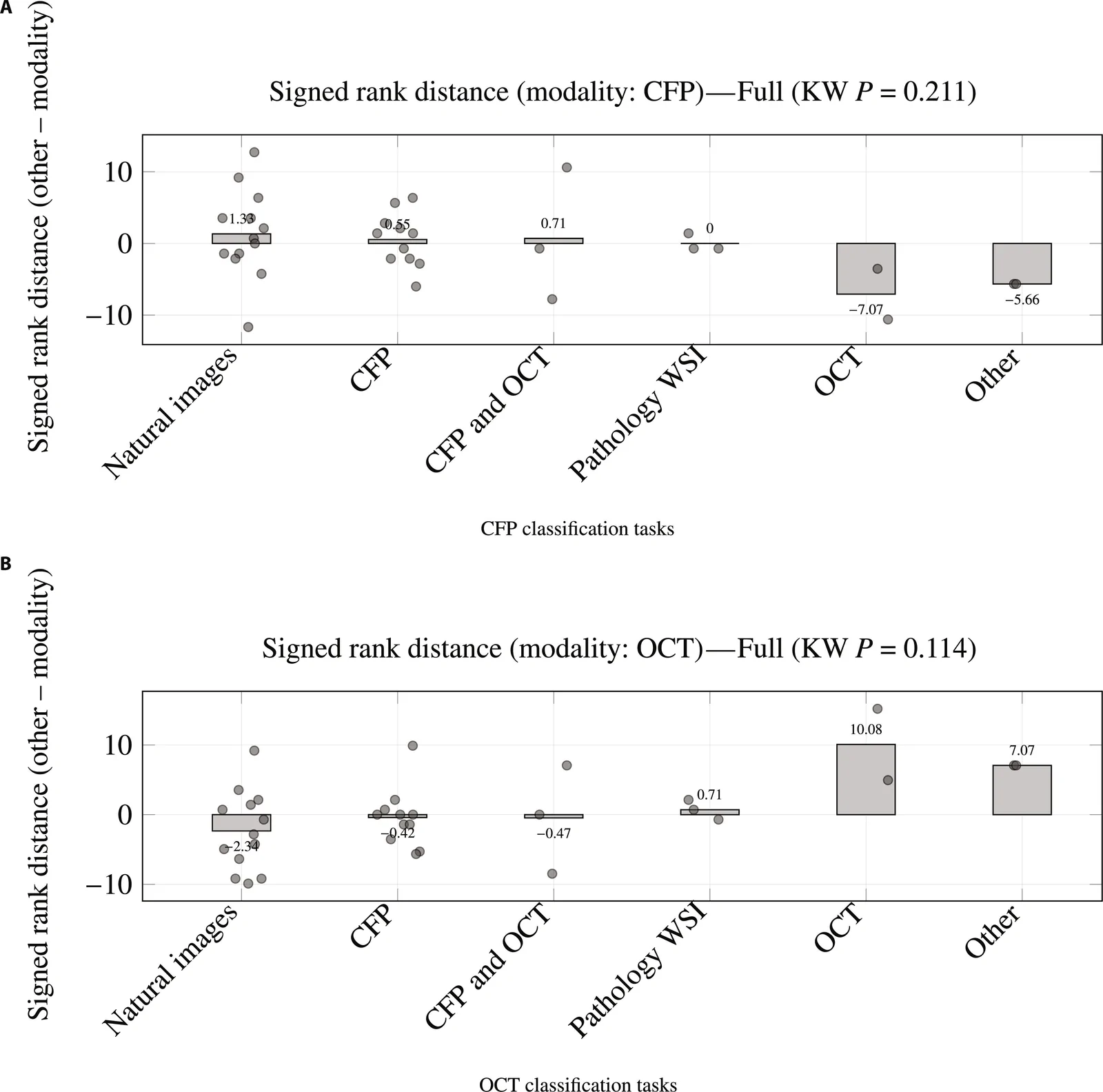
Signed rank distance grouped by pretraining domain, showing the relative performance on (A) color fundus photography (CFP) and (B) optical coherence tomography (OCT) tasks versus other modalities. A positive value indicates a better relative rank for the respective modality. Bars represent the mean signed rank distance for each pretraining group, with individual models overlaid as points. Groups are sorted by the number of models. A Kruskal–Wallis (KW) test assesses differences between pretraining domains.

Figure A.10 in Appendix A.4 shows the signed rank distance for each encoder when comparing performance for a specific modality against all other tasks (e.g., CFP versus rest). Figure 9 shows the resulting mean across encoders with the same pretraining modality. RGB-image pretraining appears to generalize to grayscale tasks, but not vice versa, as shown in Fig. 9. Models pretrained on grayscale images, such as the OCT models (RETFound MAE and VisionFM OCT) and the chest x-ray model (RadDINO), achieve a better relative rank on OCT classification tasks (Fig. A.10b). Conversely, the OCT-pretrained models rank lower when evaluated on CFP tasks (Fig. 9A), suggesting that their learned features do not generalize well to the CFP domain.

We conduct a similar analysis for tasks related to dr and glaucoma classification (Fig. A.11). For DR staging [74], Fig. A.11 shows that models pretrained with some form of self-distillation method demonstrate a slight performance advantage, although no single pretraining method is consistently superior. For glaucoma classification, CLIP-style pretrained models seem to struggle (Fig. A.11b).

To complement the modality-specific signed-rank-distance analysis, we additionally generated top 8 ranking plots under full fine-tuning for all tasks, CFP tasks only, and OCT tasks only (Fig. A.12), after excluding datasets with a high risk of data leakage as described in the “Full fine-tuning” section under “Controlling for potential data leakage”. Several patterns were notable (Fig. A.12). First, no encoder pretrained exclusively on OCT data ranked among the top 8 models in any of the 3 settings, including the OCT-only subset (Fig. A.12). Second, Virchow remained highly competitive across modalities and ranked first both in the overall analysis and in the OCT-only analysis, despite being pretrained on pathology whole-slide images rather than ophthalmic data (Fig. A.12). Third, several natural-image or broadly pretrained encoders, including DINOv2 variants and ViT-L/14 CLIP, appeared among the top-ranked models in several subsets, further supporting the observation that strong general-purpose visual representations can transfer effectively to ophthalmic classification tasks. Additionally, CFP-pretrained models such as RetiZero and DINORET ranked prominently for both CFP tasks and OCT tasks.

### Fairness

**Fig. 10.**
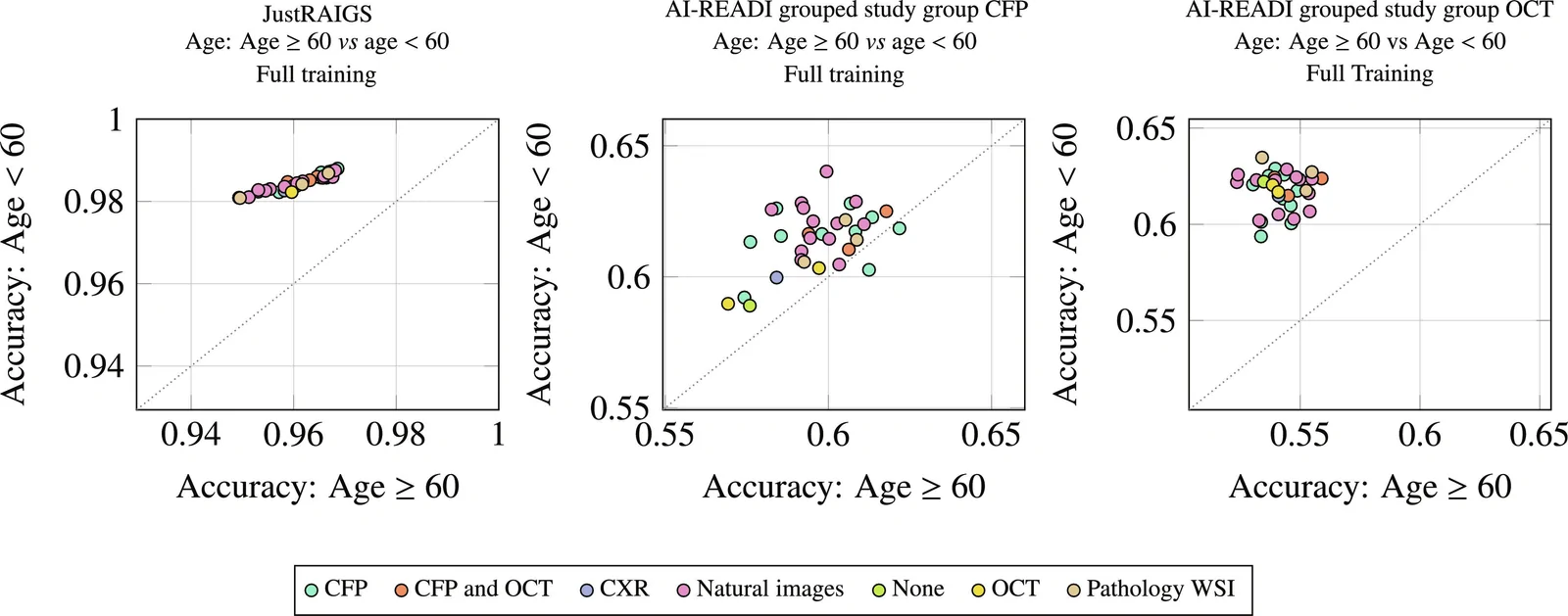
Model accuracy on 3 datasets stratified by age. Each point represents an encoder’s performance on the subgroup under 60 (*y*-axis) versus that for over 60 (*x*-axis). Colors indicate the pretraining domain, and individual dots represent the average differences across 3 replicates.

**Fig. 11.**
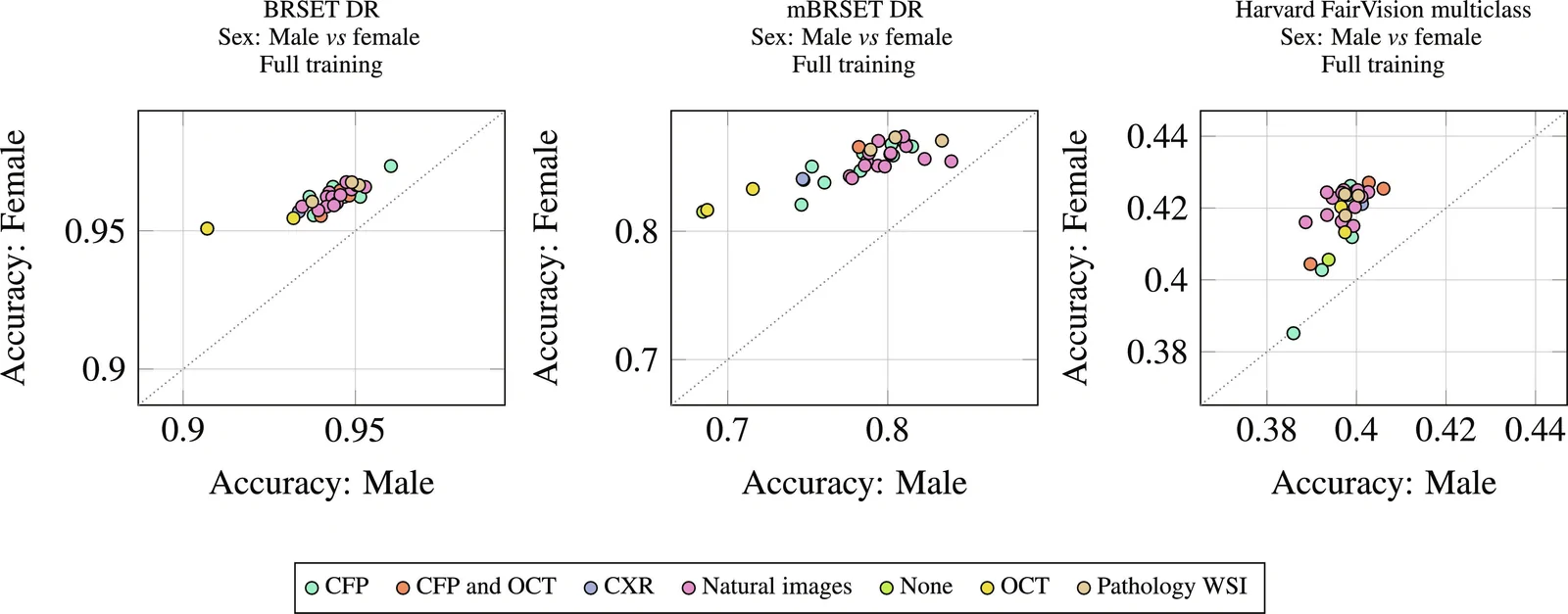
Model accuracy on 3 datasets stratified by sex. Each point represents an encoder’s performance on the female subgroup (*y*-axis) versus that on the male subgroup (*x*-axis). Colors indicate the pretraining domain, and individual dots represent the average differences across 3 replicates.

We assess model fairness by measuring performance disparities across demographic subgroups for datasets containing sensitive attributes. Figure 10 plots the classification accuracy for patients under 60 versus that for those over 60. Similarly, Fig. 11 shows that models perform better for the female subgroup, which is also the majority group in these datasets. The remaining datasets with fairness metrics are shown in Fig. A.13 in Appendix A.4. For both age and sex, the performance of all encoders is tightly clustered, mostly along a line. This indicates that the observed accuracy gap between subgroups is consistent across all encoders, without systematic differences in fairness outcomes based on model backbone selection.

### Learning rate sensitivity

**Fig. 12.**
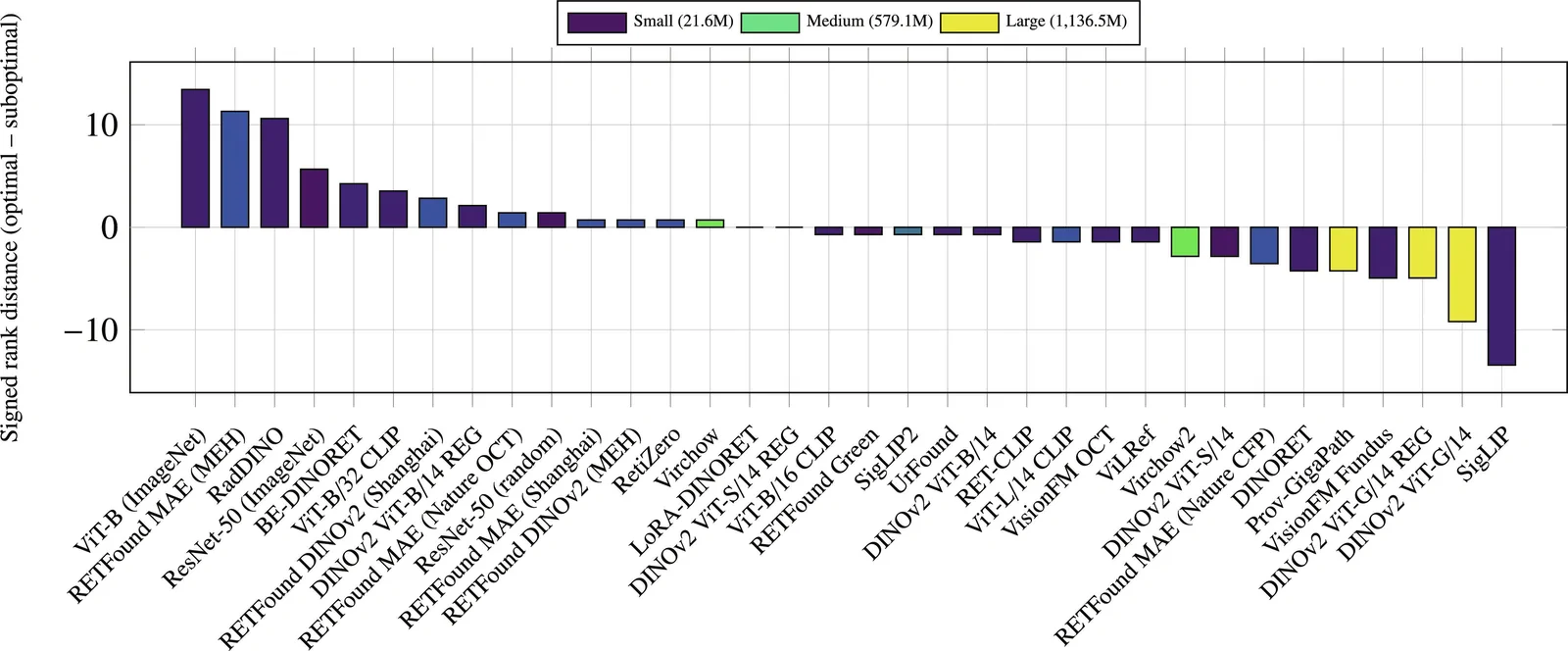
Signed rank distance showing the change in each encoder’s rank between using an optimal learning rate and the mean of suboptimal learning rates for the task of diabetic retinopathy (DR) classification of the color fundus photography (CFP) images of the AI-READI dataset. A positive value indicates better relative robustness to suboptimal learning rates. The bars are colored by model size. Models are listed from left to right by decreasing signed rank distances.

When fine-tuning models for a specific task, a wide range of hyperparameter settings can be chosen, chiefly learning rates for the vision encoder and the classification head. Consistent outcomes under different hyperparameter settings could indicate model robustness and allow fine-tuning for specific tasks with minimal effort. We thus evaluate the robustness of each encoder to hyperparameter selection by comparing its performance with an optimal learning rate against its average performance across a range of suboptimal learning rates for DR classification on CFP images from the AI-READI dataset. Figure 12 illustrates the change in mean rank between these conditions.

We observed an association between model size and learning rate sensitivity. The 3 largest models (Prov-GigaPath, DINOv2 ViT-G/14, and DINOv2 ViT-G/14 REG) exhibit large negative rank changes, meaning their relative performance degrades more with suboptimal learning rates. In contrast, smaller baseline models like ResNet-50 and ViT-B (ImageNet) show positive rank changes, performing more stably even with less precise hyperparameter tuning.

Performing a full learning rate grid search for every encoder–dataset combination is computationally infeasible. As a sensitivity analysis, we therefore investigate the transferability of the identified learning rates by repeating the learning rate sweep on a second dataset, EyePACS balanced, for a subset of representative models. Specifically, we selected ViT-B (ImageNet), DINOv2 ViT-B/14, and SigLIP, which exhibited the most positive rank change, the most negative rank change, and a near-zero rank change, respectively, in Fig. 12. As shown in Figs. A.14 and A.15, encoders exhibit a preference for similar classification head and encoder learning rates across both tasks. Learning rates yielding the best performance on the AI-READI dataset also exhibit good performance on the EyePACS balanced dataset.

### Compute requirements

Beyond predictive performance, the practical use of foundation models in ophthalmology also depends on computational efficiency. We therefore benchmarked the GPU memory footprint and runtime of each encoder on the APTOS DR classification task under both full fine-tuning and linear probing. Training benchmarks were performed with a batch size of 64 and 25 steps per epoch, while inference benchmarks were measured on the test split with a batch size of 1 after a short warm-up phase. All experiments were performed on GH200. The resulting inference memory and latency comparisons are shown in Figs. A.16 and A.17, while the corresponding training memory and runtime comparisons are shown in Figs. A.18 and A.19.

## Discussion

Our large-scale benchmarking of over 30 vision encoders across 39 ophthalmic classification tasks highlights both the promise and current limitations of applying foundation models to ophthalmology. The results reveal a landscape of high variability, nuanced domain transfer, and substantial sensitivity to evaluation design, raising broader questions about how generalizable visual representations truly are for retinal imaging [11,26].

A central observation is the pronounced task dependence of model performance. Even the top-performing encoder, Virchow [22], failed to generalize consistently and ranked among the worst-performing encoders for several tasks. Conversely, some encoders with modest overall rankings excelled in specific challenges. This variability underscores that success on a given ophthalmic dataset may reflect task-specific alignment rather than general transfer capability [11,79]. It also illustrates the instability of leaderboard-style comparisons: performance hierarchies shift depending on data modality, disease category, and label granularity.

Interestingly, our findings suggest that cross-domain pretraining can outperform in-domain learning. Models such as Virchow [22], pretrained on histopathology whole-slide images, transferred surprisingly well to retinal images, outperforming both general-purpose and ophthalmology-specific encoders. While part of this success may stem from Virchow’s DINOv2 backbone, its added benefit over baseline DINOv2 models may also reflect properties of histopathology pretraining that are useful beyond the histological domain. Whole-slide images expose models to highly diverse local morphology across multiple spatial scales, including fine-grained textures, vascular structures, tissue boundaries, and small abnormal patterns. Such pretraining may therefore encourage sensitivity to local structural detail, a property that is also important for retinal classification tasks involving vessels, hemorrhages, exudates, and other subtle lesions. In contrast, modality-matched CFP pretraining may provide closer image-level domain alignment but less diversity in scale, tissue appearance, and local morphological variation. This interpretation remains speculative, but it provides one possible explanation for why histopathology-pretrained representations transferred unexpectedly well to CFP tasks. Together, these findings suggest that domain-specific pretraining is not inherently superior and ophthalmic performance can benefit from out-of-domain pretraining [39]. In contrast, SigLIP2 [45], despite being one of the largest and most recent models with extensive pretraining data, did not perform well, reinforcing that scale and recency alone do not ensure better transfer.

The limited advantage of in-domain pretraining is another striking result. Ophthalmic encoders trained on CFP or OCT data did not consistently outperform large, natural-domain models. Natural-domain models often matched or even exceeded the performance of domain-specific models, indicating that exposure to a wide variety of visual semantics may yield more robust and flexible representations than narrow, modality-specific learning [39]. At the same time, benchmark composition can influence perceived generalization. In particular, the overall underperformance of OCT-based encoders may partly reflect the predominance of CFP tasks in our benchmark. However, the modality-stratified analysis supports the same overall conclusion. Even when evaluation was restricted to CFP or OCT tasks, the highest-ranked encoders were not simply those pretrained on the matching ophthalmic modality. No exclusively OCT-pretrained encoder appeared among the top 8 models, even for OCT-only tasks, whereas Virchow ranked first overall and within the OCT subset and second within the CFP subset. Conversely, several encoders pretrained on CFP, natural images, or histopathology images ranked competitively across modality-restricted analyses. These findings suggest that modality-matched pretraining alone is insufficient to guarantee strong transfer and that broader representation quality, pretraining scale, and self-supervised learning objectives may be at least as important as nominal domain alignment.

Evaluation methodology also played a critical role in shaping conclusions. The only moderate correlations between linear probing and full fine-tuning emphasize that proxy evaluations provide unreliable signals of ultimate performance [27,80]. While linear probing remains attractive for its computational efficiency, it fails to capture the entire fine-tuning dynamics [27,80]. Consequently, simplified evaluation pipelines risk misleading conclusions about model superiority and should be complemented by controlled fine-tuning experiments. Interestingly, we show that class-balancing training datasets only minimally changes relative rankings and performance outcomes, indicating that data balancing may not always be necessary for future evaluations and that models can perform well, even if fine-tuned on largely imbalanced datasets.

Our fairness analysis further illustrates the complexity of benchmarking foundation models. Although modest performance gaps appeared across sex and age subgroups, these disparities were broadly consistent across all encoders, suggesting that model architecture alone is unlikely to explain the observed subgroup differences. Instead, such disparities may reflect biases already present in the downstream datasets [81], as well as biases in the fine-tuning datasets or in the clinical distributions used for evaluation [69,82,83]. While developing fairness-specific training strategies was beyond the scope of this benchmark, future work should assess whether balanced sampling, subgroup-aware calibration, reweighting strategies, fairness-aware loss functions, or more deliberate pretraining data curation can mitigate these gaps consistently across a broad range of foundation models [69,82,83]. Still, fully unbiased pretraining and fine-tuning may remain difficult with currently available ophthalmic datasets, which often lack complete demographic metadata, exhibit substantial class imbalance, and were not originally designed to provide balanced representation across clinically relevant subgroups [69].

From a practical perspective, our findings suggest that encoder selection in ophthalmology should be guided by the intended deployment setting rather than by aggregate benchmark rank alone. Because strong average performance did not guarantee consistent task-level superiority, candidate models should be evaluated on the specific target dataset while also considering inference latency, memory requirements, training cost, reproducibility, and ease of deployment. Our released benchmark pipeline facilitates this process by allowing users to test selected encoders on their own datasets and identify suitable base models for a given task. This is particularly relevant for low-resource research settings, clinical translation, and regulatory-facing development, where model choice should also consider robustness, transparent documentation of pretraining exposure, and prospective validation in the intended clinical population.

Despite its scale and diversity, our study has several limitations. The benchmark’s imbalance toward CFP and OCT tasks constrains the generalizability of findings to less common ophthalmic imaging modalities, such as ultrasound [19]. This imbalance reflects the current availability of public ophthalmic datasets and may disadvantage models pretrained primarily on underrepresented modalities, although we partially addressed this issue through modality-stratified analyses. Future benchmarks would ideally include a more balanced distribution of CFP, OCT, SLO, and other ophthalmic imaging modalities while continuing to report modality-specific performance. Large private or institutional datasets could help achieve such balance and reduce the risk of pretraining data leakage; however, they would also limit reproducibility and independent reuse by other researchers. We therefore prioritized publicly available datasets and a unified benchmarking pipeline, providing a transparent resource that can be extended as additional public datasets and foundation models become available.

Another limitation is that, while we carefully screened for potential data leakage, residual overlap cannot be completely ruled out given the widespread reuse of public datasets in pretraining pipelines [19,25]. Nevertheless, the strong performance of models pretrained on natural or histopathology images, domains with no direct overlap with ophthalmic data, supports the robustness of our conclusions [39]. Moreover, our sensitivity analysis, restricting the benchmark to tasks that are unlikely to appear in web-scale pretraining corpora, did not suggest any systematic bias introduced by potential data leakage from directly scrapeable datasets. A further limitation is inherently imposed by the rapid pace of the field. Our benchmark includes 34 encoders that were available when the experimental pipeline was initiated, but several promising models, including MIRAGE [84] and DINOv3 [85], were released after the main experiments had started or concluded. These models should be evaluated in future extensions of this benchmark, which are facilitated by the pipeline we provide. Our work should therefore be interpreted as a systematic snapshot of model performance at the time of experimentation and as a resource for further benchmarking, rather than as a final ranking of all available foundation models.

Finally, by enforcing uniform fine-tuning protocols, we prioritized reproducibility and comparability across models, but this choice may have disadvantaged certain architectures or models that benefit from specialized optimization strategies. In particular, for transformer-based models, we relied on the CLS token for classification. While this provides a consistent representation across encoders, it may not be optimal for all architectures, as informative features may also be distributed across patch tokens. Alternative strategies, such as averaging or concatenating patch-token embeddings, may improve individual model performance and can yield slightly different results [23]. Future work should therefore explore model-specific pooling and fine-tuning strategies while balancing performance optimization against the need for standardized comparison.

Taken together, these findings suggest that the success of foundation models in ophthalmology is shaped less by scale or domain specificity and more by the interplay between task characteristics, dataset composition, and fine-tuning strategy. Our results reveal that natural-domain and histopathology-pretrained encoders can transfer remarkably well to retinal imaging, sometimes rivaling or surpassing models trained exclusively on ophthalmic data [23,26,39,50]. This indicates that rich, diverse visual representations—rather than narrow domain focus—may provide the most adaptable foundation for retinal imaging. Future work should explore hybrid or joint pretraining paradigms that combine natural, histopathological, and ophthalmic data to capture both generic and disease-relevant visual features. As the field advances toward clinical translation, benchmarking must move beyond single-number rankings to emphasize reliability, fairness, and interpretability, ensuring that foundation models become not just powerful, but truly trustworthy tools in ophthalmic care [11,32,33,37].

## Conclusion

In conclusion, we present the first large-scale, standardized benchmark of vision encoders for ophthalmic image classification, spanning 34 models and 39 classification tasks across 21 public datasets. Our results reveal that no single encoder dominates across tasks—performance is strongly task and modality dependent, with natural- and histopathology-pretrained models often rivaling or surpassing ophthalmology-specific ones. Proxy metrics such as linear probing and kNN only moderately predict fine-tuning outcomes, underscoring the need for comprehensive evaluations in model selection.

By releasing our open-source benchmarking framework, we aim to provide a transparent, reproducible foundation for future research. Beyond ranking models, this work establishes a reality check for foundation models in ophthalmology and charts a path toward more reliable, fair, and clinically meaningful applications of AI in eye care.

## Data Availability

Code and scripts are available at https://github.com/cm090999/ocularbench.git. Dataset splits and evaluation configuration will be made available at the same URL. The public datasets used in this study are available from their original sources (see Methods/Appendix for citations and access conditions).

## Supplementary Materials

Supplementary 1 Appendices A.1 to A.5 Figs. A.1 to A.19 Tables A.1 to A.21
