# Supplementary material for: Benchmarking Vision Encoders for Image Classification in Ophthalmology
Source: Comput Struct Biotechnol J. 2026 Sep 1;36(2):0178. doi: 10.34133/csbj.0178 (PMC13530374; doi:10.34133/csbj.0178)
Supplement: Supplementary file 1 [file csbj.0178.f1.pdf]

Supplementary Material

Benchmarking Vision Encoders for Image Classification in Ophthalmology

Jay Zoellin[1,*], Colin Merk[1,*], Bence György[1,2,†]

[1]Institute of Molecular and Clinical Ophthalmology Basel (IOB)

[2]Department of Ophthalmology, University of Basel, Basel, Switzerland

[*]These authors contributed equally and share first authorship.

[†]Corresponding author: bence.gyoergy@iob.ch

Appendix A. Appendix

Appendix A.1. Compute and Timeline

All experiments, including the learning rate grid search, full fine-tuning, and linear probing of all encoders across all datasets, each with three random seeds, required approximately 28 days of computation on a single GH200 instance. Training runs were initiated in early June 2025; consequently, models released after this date were not considered in the experiments.

Appendix A.2. Vision Encoders

Table A.1: Vision encoders evaluated in this study, grouped by pretraining data type and pretraining method.

**Model**	**Pretraining Data Type**	**Pretraining Method**
BE-DINORET, LoRA-DINORET, DINORET	Natural Images, CFP	Self-distillation (DINO)
DINOv2 ViT-S/14, DINOv2 ViT-B/14, DINOv2 ViT-G/14	Natural Images	Self-distillation (DINO)
DINOv2 ViT-S/14 REG, DINOv2 ViT-B/14 REG, DINOv2 ViT-G/14 REG	Natural Images	Self-distillation (DINO)
Prov-GigaPath	Pathology Whole Slide Images (WSI)	Masked Autoencoder (MAE)
RadDINO	Natural Images, Chest X-rays	Self-distillation (DINO)
ResNet-50 (ImageNet)	Natural Images	Supervised (ImageNet)
ResNet-50 (Random)	None	Untrained
RET-CLIP	CFP	Contrastive (CLIP-style)
RETFound DINOv2 (MEH), RETFound DINOv2 (Shanghai)	Natural Images, CFP, OCT, FAF, Red-Free CFP / Natural Images, CFP	Self-distillation (DINO)
RETFound MAE (MEH), RETFound MAE (Nature CFP), RETFound MAE (Nature OCT), RETFound MAE (Shanghai)	Natural Images, CFP, OCT, FAF, Red-Free CFP / Natural Images, CFP / Natural Images, OCT	Masked Autoencoder (MAE)
RETFound Green	Natural Images, CFP	Masked Autoencoder (MAE)
RETIZero	CFP	Contrastive (CLIP-style)
SigLIP	Natural Images	Contrastive (CLIP-style)
SigLIP2	Natural Images	Contrastive (CLIP-style)
URFound	CFP, OCT	Masked Autoencoder (MAE), Contrastive (CLIP-style)
ViLRef	CFP	Contrastive (CLIP-style)
Virchow	Natural Images, Pathology WSI	Self-distillation (DINO)
Virchow2	Natural Images, Pathology WSI	Self-distillation (DINO)
VisionFM Fundus	CFP	Self-distillation (DINO)
VisionFM OCT	OCT	Self-distillation (DINO)
ViT-B/16 CLIP, ViT-B/32 CLIP, ViT-L/14 CLIP	Natural Images	Contrastive (CLIP-style)
ViT-B (ImageNet)	Natural Images	Supervised (ImageNet)

Appendix A.3. Datasets

We use 21 publicly available datasets to construct 39 classification tasks. If datasets provided predefined training and test splits, we pooled the data to enable evaluation with multiple independently sampled splits. For all experiments, we created stratified train–validation–test partitions of 70 %, 15 %, and 15 %. When participant identifiers were available, we used them for stratified splits to avoid patient-level data leakage. To reduce disk I/O overhead and enable consistent model input, we resized all images to 224×224 pixels. A detailed breakdown of all classification tasks, including scripts and dataset setup instructions, is available in our code repository.

Appendix A.3.1. Multi-Disease Datasets

Harvard FairVision (Multi-Class). The *Harvard FairVision Dataset* contains 30,000 3D OCT scans. We use the central B-scan of each scan and generate four disease classes based on the provided diagnostic labels: ("Healthy"), ("Diabetic Retinopathy"), ("Glaucoma"), and ("Age-related Macular Degeneration"). Risk for data leakage: No. Definitive data leakage: None.

RFMiD (Multi-Class). The *Retinal Fundus Multi-Disease Image Dataset* consists of 3,200 CFPs covering 46 conditions. Following previous work, we excluded multi-label images and conditions with fewer than 20 images, yielding 2,303 images across 16 single-label classes. Risk for data leakage: Yes. Definitive data leakage: RetiZero, URFound.

RFMiD (Binary). To mitigate severe class imbalance and support a simpler setting, we defined a binary disease task distinguishing ("Healthy") from ("Abnormal") images yielding 2,303 images across 2 single-label classes. Risk for data leakage: Yes. Definitive data leakage: RetiZero, URFound.

Open UWF. The *Open Ultra-widefield Fundus Image Dataset* contains 700 ultra-widefield fundus photographs spanning seven classes: ("Diabetic Retinopathy"), ("Age-related Macular Degeneration"), ("Retinal Vein Occlusion"), ("Pathological Myopia"), ("Uveitis"), ("Retinal Detachment"), and ("Healthy"). Risk for data leakage: Yes. Definitive data leakage: No.

OCTID. The *Optical Coherence Tomography Image Database* consists of 572 OCT central B-scans labeled as ("Healthy"), ("Age-related Macular Degeneration"), ("Diabetic Retinopathy"), ("Macular Hole"), or ("Central Serous Chorioretinopathy"). Risk for data leakage: Yes. Definitive data leakage: No.

HRF. The *High-Resolution Fundus (HRF) Database* contains 45 CFPs, originally designed for vessel segmentation. As it includes disease labels ("Healthy"), ("Diabetic Retinopathy"), and ("Glaucoma"), we use them for a three-class classification task. Risk for data leakage: Yes. Definitive data leakage: No.

OCT-XRAY. The *OCT-XRAY Dataset* contains 84,484 OCT central B-scans labeled as ("Choroidal Neovascularization"), ("Diabetic Macular Edema"), ("Drusen"), or ("Healthy"). We use these labels for disease classification. Risk for data leakage: Yes. Definitive data leakage: No.

Appendix A.3.2. Diabetic Retinopathy

We evaluate diabetic retinopathy (DR) staging according to the International Clinical Diabetic Retinopathy (ICDR) scale.

APTOS2019. The *Asia Pacific Tele-Ophthalmology Society Dataset* consists of 3,662 macula-centered fundus photographs with ICDR labels. Because test labels are not provided, we only use the training and validation partitions of the dataset. Risk for data leakage: Yes. Definitive data leakage: RETIZero.

DRTiD. The *Diabetic Retinopathy Two-field Image Dataset* contains 1,550 macula-centered fundus photographs. Labels were assigned by three ophthalmologists and adjudicated by a senior ophthalmologist. We retained one macula-centered image per eye. Risk for data leakage: Yes. Definitive data leakage: No.

EYEPACS. The *EYEPACS Dataset* includes 88,702 CFPs acquired using multiple camera systems, with one image per eye and ICDR labels. Risk for data leakage: Yes. Definitive data leakage: DINORET, BE-DINORET, RETIZero, URFound.

EYEPACS (Balanced). To enable a class-balanced evaluation, we constructed a balanced subset by downsampling each label to the same sample count, resulting in 9,570 images. Risk for data leakage: Yes. Definitive data leakage: DINORET, BE-DINORET, RETIZero, URFound.

IDRiD (DR). The *Indian Diabetic Retinopathy Image Dataset* contains 516 CFPs labeled for ICDR staging. Risk for data leakage: Yes. Definitive data leakage: RETIZero.

BRSET (DR). The *Brazilian Multilabel Ophthalmological Dataset (BRSET)* consists of 16,266 CFPs from 8,524 patients, including clinical metadata. We use provided DR severity labels for ICDR staging. Risk for data leakage: No. Definitive data leakage: No.

mBRSET (DR). The *Mobile BRSET Dataset* contains 5,164 fundus images acquired using portable cameras from 1,291 diabetic patients. We use the DR severity labels for ICDR staging. Risk for data leakage: No. Definitive data leakage: No.

Messidor (DR). Messidor includes fundus photographs annotated for DR. Risk for data leakage: Yes. Definitive data leakage: RETIZero.

Appendix A.3.3. Glaucoma

We use several publicly available glaucoma datasets. The classes are ("Healthy") and ("Glaucoma"). The PAPILA dataset provides an additional class ("Suspicious").

PAPILA. The *PAPILA Dataset* contains 488 CFPs of healthy and glaucoma patients, with demographic information including age and sex. Risk for data leakage: Yes. Definitive data leakage: No.

Harvard FairGenMed (Glaucoma). The *Harvard FairGenMed Dataset* contains scanning laser ophthalmoscopy (SLO) images with demographic metadata. We use the glaucoma classification task. Risk for data leakage: No. Definitive data leakage: No.

Harvard FairFedMed (Glaucoma). The *Harvard FairFedMed Dataset* contains paired 3D OCT and SLO images from three clinical sites. We use the central B-scan for glaucoma classification. Risk for data leakage: No. Definitive data leakage: No.

Harvard GF (Glaucoma). The *Harvard Glaucoma Fairness Dataset* contains 3D OCT data with glaucoma labels. We extract the central B-scan for classification. Risk for data leakage: No. Definitive data leakage: No.

Harvard FairVision (Glaucoma). The *Harvard FairVision Dataset* provides 10,000 3D OCT scans labeled as ("Healthy") or ("Glaucoma"). We use the central B-scan. Risk for data leakage: No. Definitive data leakage: No.

JustRAIGS. The *Justified Referral in AI Glaucoma Screening (JustRAIGS)* dataset contains CFP images covering ("Referable") and ("Non-Referable Glaucoma"). Risk for data leakage: Yes. Definitive data leakage: RETIZero.

Appendix A.3.4. Other Tasks

IDRiD (ME). From IDRiD, we use labels for the presence of ("Macular Edema") as a binary classification task. Risk for data leakage: Yes, Definitive data leakage: RETIZero.

Messidor (DME). We use the ("Diabetic Macular Edema") labels from Krause et al. on the Messidor dataset. Risk for data leakage: Yes. Definitive data leakage: RETIZero.

BRSET (Hypertension). Using BRSET metadata, we classify the presence of ("Systemic Hypertension"). Risk for data leakage: No. Definitive data leakage: No.

mBRSET (Hypertension / Obesity / Microvascular). From mBRSET, we define three tasks to predict ("Systemic Hypertension"), ("Obesity"), and ("Microvascular Pathologies") from CFPs. Risk for data leakage: No. Definitive data leakage: No.

FIMD. The *Fundus Image Myopia Dataset* includes images with future visual acuity measurements over 2–5 years. We define a binary task predicting above- vs. below-median visual acuity change. Risk for data leakage: Yes. Definitive data leakage: No.

GRAPE (PLR2 / PLR3 / MD). The GRAPE dataset provides longitudinal fundus imaging with visual field progression labels. We evaluate three binary tasks: PLR2 and PLR3, based on pointwise linear regression (at least two or three points with $p < 0.01$ negative slopes), and MD, based on significant mean deviation decline ($p < 0.05$). Risk for data leakage: Yes. Definitive data leakage: No.

Harvard GF (MD). We predict visual field mean deviation (MD) from the central B-scan. Risk for data leakage: No. Definitive data leakage: No.

Harvard FairGenMed (Vision Loss). We predict future vision loss from SLO images. Risk for data leakage: No. Definitive data leakage: No.

Harvard FairVision (AMD). We classify ("Age-Related Macular Degeneration severity") (no, early, intermediate, late AMD) using central B-scans. Risk for data leakage: No. Definitive data leakage: No.

Harvard FairVision (DR). We classify central B-scans as ("Vision-threatening") or ("Non–Vision-threatening") diabetic retinopathy. Risk for data leakage: No. Definitive data leakage: No.

AI-READI (CFP). We use the AI-READI dataset to classify color fundus photographs into four study groups reflecting metabolic status: ("Healthy"), ("Pre-diabetes (lifestyle controlled)"), ("Oral/Non-insulin injectable medication controlled"), and ("Insulin dependent"). Risk for data leakage: No. Definitive data leakage: No.

AI-READI (OCT). Analogously, we classify the central B-scan from 3D OCT volumes into the same four metabolic study groups, using the central B-scan from each volume for training and evaluation. Risk for data leakage: No. Definitive data leakage: No.

AI-READI (CFP, Grouped). For binary classification, ("Healthy") and ("Pre-diabetes") are grouped as ("Non-diabetic"), while the two medication-controlled categories form the ("Diabetic") class. Risk for data leakage: No. Definitive data leakage: No.

AI-READI (OCT, Grouped). The same binary grouping is applied to OCT-based classification, using central B-scans to distinguish ("Non-diabetic") from ("Diabetic") participants. Risk for data leakage: No. Definitive data leakage: No.

Appendix A.4. Additional Results

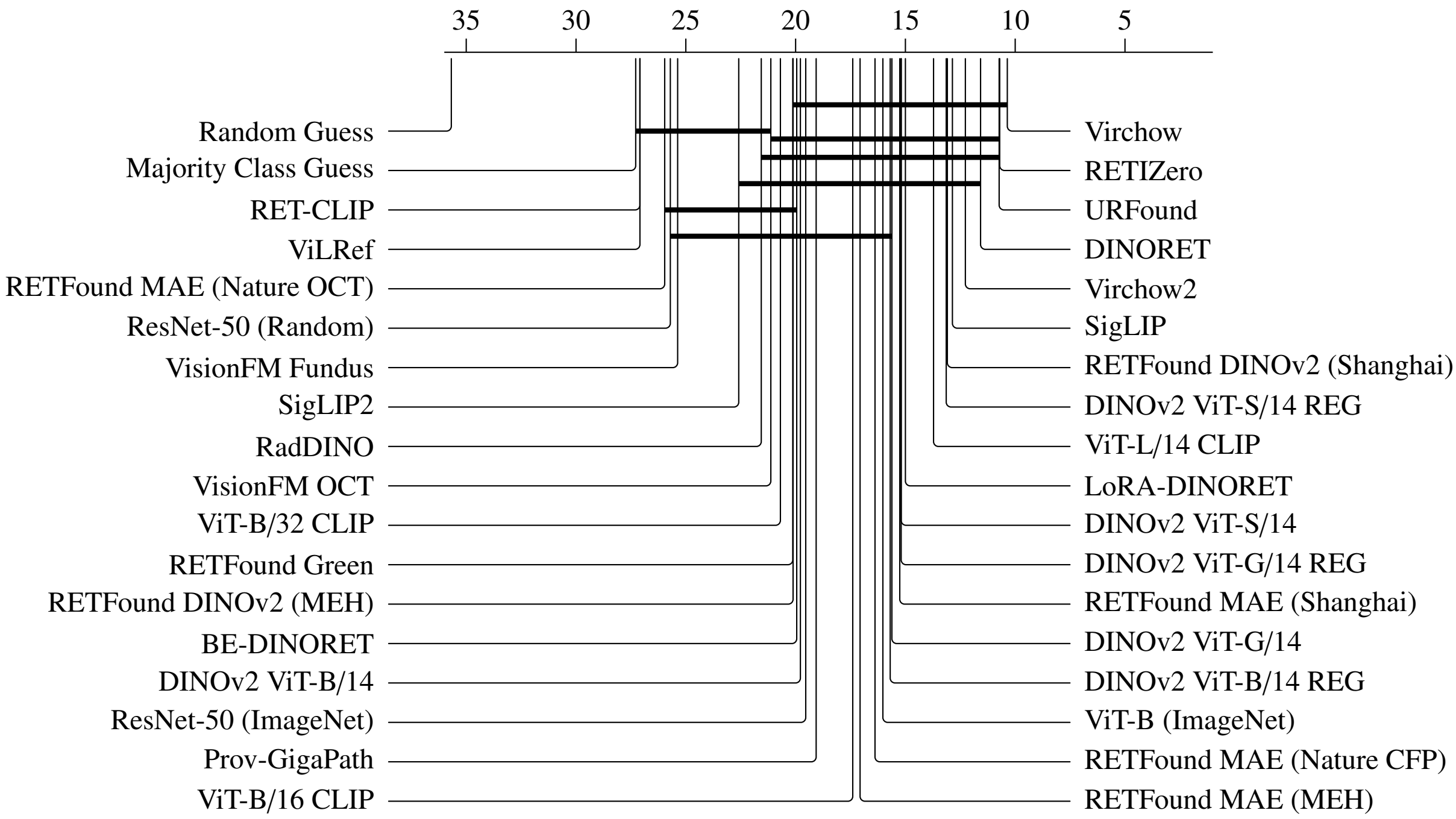

Figure A.1: A critical difference plot of model rankings for full fine-tuning based on mean ranks across tasks. Models connected by horizontal bars form groups that do not differ significantly according to the Nemenyi post-hoc test (α = 0.05). Lower ranks indicate better performance. The red bar indicates the critical difference (CD). The corresponding Friedman test p-value is $p < 0.001$ and the corresponding Kendall's W is 0.322.

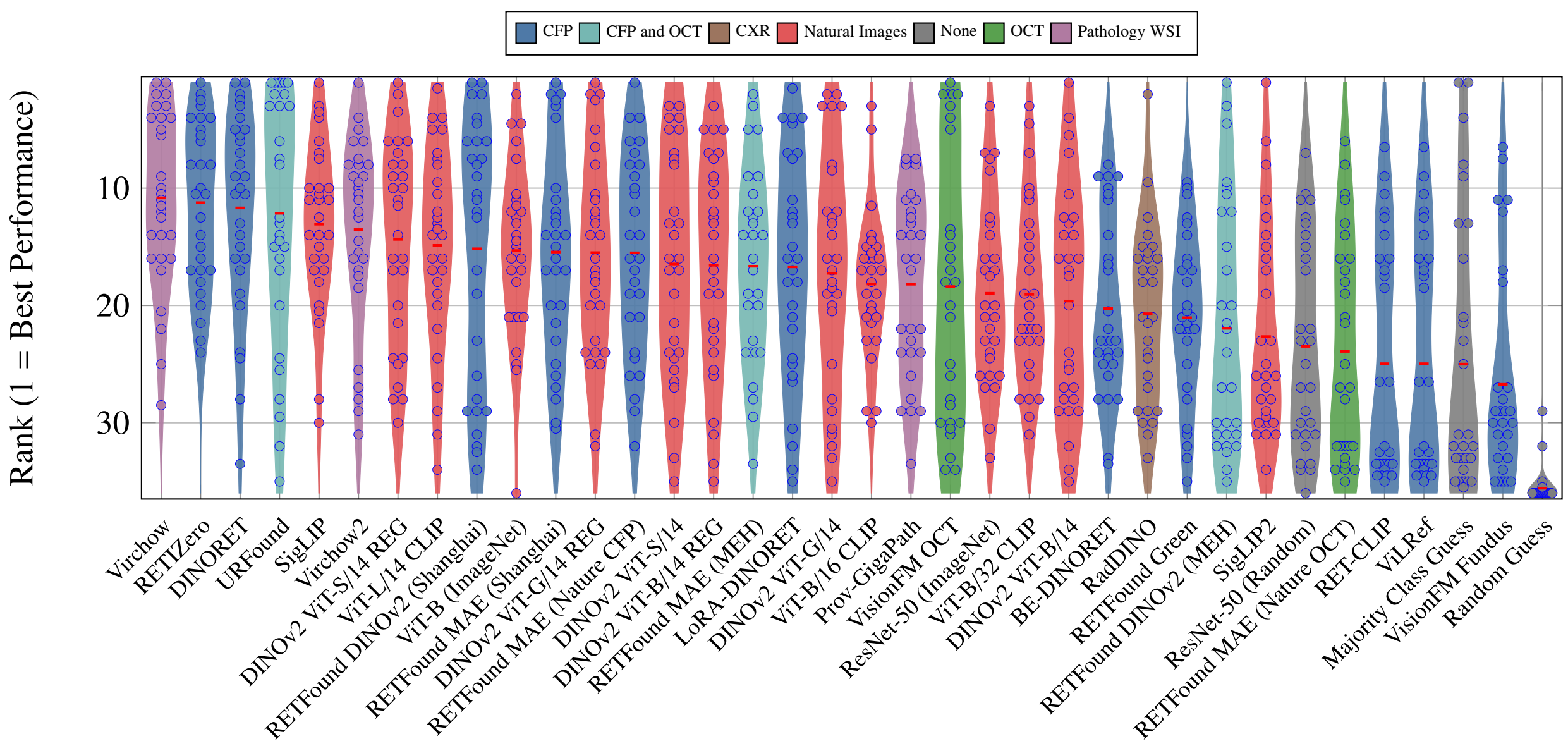

Figure A.2: This violin plot shows the rank distribution for full fine-tuning of the tested encoders across 29 tasks after removing datasets with documented data leakage for one or more encoders. Each individual rank is based on the average test accuracy across 3 differently seeded runs. The colors of the violin plot indicate the pretraining domain for each model.

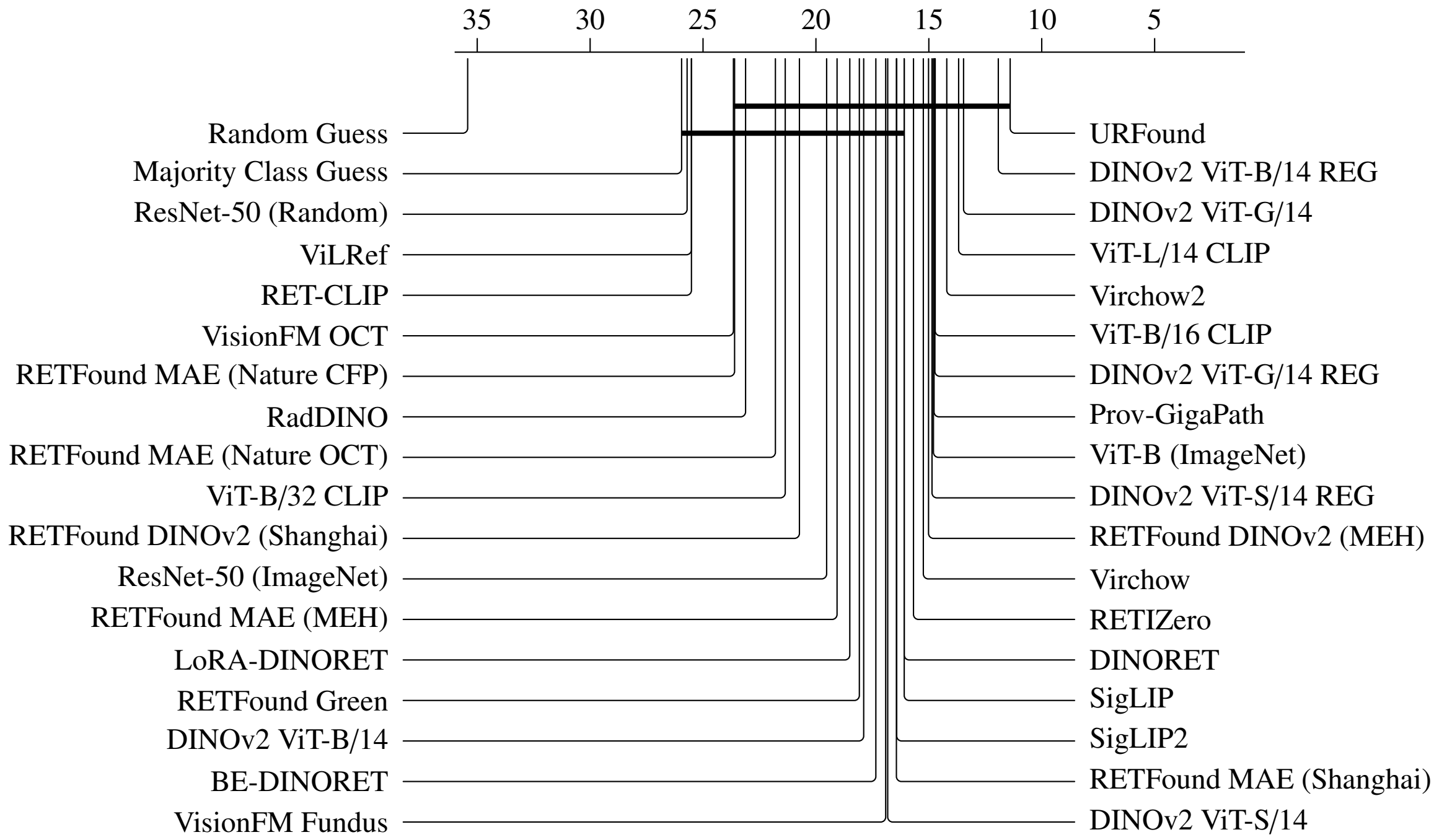

Figure A.3: A critical difference plot of model rankings for linear probing evaluations based on mean ranks across tasks. Models connected by horizontal bars form groups that do not differ significantly according to the Nemenyi post-hoc test ($\alpha = 0.05$). Lower ranks indicate better performance. The red bar indicates the critical difference (CD). The corresponding Friedman test p-value is $p < 0.001$ and the corresponding Kendall's W is 0.235.

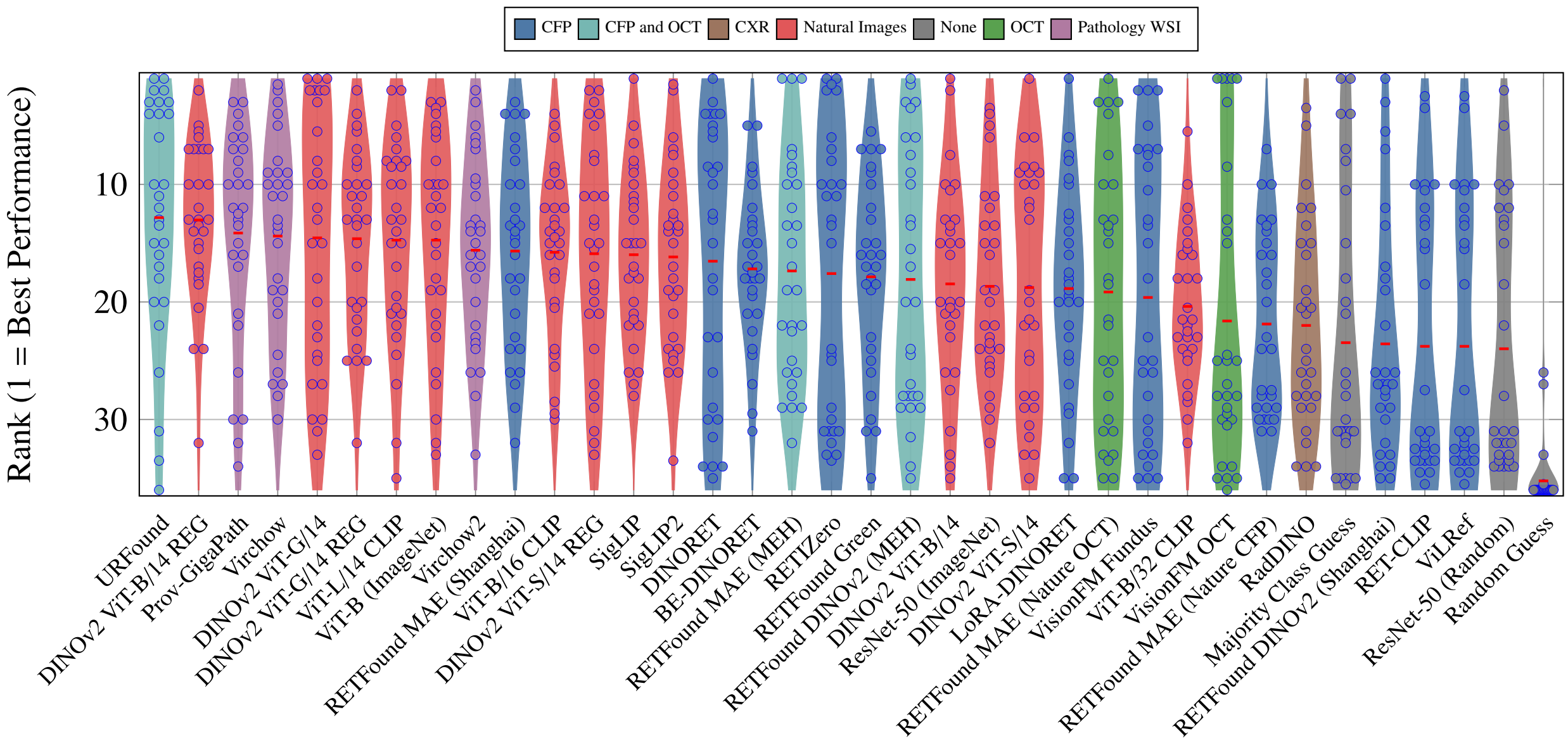

Figure A.4: This violin plot shows the rank distribution for linear probing of the tested encoders across 29 tasks after removing datasets with known data leakage for one or more encoders. Each individual rank is based on the average test accuracy across 3 differently seeded runs. The colors of the violin plot indicate the pretraining domain for each model.

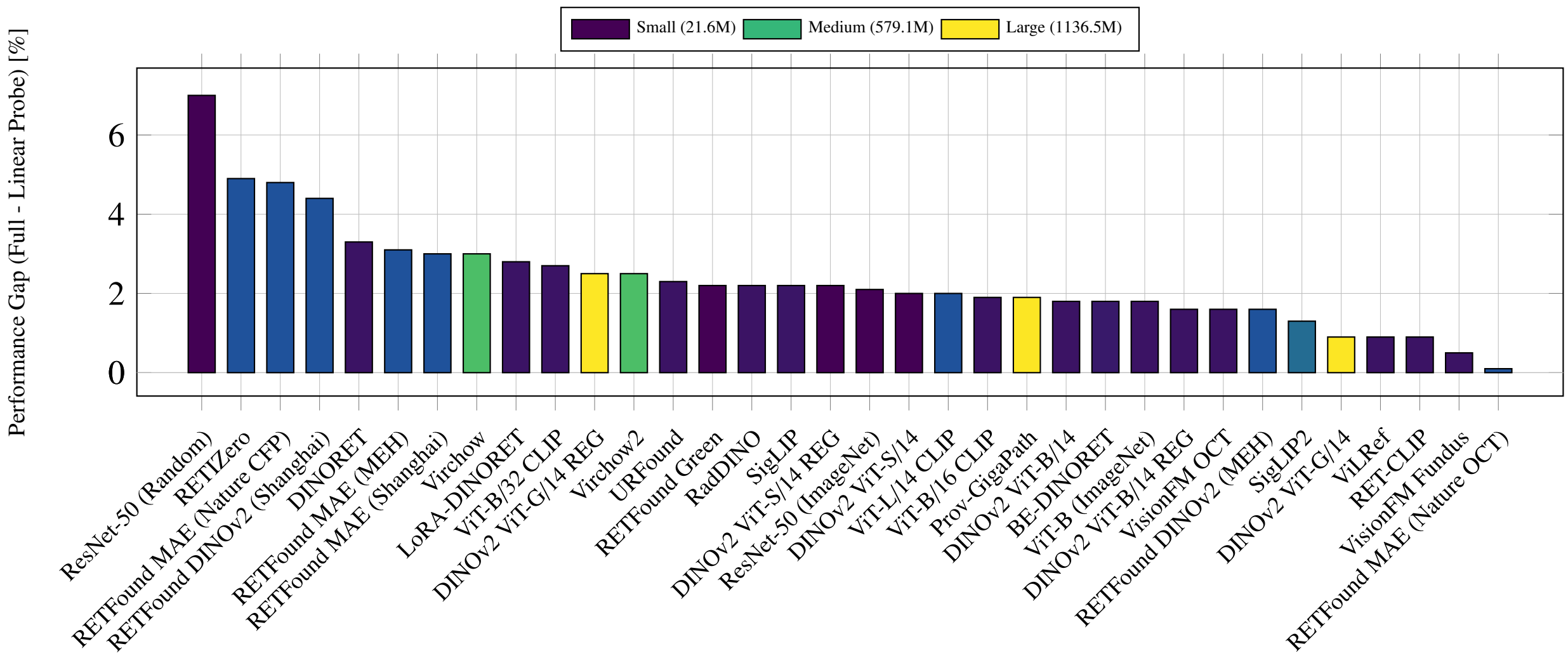

Figure A.5: Performance gap between full fine-tuning and linear probing, calculated as the difference in mean accuracy across all datasets. A larger gap indicates a greater benefit from fine-tuning the full network. Encoders are listed by decreasing gaps from left to right and a color gradient indicates model size (number of total parameters, from small in violet to large in yellow, with 3 reference sizes annotated). Results shown represent the mean accuracy gap across all datasets, with three replicates per dataset using random seeds.

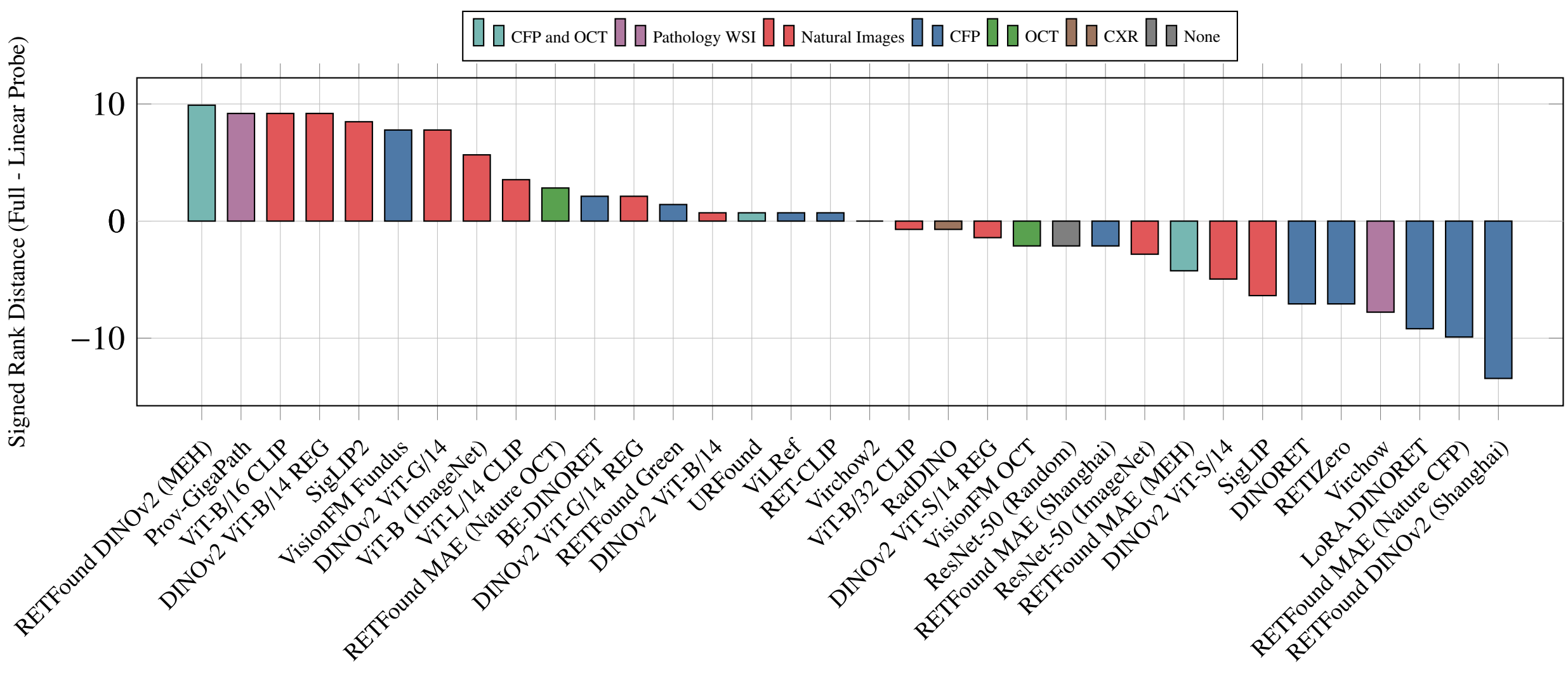

Figure A.6: Signed rank distance, showing the change in each encoder's mean rank between full fine-tuning and linear probing. A positive value indicates a better relative rank under linear probing. The distance is calculated as $(\text{rank}_{\text{Full}} - \text{rank}_{\text{LP}})/\sqrt{2}$. This metric geometrically corresponds to the signed perpendicular distance of each model from the $y = x$ diagonal in a scatter plot of the two rank types, thus providing a quantitative measure of rank stability. The bars are colored by the model's pretraining modalities. Models are listed from left to right by decreasing signed rank distances.

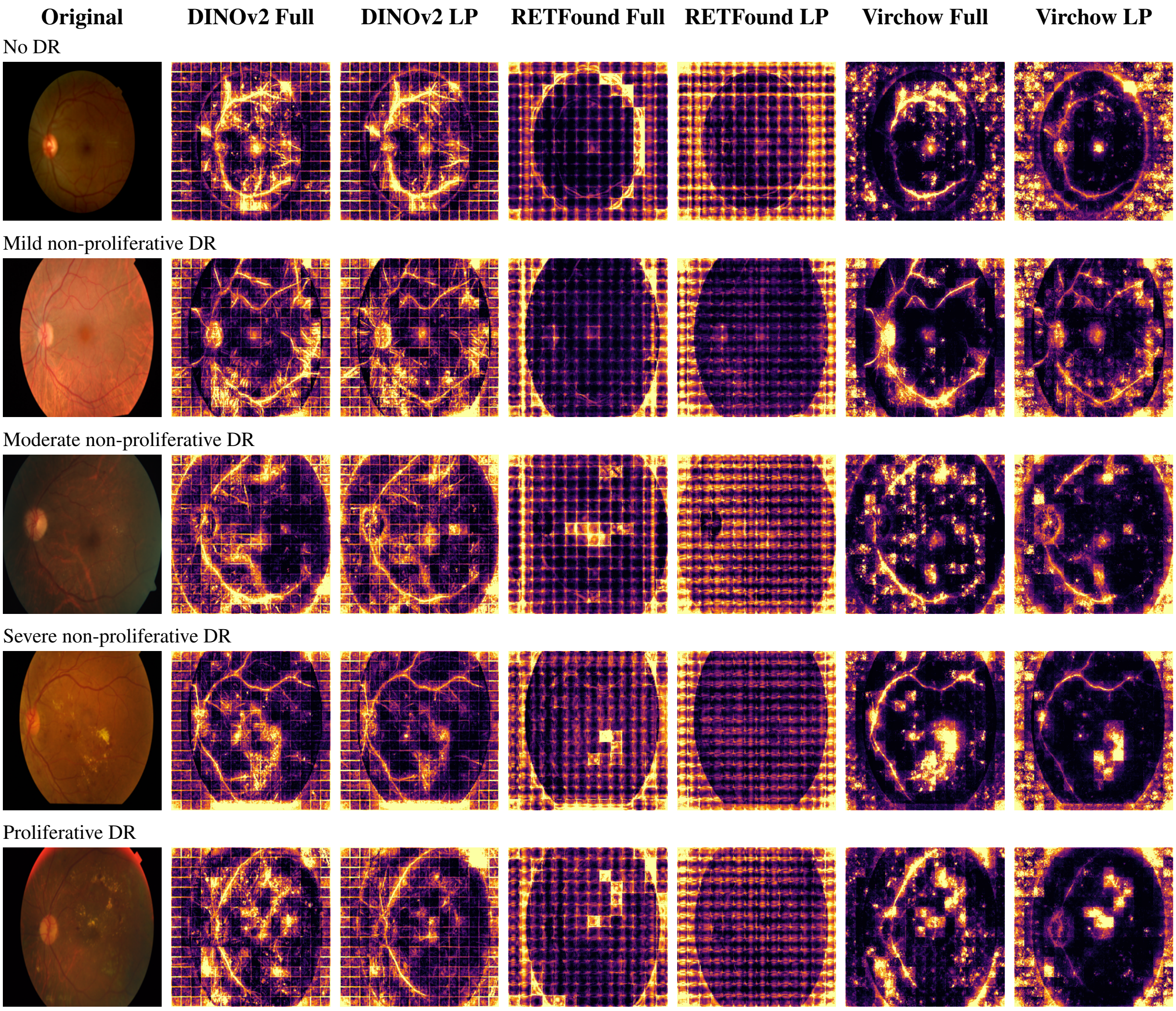

Figure A.7: NoiseTunnel saliency maps for diabetic retinopathy severity grading on APTOS. Representative images from the APTOS dataset are shown for each ICDR class. Columns show the original image followed by NoiseTunnel saliency maps for *DINOv2 ViT-B*, *RETFound*, and *Virchow* under full fine-tuning and linear probing.

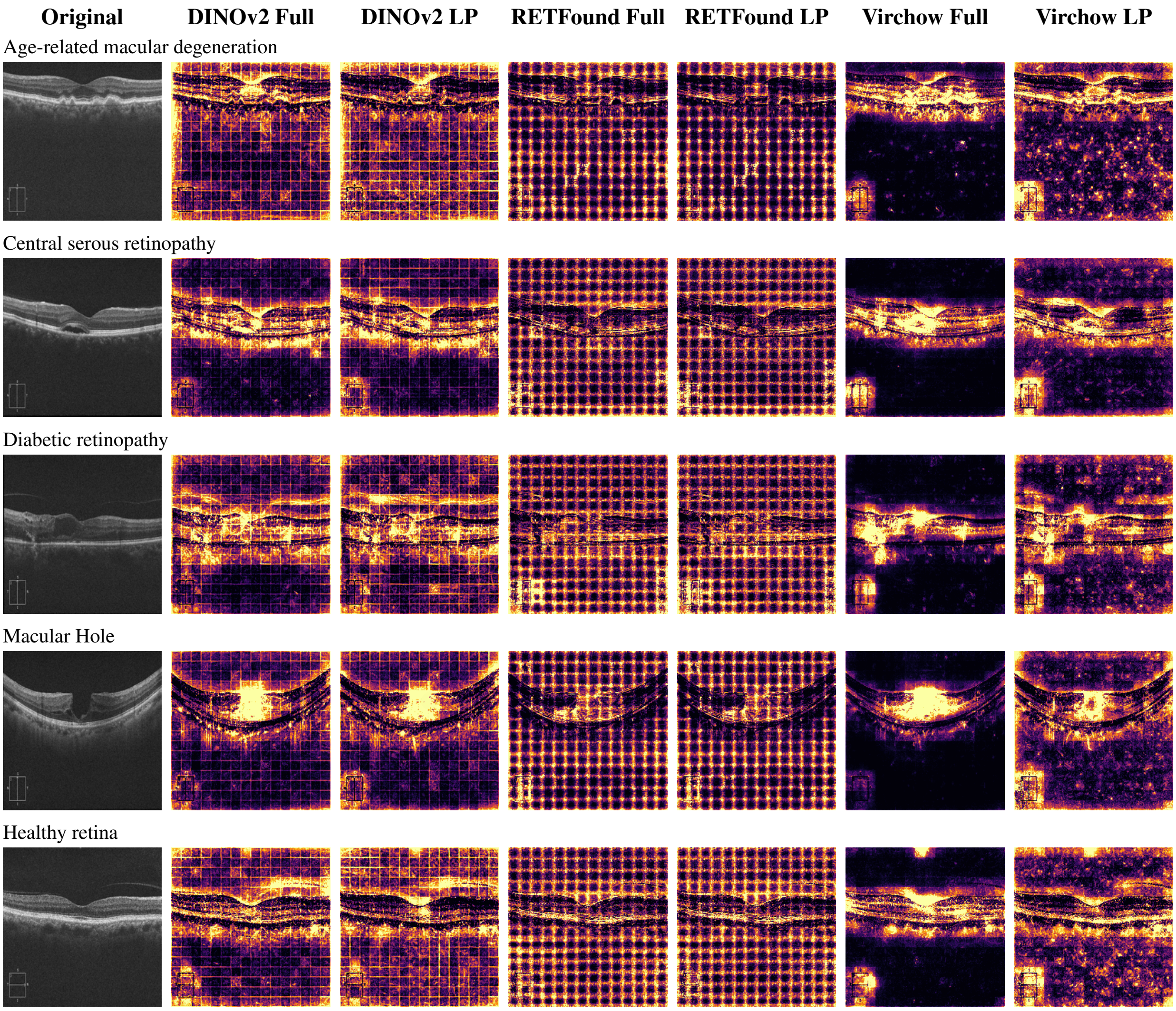

Figure A.8: NoiseTunnel saliency maps for OCT disease classification on OCTID. Representative images from the OCTID dataset are shown for each disease class. Columns show the original OCT image followed by NoiseTunnel saliency maps for *DINOv2 ViT-B*, *RETFound*, and *Virchow* under full fine-tuning and linear probing.

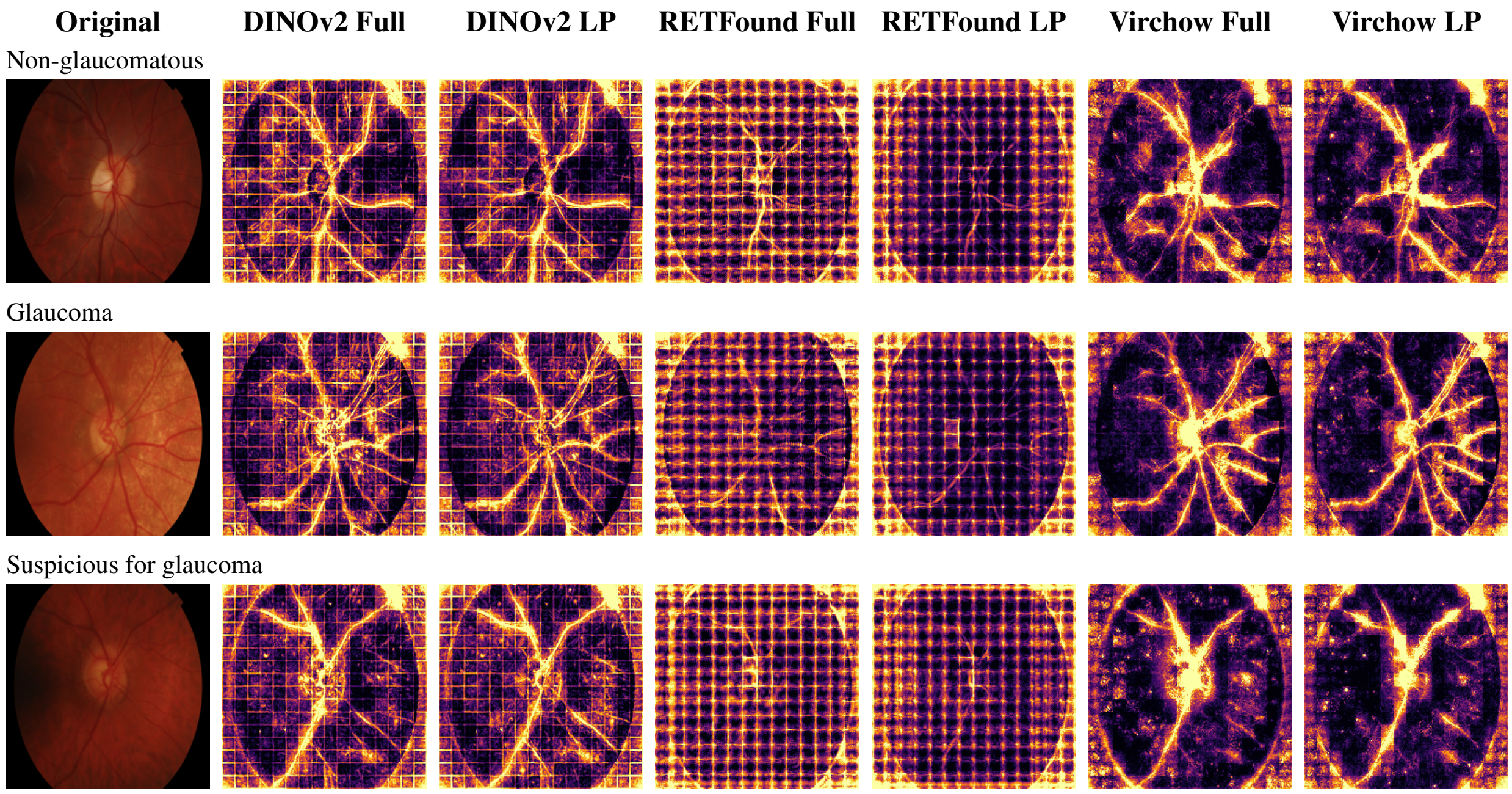

Figure A.9: NoiseTunnel saliency maps for glaucoma classification on PAPILA. Representative fundus images from the PAPILA dataset are shown for the normal, glaucoma, and suspicious classes. Columns show the original image followed by NoiseTunnel saliency maps for *DINOv2 ViT-B*, *RETFound*, and *Virchow* under full fine-tuning and linear probing.

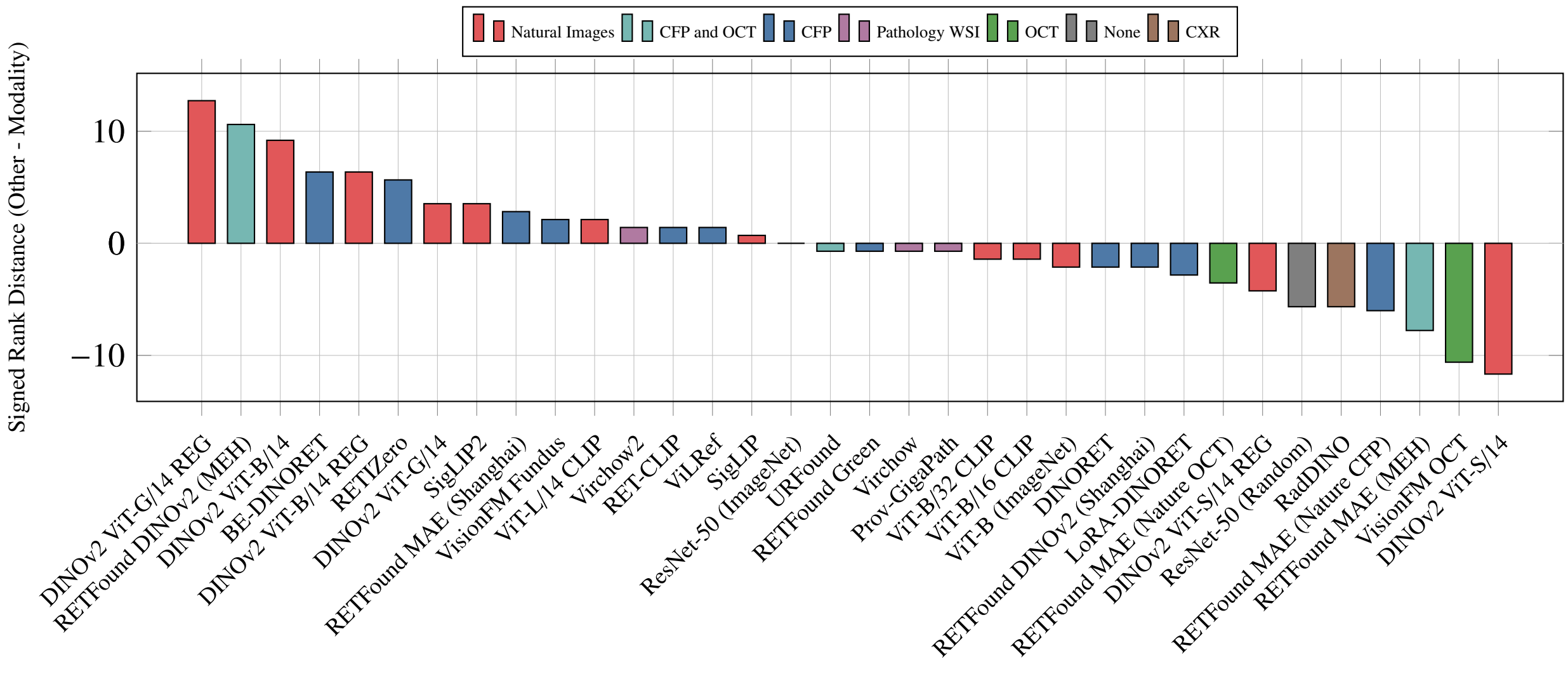

(a) Color Fundus Photograph classification tasks

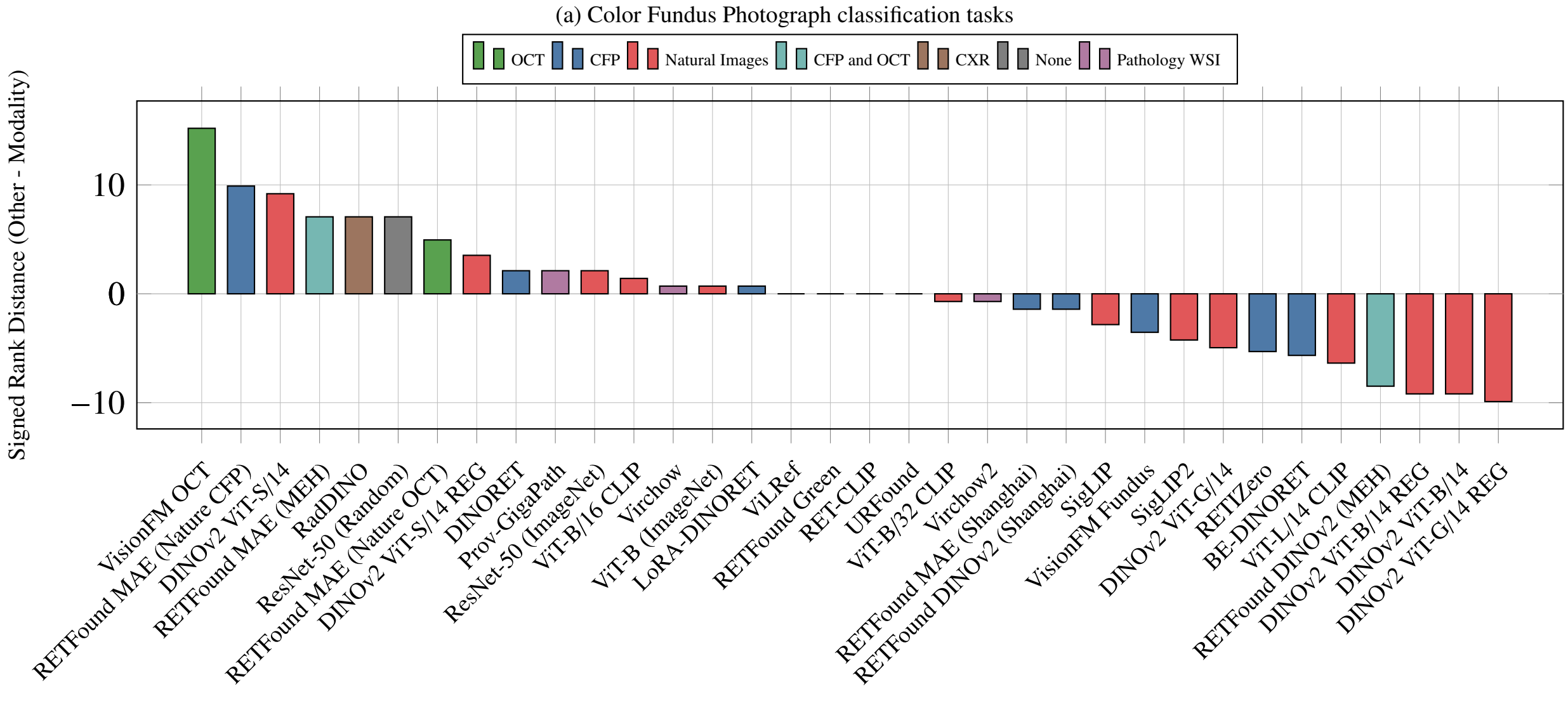

(b) Optical Coherence Tomography classification tasks

Figure A.10: Signed rank distance, showing the change in each encoder's mean rank between classification tasks of a given modality and tasks in other modalities. A positive value indicates a better relative rank for the given modality. The bars are colored by the model's pretraining modalities. Models are listed from left to right by decreasing signed rank distances.

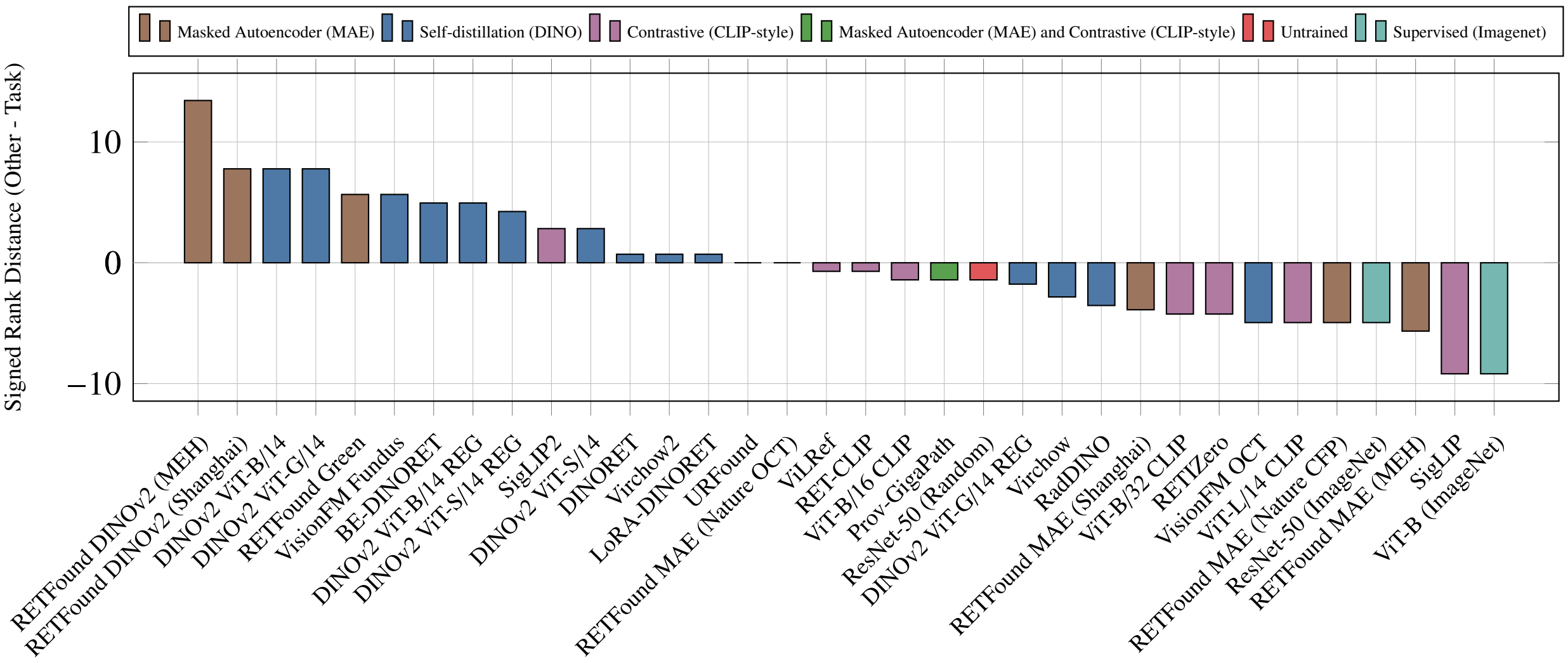

(a) Diabetic retinopathy staging tasks

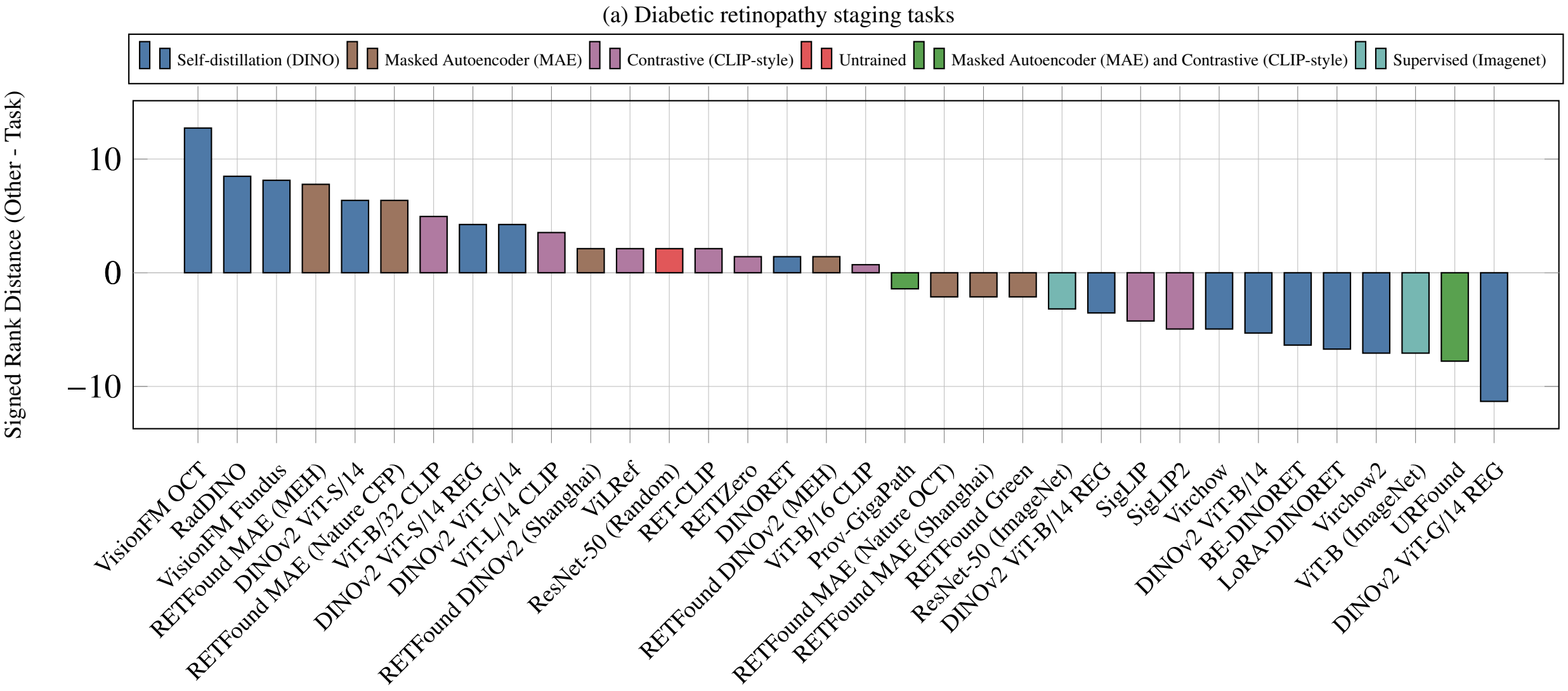

(b) Glaucoma classification tasks

Figure A.11: Signed rank distance, showing the change in each encoder's mean rank between classification tasks of a given type and other tasks. A positive value indicates a better relative rank for the given task type. The bars are colored by the model's pretraining supervision method. Models are listed from left to right by decreasing signed rank distances.

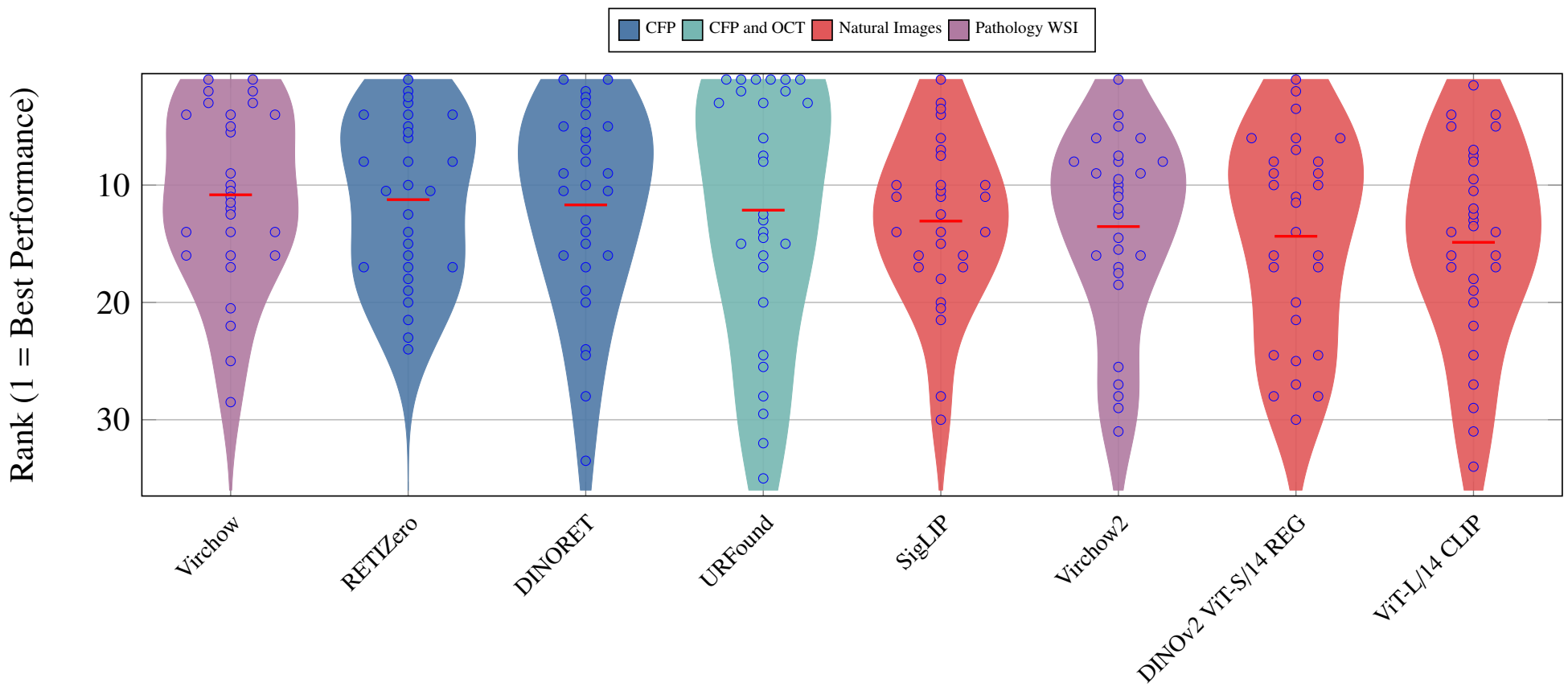

(a) All classification tasks

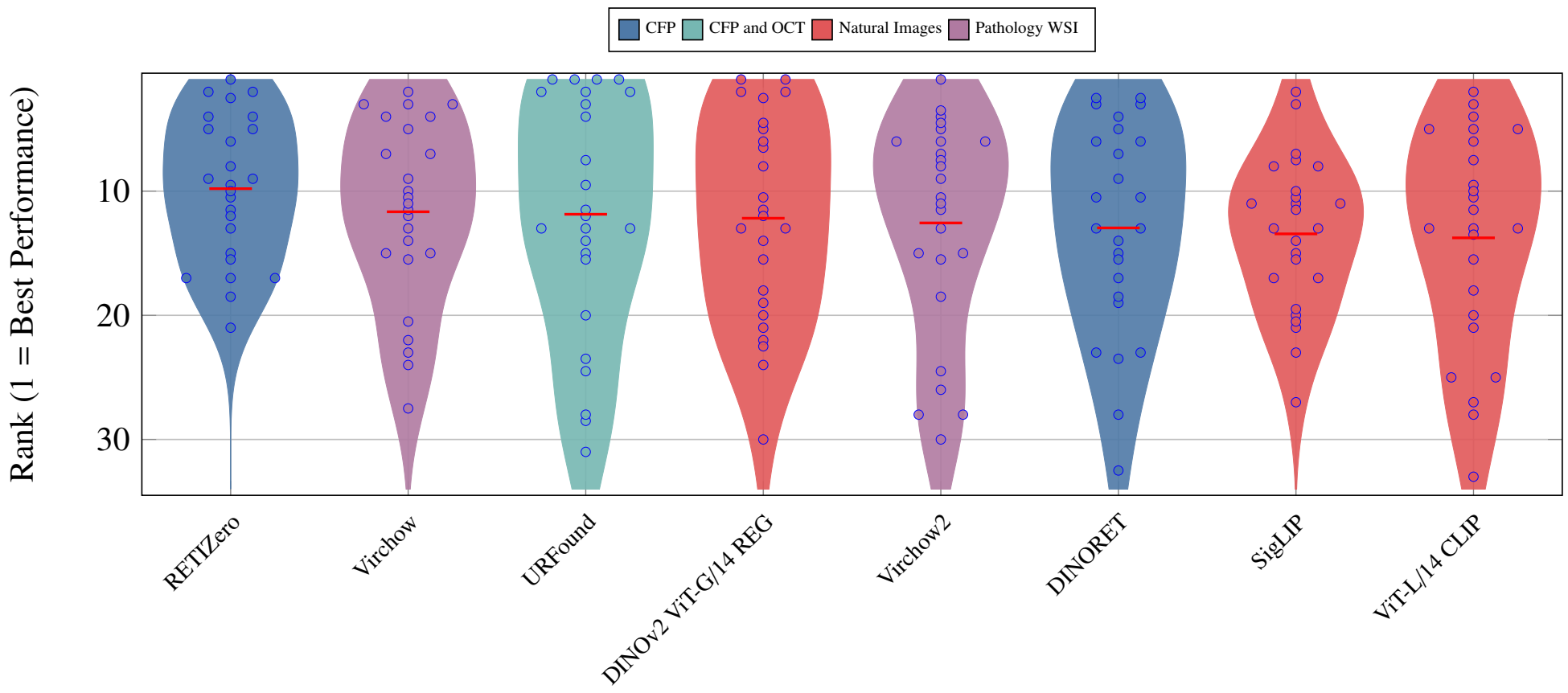

(b) Color Fundus Photograph classification tasks

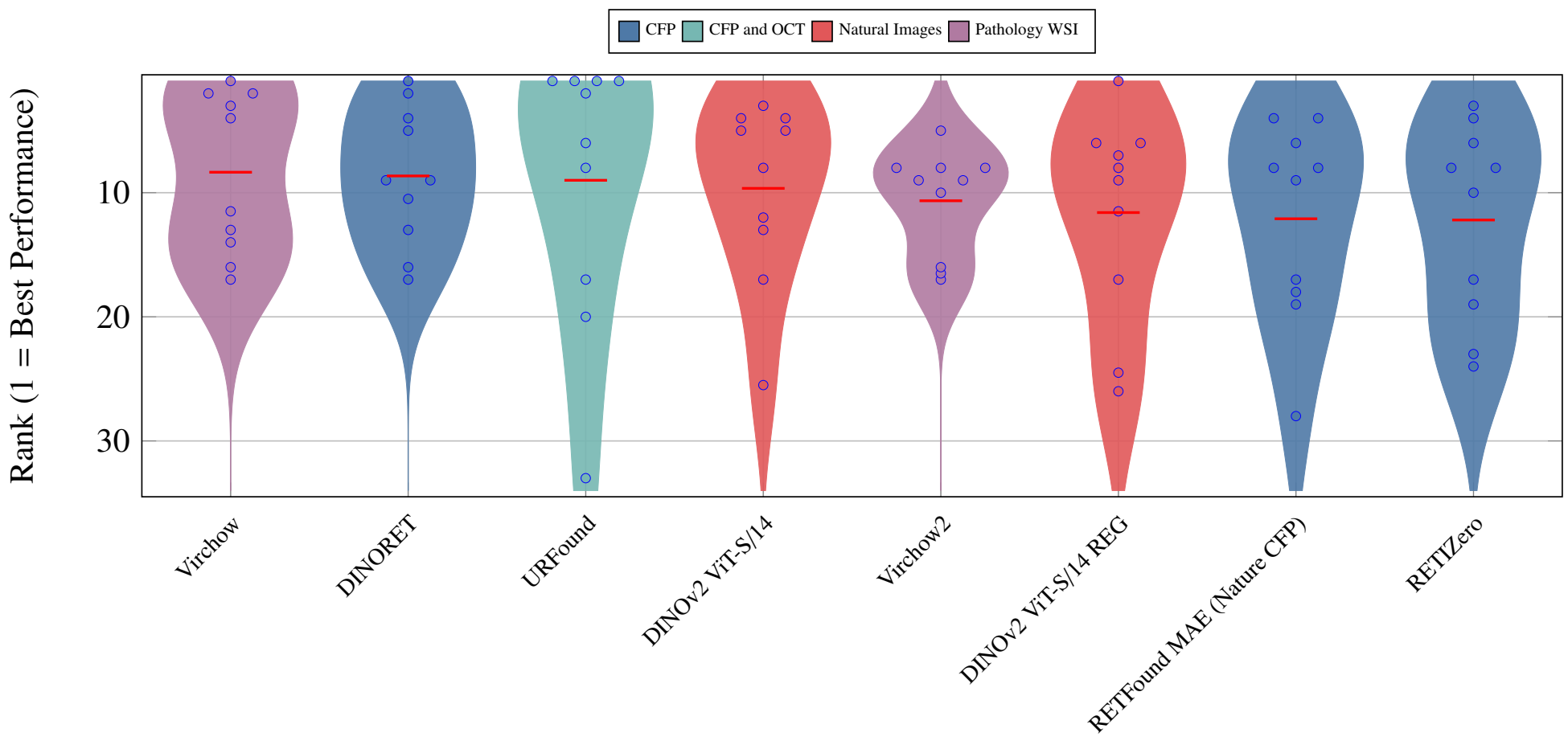

(c) Optical Coherence Tomography classification tasks

Figure A.12: Top eight encoders across all tasks, CFP tasks, and OCT tasks under full fine-tuning. Rank distributions are shown for the eight highest-ranked encoders across all classification tasks, Color Fundus Photograph classification tasks only, and Optical Coherence Tomography classification tasks only. Lower ranks indicate better performance. Each point represents the rank of an encoder on an individual task, based on the average test accuracy across three random seeds. Violin plots summarize the distribution of ranks for each encoder, and red horizontal lines indicate the mean rank. Colors indicate the pretraining domain of each encoder. Models are ordered from left to right by increasing mean rank within each subset.

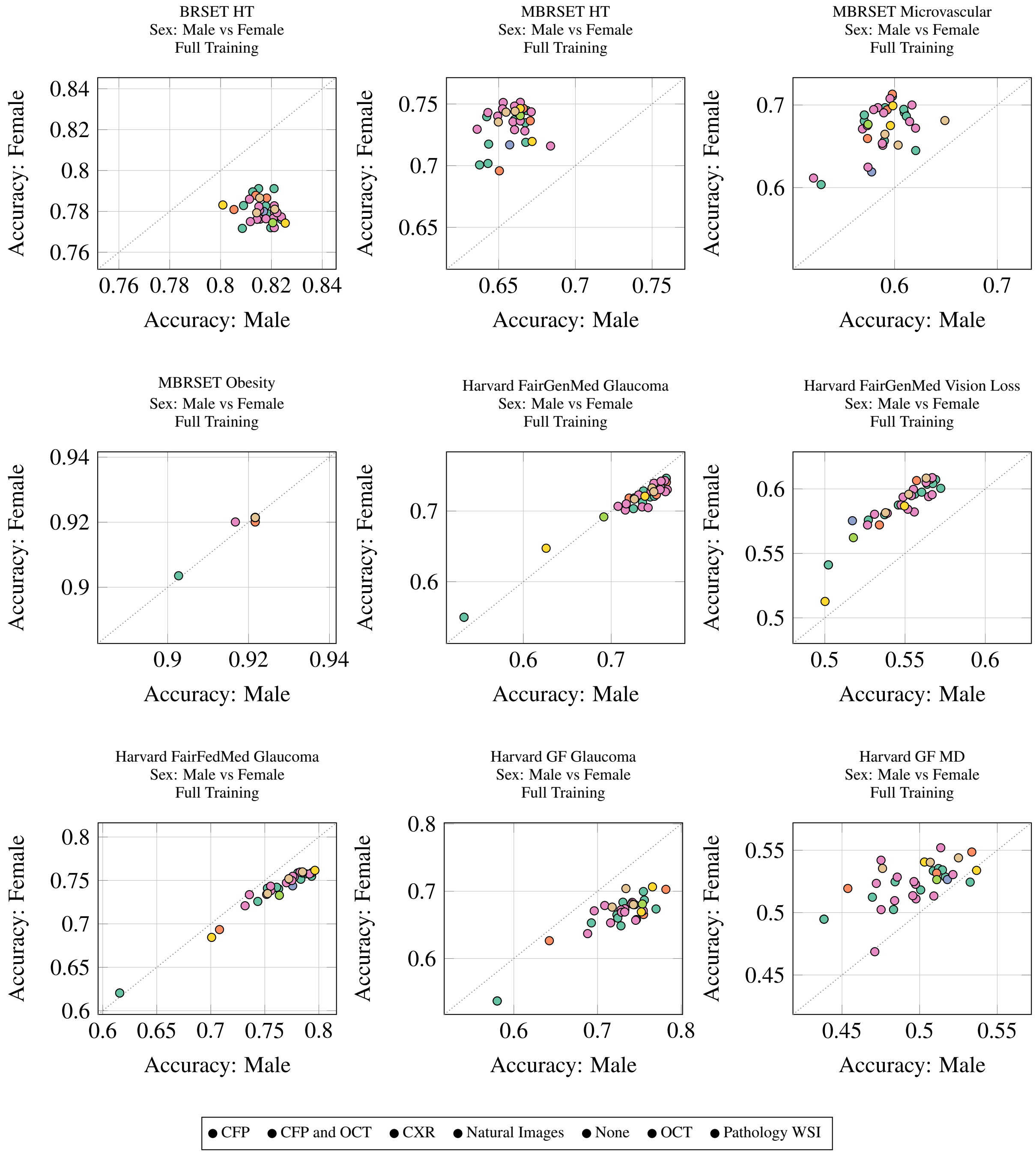

Figure A.13: Model accuracy on three datasets stratified by sex. Each point represents an encoder's performance on the female subgroup (y-axis) versus the male subgroup (x-axis).

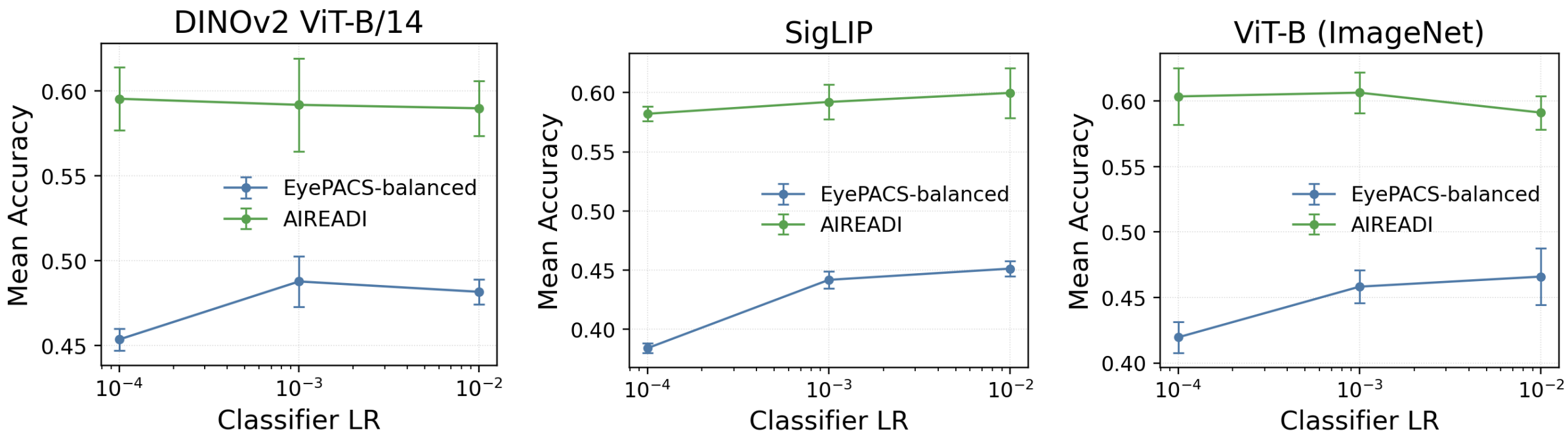

(a) DINOv2 ViT-B/14 (b) SigLIP (c) ViT-B (ImageNet)

EyePACS-balanced AIREADI

Figure A.14: Grid-search accuracy across classifier learning rates for the overlapping transfer-study encoders.

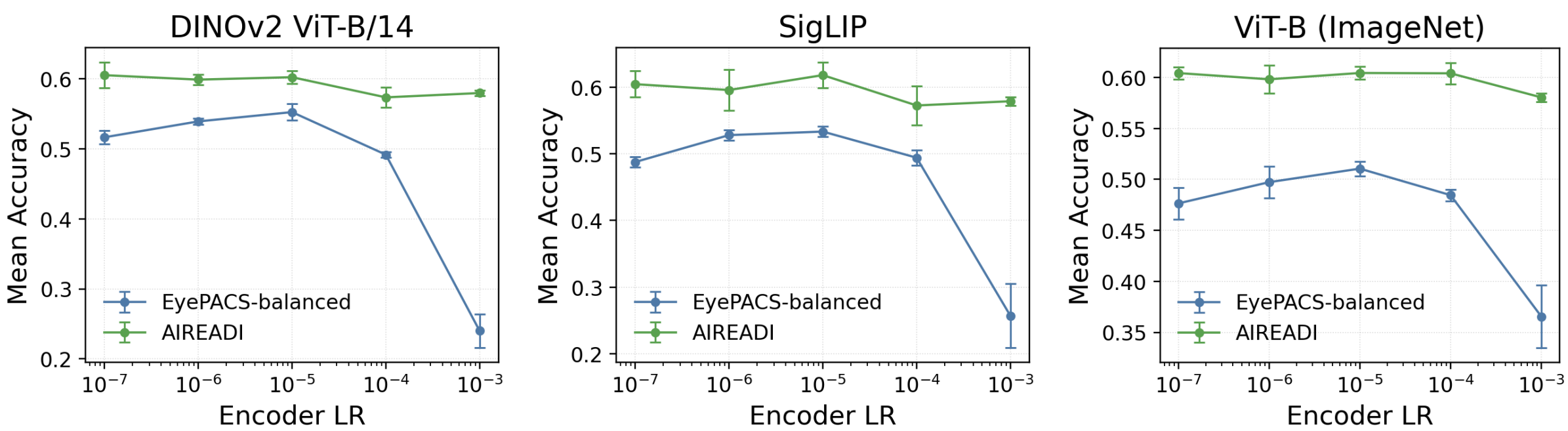

(a) DINOv2 ViT-B/14 (b) SigLIP (c) ViT-B (ImageNet)

EyePACS-balanced AIREADI

Figure A.15: Grid-search accuracy across encoder learning rates for the overlapping transfer-study encoders.

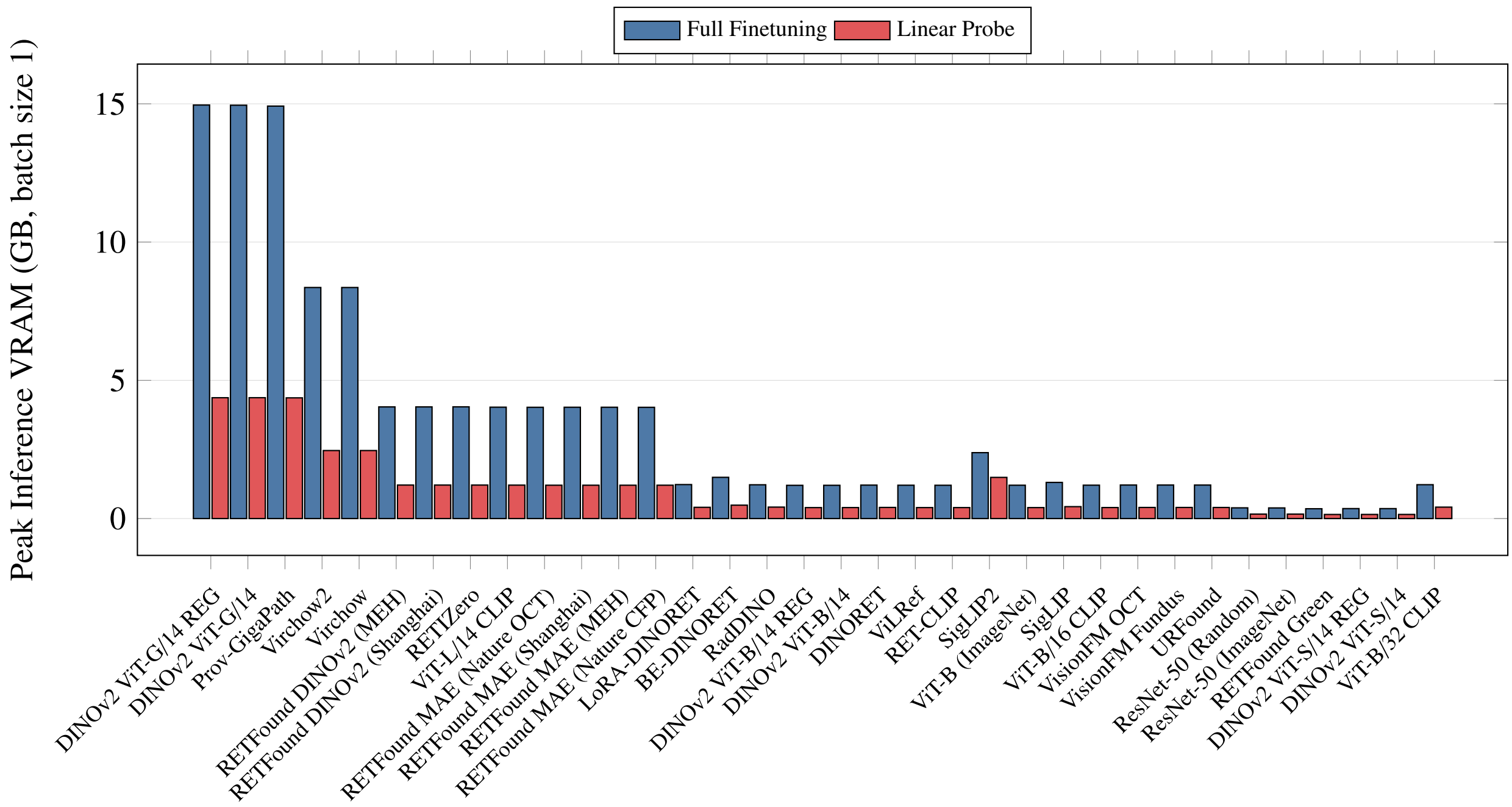

Figure A.16: Peak allocated GPU memory during inference at batch size 1 for each encoder model on the *APTOS* test split, shown for both full fine-tuning and linear probing. Values were recorded after warm-up and correspond to the maximum allocated VRAM observed during the measured inference batches.

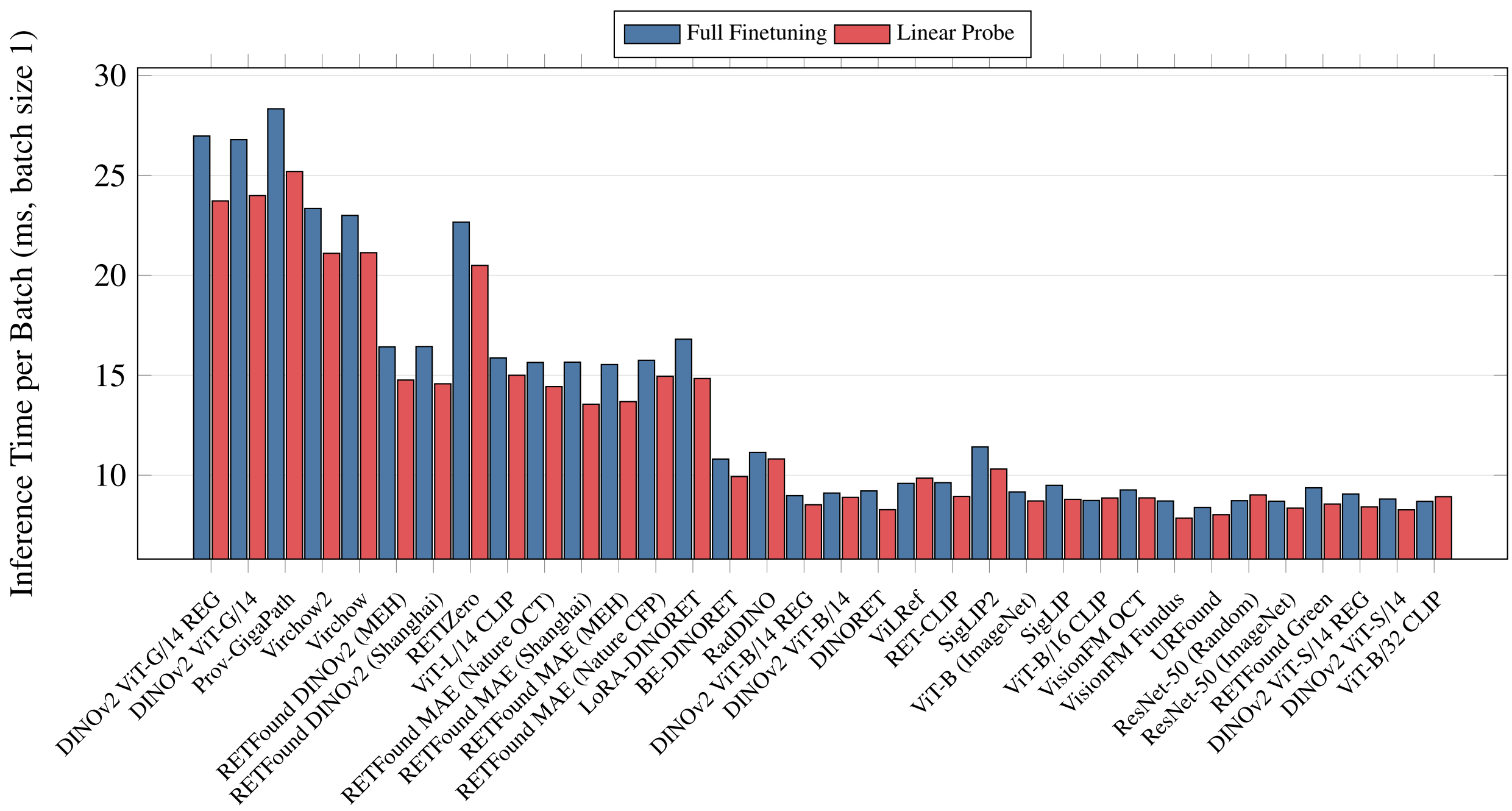

Figure A.17: Mean inference time per batch at batch size 1 for each encoder model on the *APTOS* test split, shown for both full fine-tuning and linear probing. Timings were measured after warm-up; at batch size 1, these values correspond to per-image inference latency.

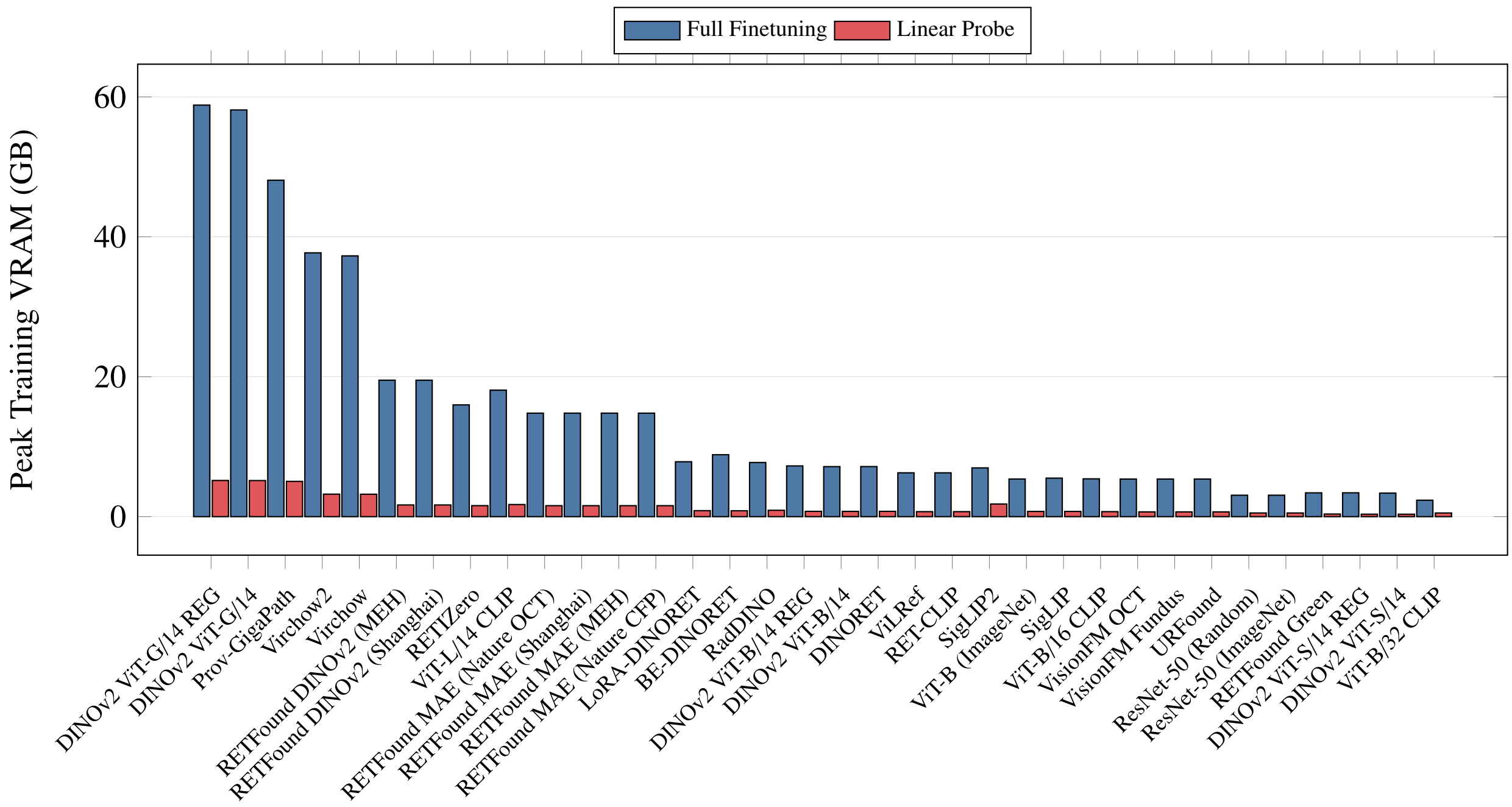

Figure A.18: Peak allocated GPU memory during training for each encoder model on the *APTOS* dataset with a batch size of 64, shown for both full fine-tuning and linear probing. Reported values correspond to the maximum allocated VRAM observed across the benchmarked training epochs.

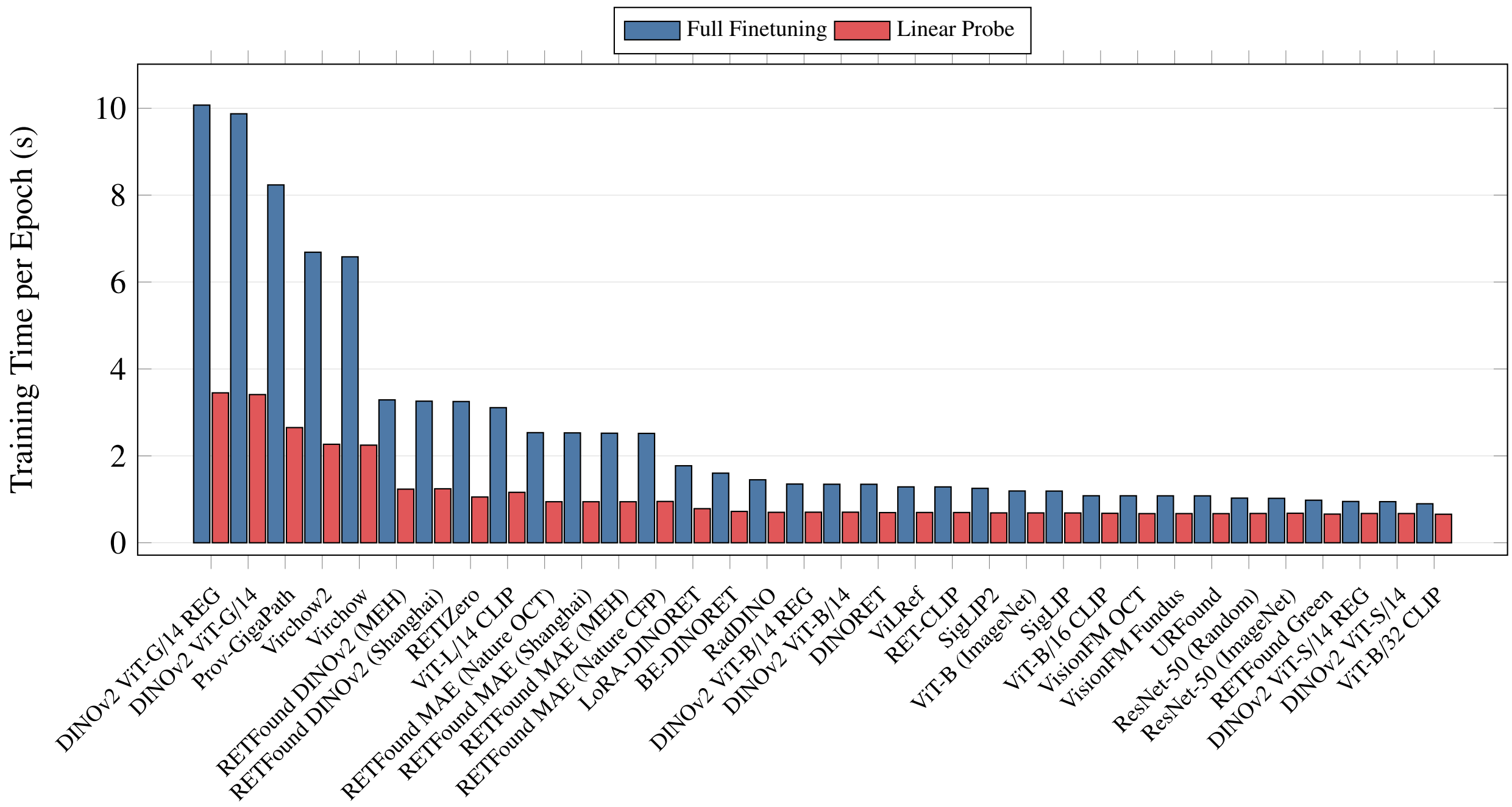

Figure A.19: Mean training time per epoch for each encoder model on the *APTOS* dataset with a batch size of 64 and 25 steps per epoch, shown for both full fine-tuning and linear probing. To reduce initialization effects, the average excludes the initial warm-up epochs of each benchmark run.

Appendix A.5. Raw Results

Appendix A.5.1. Balanced Training - Unbalanced Test

**Model**	**AIREADI Grouped Study Group CFP**	**AIREADI Grouped Study Group OCT**	**AIREADI Study Group CFP**	**AIREADI Study Group OCT**
BE-DINORET	$0.573 \pm 0.016_{(3)}$	$0.545 \pm 0.018_{(3)}$	$0.314 \pm 0.006_{(3)}$	$0.289 \pm 0.012_{(3)}$
DINORET	$0.562 \pm 0.024_{(3)}$	$0.589 \pm 0.031_{(3)}$	$0.323 \pm 0.015_{(3)}$	$0.292 \pm 0.042_{(3)}$
DINOv2 ViT-B/14	$0.571 \pm 0.006_{(3)}$	$0.528 \pm 0.015_{(3)}$	$0.321 \pm 0.030_{(3)}$	$0.288 \pm 0.013_{(3)}$
DINOv2 ViT-B/14 REG	$0.584 \pm 0.012_{(3)}$	$0.549 \pm 0.005_{(3)}$	$0.308 \pm 0.010_{(3)}$	$0.300 \pm 0.030_{(3)}$
DINOv2 ViT-G/14	$0.555 \pm 0.009_{(3)}$	$0.570 \pm 0.016_{(3)}$	$0.277 \pm 0.040_{(3)}$	$0.305 \pm 0.028_{(3)}$
DINOv2 ViT-G/14 REG	$0.583 \pm 0.007_{(3)}$	$0.522 \pm 0.025_{(3)}$	$0.308 \pm 0.057_{(3)}$	$0.298 \pm 0.029_{(3)}$
DINOv2 ViT-S/14	$0.586 \pm 0.010_{(3)}$	$0.575 \pm 0.026_{(3)}$	$0.307 \pm 0.037_{(3)}$	$0.293 \pm 0.021_{(3)}$
DINOv2 ViT-S/14 REG	$0.562 \pm 0.012_{(3)}$	$0.544 \pm 0.022_{(3)}$	$0.321 \pm 0.041_{(3)}$	$0.272 \pm 0.023_{(3)}$
LoRA-DINORET	$0.590 \pm 0.017_{(3)}$	$0.570 \pm 0.034_{(3)}$	$0.293 \pm 0.004_{(3)}$	$0.259 \pm 0.005_{(3)}$
Prov-GigaPath	$0.570 \pm 0.004_{(3)}$	$0.547 \pm 0.022_{(3)}$	$0.305 \pm 0.038_{(3)}$	$0.300 \pm 0.016_{(3)}$
RadDINO	$0.586 \pm 0.024_{(3)}$	$0.553 \pm 0.024_{(3)}$	$0.322 \pm 0.033_{(3)}$	$0.298 \pm 0.041_{(3)}$
ResNet-50 (ImageNet)	$0.572 \pm 0.010_{(3)}$	$0.534 \pm 0.032_{(3)}$	$0.315 \pm 0.057_{(3)}$	$0.303 \pm 0.006_{(3)}$
ResNet-50 (Random)	$0.537 \pm 0.024_{(3)}$	$0.517 \pm 0.037_{(3)}$	$0.269 \pm 0.014_{(3)}$	$0.267 \pm 0.026_{(3)}$
RET-CLIP	$0.516 \pm 0.030_{(3)}$	$0.468 \pm 0.034_{(3)}$	$0.249 \pm 0.015_{(3)}$	$0.287 \pm 0.033_{(3)}$
RETFound DINOv2 (MEH)	$0.570 \pm 0.015_{(3)}$	$0.545 \pm 0.033_{(3)}$	$0.323 \pm 0.027_{(3)}$	$0.282 \pm 0.016_{(3)}$
RETFound DINOv2 (Shanghai)	$0.568 \pm 0.013_{(3)}$	$0.547 \pm 0.014_{(3)}$	$0.303 \pm 0.026_{(3)}$	$0.284 \pm 0.022_{(3)}$
RETFound MAE (MEH)	$0.581 \pm 0.009_{(3)}$	$0.530 \pm 0.010_{(3)}$	$0.322 \pm 0.050_{(3)}$	$0.307 \pm 0.056_{(3)}$
RETFound MAE (Nature CFP)	$0.590 \pm 0.031_{(3)}$	$0.557 \pm 0.034_{(3)}$	$0.303 \pm 0.020_{(3)}$	$0.261 \pm 0.053_{(3)}$
RETFound MAE (Nature OCT)	$0.546 \pm 0.011_{(3)}$	$0.556 \pm 0.029_{(3)}$	$0.272 \pm 0.023_{(3)}$	$0.308 \pm 0.037_{(3)}$
RETFound MAE (Shanghai)	$0.589 \pm 0.014_{(3)}$	$0.548 \pm 0.008_{(3)}$	$0.307 \pm 0.023_{(3)}$	$0.303 \pm 0.012_{(3)}$
RETFound Green	$0.554 \pm 0.013_{(3)}$	$0.516 \pm 0.038_{(3)}$	$0.279 \pm 0.056_{(3)}$	$0.279 \pm 0.010_{(3)}$
RETIZero	$0.585 \pm 0.019_{(3)}$	$0.545 \pm 0.025_{(3)}$	$0.334 \pm 0.007_{(3)}$	$0.288 \pm 0.019_{(3)}$
SigLIP	$0.558 \pm 0.021_{(3)}$	$0.566 \pm 0.010_{(3)}$	$0.281 \pm 0.051_{(3)}$	$0.284 \pm 0.042_{(3)}$
SigLIP2	$0.560 \pm 0.008_{(3)}$	$0.465 \pm 0.021_{(3)}$	$0.330 \pm 0.019_{(3)}$	$0.277 \pm 0.034_{(3)}$
URFound	$0.590 \pm 0.011_{(3)}$	$0.576 \pm 0.015_{(3)}$	$0.328 \pm 0.014_{(3)}$	$0.301 \pm 0.039_{(3)}$
ViLRef	$0.516 \pm 0.030_{(3)}$	$0.468 \pm 0.034_{(3)}$	$0.249 \pm 0.015_{(3)}$	$0.287 \pm 0.033_{(3)}$
Virchow	$0.579 \pm 0.019_{(3)}$	$0.571 \pm 0.025_{(3)}$	$0.333 \pm 0.026_{(3)}$	$0.311 \pm 0.023_{(3)}$
Virchow2	$0.559 \pm 0.015_{(3)}$	$0.544 \pm 0.037_{(3)}$	$0.292 \pm 0.035_{(3)}$	$0.312 \pm 0.006_{(3)}$
VisionFM Fundus	$0.585 \pm 0.010_{(3)}$	$0.543 \pm 0.026_{(3)}$	$0.292 \pm 0.024_{(3)}$	$0.276 \pm 0.013_{(3)}$
VisionFM OCT	$0.574 \pm 0.018_{(3)}$	$0.538 \pm 0.037_{(3)}$	$0.323 \pm 0.017_{(3)}$	$0.256 \pm 0.019_{(3)}$
ViT-B/16 CLIP	$0.568 \pm 0.017_{(3)}$	$0.527 \pm 0.032_{(3)}$	$0.315 \pm 0.036_{(3)}$	$0.293 \pm 0.012_{(3)}$
ViT-B/32 CLIP	$0.587 \pm 0.019_{(3)}$	$0.539 \pm 0.017_{(3)}$	$0.312 \pm 0.018_{(3)}$	$0.273 \pm 0.012_{(3)}$
ViT-L/14 CLIP	$0.580 \pm 0.023_{(3)}$	$0.580 \pm 0.016_{(3)}$	$0.294 \pm 0.015_{(3)}$	$0.308 \pm 0.048_{(3)}$
ViT-B (ImageNet)	$0.572 \pm 0.030_{(3)}$	$0.572 \pm 0.010_{(3)}$	$0.321 \pm 0.023_{(3)}$	$0.280 \pm 0.011_{(3)}$

Table A.2: Performance comparison across datasets using full training. Values show test accuracy as mean ± standard deviation. – indicates no data available. Subscript numbers in parentheses indicate sample size.

Model	APTOS	BRSET DR	BRSET HT	DR-TID
BE-DINORET	$0.762 \pm 0.020_{(3)}$	$0.672 \pm 0.044_{(3)}$	$0.699 \pm 0.013_{(3)}$	$0.432 \pm 0.042_{(3)}$
DINORET	$0.733 \pm 0.028_{(3)}$	$0.485 \pm 0.305_{(3)}$	$0.705 \pm 0.013_{(3)}$	$0.438 \pm 0.026_{(3)}$
DINOv2 ViT-B/14	$0.773 \pm 0.030_{(3)}$	$0.660 \pm 0.098_{(3)}$	$0.707 \pm 0.008_{(3)}$	$0.459 \pm 0.024_{(3)}$
DINOv2 ViT-B/14 REG	$0.756 \pm 0.019_{(3)}$	$0.666 \pm 0.024_{(3)}$	$0.708 \pm 0.008_{(3)}$	$0.462 \pm 0.057_{(3)}$
DINOv2 ViT-G/14	$0.743 \pm 0.012_{(3)}$	$0.722 \pm 0.113_{(3)}$	$0.730 \pm 0.010_{(3)}$	$0.426 \pm 0.077_{(3)}$
DINOv2 ViT-G/14 REG	$0.762 \pm 0.007_{(3)}$	$0.716 \pm 0.071_{(3)}$	$0.704 \pm 0.001_{(3)}$	$0.401 \pm 0.071_{(3)}$
DINOv2 ViT-S/14	$0.730 \pm 0.010_{(3)}$	$0.672 \pm 0.033_{(3)}$	$0.702 \pm 0.023_{(3)}$	$0.448 \pm 0.091_{(3)}$
DINOv2 ViT-S/14 REG	$0.755 \pm 0.019_{(3)}$	$0.657 \pm 0.153_{(3)}$	$0.700 \pm 0.006_{(3)}$	$0.379 \pm 0.073_{(3)}$
LoRA-DINORET	$0.767 \pm 0.015_{(3)}$	$0.589 \pm 0.227_{(3)}$	$0.715 \pm 0.020_{(3)}$	$0.498 \pm 0.013_{(3)}$
Prov-GigaPath	$0.755 \pm 0.031_{(3)}$	$0.758 \pm 0.016_{(3)}$	$0.701 \pm 0.011_{(3)}$	$0.416 \pm 0.056_{(3)}$
RadDINO	$0.722 \pm 0.021_{(3)}$	$0.585 \pm 0.052_{(3)}$	$0.694 \pm 0.012_{(3)}$	$0.346 \pm 0.030_{(3)}$
ResNet-50 (ImageNet)	$0.736 \pm 0.024_{(3)}$	$0.644 \pm 0.013_{(3)}$	$0.709 \pm 0.006_{(3)}$	$0.332 \pm 0.022_{(3)}$
ResNet-50 (Random)	$0.636 \pm 0.060_{(3)}$	$0.321 \pm 0.288_{(3)}$	$0.622 \pm 0.015_{(3)}$	$0.190 \pm 0.039_{(3)}$
RET-CLIP	$0.378 \pm 0.038_{(3)}$	$0.259 \pm 0.220_{(3)}$	$0.652 \pm 0.005_{(3)}$	$0.193 \pm 0.061_{(3)}$
RETFound DINOv2 (MEH)	$0.775 \pm 0.019_{(3)}$	$0.688 \pm 0.005_{(3)}$	$0.716 \pm 0.013_{(3)}$	$0.393 \pm 0.022_{(3)}$
RETFound DINOv2 (Shanghai)	$0.748 \pm 0.008_{(3)}$	$0.745 \pm 0.042_{(3)}$	$0.735 \pm 0.015_{(3)}$	$0.348 \pm 0.013_{(3)}$
RETFound MAE (MEH)	$0.757 \pm 0.035_{(3)}$	$0.613 \pm 0.132_{(3)}$	$0.685 \pm 0.010_{(3)}$	$0.398 \pm 0.063_{(3)}$
RETFound MAE (Nature CFP)	$0.765 \pm 0.067_{(3)}$	$0.642 \pm 0.113_{(3)}$	$0.696 \pm 0.034_{(3)}$	$0.406 \pm 0.041_{(3)}$
RETFound MAE (Nature OCT)	$0.614 \pm 0.026_{(3)}$	$0.280 \pm 0.029_{(3)}$	$0.657 \pm 0.008_{(3)}$	$0.199 \pm 0.029_{(3)}$
RETFound MAE (Shanghai)	$0.776 \pm 0.014_{(3)}$	$0.754 \pm 0.163_{(3)}$	$0.713 \pm 0.015_{(3)}$	$0.489 \pm 0.073_{(3)}$
RETFound Green	$0.719 \pm 0.037_{(3)}$	$0.627 \pm 0.188_{(3)}$	$0.712 \pm 0.009_{(3)}$	$0.418 \pm 0.027_{(3)}$
RETIZero	$0.804 \pm 0.029_{(3)}$	$0.746 \pm 0.096_{(3)}$	$0.710 \pm 0.003_{(3)}$	$0.522 \pm 0.039_{(3)}$
SigLIP	$0.780 \pm 0.028_{(3)}$	$0.539 \pm 0.166_{(3)}$	$0.702 \pm 0.018_{(3)}$	$0.402 \pm 0.064_{(3)}$
SigLIP2	$0.756 \pm 0.021_{(3)}$	$0.587 \pm 0.026_{(3)}$	$0.698 \pm 0.008_{(3)}$	$0.396 \pm 0.037_{(3)}$
URFound	$0.786 \pm 0.028_{(3)}$	$0.788 \pm 0.064_{(3)}$	$0.703 \pm 0.012_{(3)}$	$0.479 \pm 0.028_{(3)}$
ViLRef	$0.378 \pm 0.038_{(3)}$	$0.259 \pm 0.220_{(3)}$	$0.652 \pm 0.005_{(3)}$	$0.193 \pm 0.061_{(3)}$
Virchow	$0.755 \pm 0.013_{(3)}$	$0.616 \pm 0.200_{(3)}$	$0.669 \pm 0.015_{(3)}$	$0.376 \pm 0.062_{(3)}$
Virchow2	$0.774 \pm 0.021_{(3)}$	$0.815 \pm 0.022_{(3)}$	$0.704 \pm 0.018_{(3)}$	$0.412 \pm 0.053_{(3)}$
VisionFM Fundus	$0.698 \pm 0.009_{(3)}$	$0.619 \pm 0.044_{(3)}$	$0.712 \pm 0.010_{(3)}$	$0.355 \pm 0.038_{(3)}$
VisionFM OCT	$0.646 \pm 0.039_{(3)}$	$0.433 \pm 0.114_{(3)}$	$0.673 \pm 0.010_{(3)}$	$0.323 \pm 0.052_{(3)}$
ViT-B/16 CLIP	$0.764 \pm 0.028_{(3)}$	$0.604 \pm 0.036_{(3)}$	$0.706 \pm 0.024_{(3)}$	$0.445 \pm 0.022_{(3)}$
ViT-B/32 CLIP	$0.744 \pm 0.010_{(3)}$	$0.578 \pm 0.118_{(3)}$	$0.704 \pm 0.009_{(3)}$	$0.416 \pm 0.007_{(3)}$
ViT-L/14 CLIP	$0.781 \pm 0.029_{(3)}$	$0.674 \pm 0.070_{(3)}$	$0.711 \pm 0.038_{(3)}$	$0.428 \pm 0.071_{(3)}$
ViT-B (ImageNet)	$0.753 \pm 0.012_{(3)}$	$0.605 \pm 0.104_{(3)}$	$0.695 \pm 0.035_{(3)}$	$0.389 \pm 0.084_{(3)}$

Table A.3: Performance comparison across datasets using full training. Values show test accuracy as mean ± standard deviation. – indicates no data available. Subscript numbers in parentheses indicate sample size.

Model	EyePACS	EyePACS Balanced	FIMD	GRAPE MD
BE-DINORET	$0.499 \pm 0.018_{(3)}$	$0.505 \pm 0.018_{(3)}$	$0.455 \pm 0.091_{(3)}$	$0.592 \pm 0.088_{(3)}$
DINORET	$0.519 \pm 0.150_{(3)}$	$0.552 \pm 0.011_{(3)}$	$0.576 \pm 0.262_{(3)}$	$0.720 \pm 0.092_{(3)}$
DINOv2 ViT-B/14	$0.497 \pm 0.018_{(3)}$	$0.500 \pm 0.008_{(3)}$	$0.455 \pm 0.157_{(3)}$	$0.688 \pm 0.063_{(3)}$
DINOv2 ViT-B/14 REG	$0.517 \pm 0.024_{(3)}$	$0.544 \pm 0.022_{(3)}$	$0.515 \pm 0.189_{(3)}$	$0.588 \pm 0.089_{(3)}$
DINOv2 ViT-G/14	$0.513 \pm 0.115_{(3)}$	$0.567 \pm 0.022_{(3)}$	$0.667 \pm 0.189_{(3)}$	$0.369 \pm 0.070_{(3)}$
DINOv2 ViT-G/14 REG	$0.506 \pm 0.028_{(3)}$	$0.510 \pm 0.021_{(3)}$	$0.515 \pm 0.052_{(3)}$	$0.396 \pm 0.222_{(3)}$
DINOv2 ViT-S/14	$0.536 \pm 0.095_{(3)}$	$0.543 \pm 0.010_{(3)}$	$0.667 \pm 0.139_{(3)}$	$0.532 \pm 0.074_{(3)}$
DINOv2 ViT-S/14 REG	$0.548 \pm 0.030_{(3)}$	$0.546 \pm 0.020_{(3)}$	$0.545_{(3)}$	$0.459 \pm 0.015_{(3)}$
LoRA-DINORET	$0.438 \pm 0.054_{(3)}$	$0.555 \pm 0.020_{(3)}$	$0.636 \pm 0.091_{(3)}$	$0.426 \pm 0.225_{(3)}$
Prov-GigaPath	$0.489 \pm 0.011_{(3)}$	$0.494 \pm 0.014_{(3)}$	$0.515 \pm 0.052_{(3)}$	$0.508 \pm 0.071_{(3)}$
RadDINO	$0.486 \pm 0.123_{(3)}$	$0.483 \pm 0.018_{(3)}$	$0.576 \pm 0.105_{(3)}$	$0.558 \pm 0.063_{(3)}$
ResNet-50 (ImageNet)	$0.464 \pm 0.033_{(3)}$	$0.494 \pm 0.013_{(3)}$	$0.606 \pm 0.189_{(3)}$	$0.614 \pm 0.035_{(3)}$
ResNet-50 (Random)	$0.248 \pm 0.045_{(3)}$	$0.266 \pm 0.005_{(3)}$	$0.515 \pm 0.052_{(3)}$	$0.562 \pm 0.294_{(3)}$
RET-CLIP	$0.244 \pm 0.023_{(3)}$	$0.220 \pm 0.016_{(3)}$	$0.606 \pm 0.105_{(3)}$	$0.406 \pm 0.228_{(3)}$
RETFound DINOv2 (MEH)	$0.531 \pm 0.009_{(3)}$	$0.540 \pm 0.008_{(3)}$	$0.606 \pm 0.189_{(3)}$	$0.530 \pm 0.148_{(3)}$
RETFound DINOv2 (Shanghai)	$0.502 \pm 0.042_{(3)}$	$0.553 \pm 0.007_{(3)}$	$0.667 \pm 0.189_{(3)}$	$0.484 \pm 0.138_{(3)}$
RETFound MAE (MEH)	$0.435 \pm 0.076_{(3)}$	$0.510 \pm 0.023_{(3)}$	$0.545 \pm 0.157_{(3)}$	$0.525 \pm 0.225_{(3)}$
RETFound MAE (Nature CFP)	$0.633 \pm 0.044_{(3)}$	$0.492 \pm 0.001_{(3)}$	$0.545 \pm 0.091_{(3)}$	$0.331 \pm 0.264_{(3)}$
RETFound MAE (Nature OCT)	$0.254 \pm 0.032_{(3)}$	$0.261 \pm 0.008_{(3)}$	$0.515 \pm 0.229_{(3)}$	$0.564 \pm 0.109_{(3)}$
RETFound MAE (Shanghai)	$0.560 \pm 0.089_{(3)}$	$0.520 \pm 0.007_{(3)}$	$0.485 \pm 0.105_{(3)}$	$0.598 \pm 0.200_{(3)}$
RETFound Green	$0.518 \pm 0.038_{(3)}$	$0.523 \pm 0.018_{(3)}$	$0.576 \pm 0.189_{(3)}$	$0.606 \pm 0.049_{(3)}$
RETIZero	$0.556 \pm 0.021_{(3)}$	$0.542 \pm 0.001_{(3)}$	$0.576 \pm 0.139_{(3)}$	$0.365 \pm 0.213_{(3)}$
SigLIP	$0.399 \pm 0.128_{(3)}$	$0.532 \pm 0.008_{(3)}$	$0.576 \pm 0.139_{(3)}$	$0.548 \pm 0.131_{(3)}$
SigLIP2	$0.463 \pm 0.024_{(3)}$	$0.491 \pm 0.009_{(3)}$	$0.485 \pm 0.052_{(3)}$	$0.574 \pm 0.044_{(3)}$
URFound	$0.510 \pm 0.073_{(3)}$	$0.553 \pm 0.007_{(3)}$	$0.515 \pm 0.105_{(3)}$	$0.639 \pm 0.251_{(3)}$
ViLRef	$0.244 \pm 0.023_{(3)}$	$0.220 \pm 0.016_{(3)}$	$0.606 \pm 0.105_{(3)}$	$0.406 \pm 0.228_{(3)}$
Virchow	$0.542 \pm 0.150_{(3)}$	$0.557 \pm 0.005_{(3)}$	$0.576 \pm 0.139_{(3)}$	$0.698 \pm 0.047_{(3)}$
Virchow2	$0.552 \pm 0.029_{(3)}$	$0.541 \pm 0.007_{(3)}$	$0.485 \pm 0.052_{(3)}$	$0.459 \pm 0.250_{(3)}$
VisionFM Fundus	$0.496 \pm 0.017_{(3)}$	$0.512 \pm 0.021_{(3)}$	$0.545 \pm 0.091_{(3)}$	$0.447 \pm 0.217_{(3)}$
VisionFM OCT	$0.384 \pm 0.152_{(3)}$	$0.433 \pm 0.016_{(3)}$	$0.485 \pm 0.052_{(3)}$	$0.706 \pm 0.106_{(3)}$
ViT-B/16 CLIP	$0.500 \pm 0.038_{(3)}$	$0.541 \pm 0.017_{(3)}$	$0.485 \pm 0.105_{(3)}$	$0.512 \pm 0.292_{(3)}$
ViT-B/32 CLIP	$0.499 \pm 0.043_{(3)}$	$0.498 \pm 0.013_{(3)}$	$0.485 \pm 0.139_{(3)}$	$0.440 \pm 0.231_{(3)}$
ViT-L/14 CLIP	$0.620 \pm 0.075_{(3)}$	$0.543 \pm 0.024_{(3)}$	$0.576 \pm 0.105_{(3)}$	$0.598 \pm 0.204_{(3)}$
ViT-B (ImageNet)	$0.509 \pm 0.085_{(3)}$	$0.517 \pm 0.003_{(3)}$	$0.606 \pm 0.105_{(3)}$	$0.638 \pm 0.120_{(3)}$

Table A.4: Performance comparison across datasets using full training. Values show test accuracy as mean ± standard deviation. – indicates no data available. Subscript numbers in parentheses indicate sample size.

Model	GRAPE PLR2	GRAPE PLR3	Harvard FairFedMed Glaucoma	Harvard FairGenMed Glaucoma
BE-DINORET	$0.486 \pm 0.128_{(3)}$	$0.472 \pm 0.124_{(3)}$	$0.740 \pm 0.004_{(3)}$	$0.708 \pm 0.016_{(3)}$
DINORET	$0.588 \pm 0.156_{(3)}$	$0.537 \pm 0.063_{(3)}$	$0.764 \pm 0.002_{(3)}$	$0.744 \pm 0.004_{(3)}$
DINOv2 ViT-B/14	$0.491 \pm 0.206_{(3)}$	$0.504 \pm 0.025_{(3)}$	$0.729 \pm 0.005_{(3)}$	$0.708 \pm 0.002_{(3)}$
DINOv2 ViT-B/14 REG	$0.365 \pm 0.151_{(3)}$	$0.352 \pm 0.041_{(3)}$	$0.758 \pm 0.004_{(3)}$	$0.740 \pm 0.004_{(3)}$
DINOv2 ViT-G/14	$0.496 \pm 0.229_{(3)}$	$0.545 \pm 0.145_{(3)}$	$0.767 \pm 0.001_{(3)}$	$0.742 \pm 0.014_{(3)}$
DINOv2 ViT-G/14 REG	$0.427 \pm 0.281_{(3)}$	$0.585 \pm 0.327_{(3)}$	$0.747 \pm 0.002_{(3)}$	$0.716 \pm 0.012_{(3)}$
DINOv2 ViT-S/14	$0.505 \pm 0.181_{(3)}$	$0.569 \pm 0.099_{(3)}$	$0.763 \pm 0.001_{(3)}$	$0.746 \pm 0.010_{(3)}$
DINOv2 ViT-S/14 REG	$0.636 \pm 0.073_{(3)}$	$0.601 \pm 0.192_{(3)}$	$0.769 \pm 0.004_{(3)}$	$0.746 \pm 0.009_{(3)}$
LoRA-DINORET	$0.294 \pm 0.097_{(3)}$	$0.656 \pm 0.121_{(3)}$	$0.770 \pm 0.003_{(3)}$	$0.751 \pm 0.008_{(3)}$
Prov-GigaPath	$0.521 \pm 0.155_{(3)}$	$0.537 \pm 0.103_{(3)}$	$0.740 \pm 0.005_{(3)}$	$0.720 \pm 0.008_{(3)}$
RadDINO	$0.534 \pm 0.088_{(3)}$	$0.560 \pm 0.032_{(3)}$	$0.761 \pm 0.004_{(3)}$	$0.721 \pm 0.002_{(3)}$
ResNet-50 (ImageNet)	$0.620 \pm 0.116_{(3)}$	$0.495 \pm 0.128_{(3)}$	$0.750 \pm 0.006_{(3)}$	$0.720 \pm 0.015_{(3)}$
ResNet-50 (Random)	$0.552 \pm 0.035_{(3)}$	$0.479 \pm 0.232_{(3)}$	$0.745 \pm 0.007_{(3)}$	$0.683 \pm 0.003_{(3)}$
RET-CLIP	$0.652 \pm 0.161_{(3)}$	$0.528 \pm 0.048_{(3)}$	$0.606 \pm 0.020_{(3)}$	$0.536 \pm 0.006_{(3)}$
RETFound DINOv2 (MEH)	$0.428 \pm 0.184_{(3)}$	$0.608 \pm 0.161_{(3)}$	$0.699 \pm 0.012_{(3)}$	$0.720 \pm 0.001_{(3)}$
RETFound DINOv2 (Shanghai)	$0.586 \pm 0.088_{(3)}$	$0.496 \pm 0.079_{(3)}$	$0.767 \pm 0.006_{(3)}$	$0.739 \pm 0.001_{(3)}$
RETFound MAE (MEH)	$0.488 \pm 0.126_{(3)}$	$0.696 \pm 0.016_{(3)}$	$0.758 \pm 0.005_{(3)}$	$0.733 \pm 0.006_{(3)}$
RETFound MAE (Nature CFP)	$0.603 \pm 0.287_{(3)}$	$0.665 \pm 0.226_{(3)}$	$0.768 \pm 0.005_{(3)}$	$0.727 \pm 0.007_{(3)}$
RETFound MAE (Nature OCT)	$0.544 \pm 0.101_{(3)}$	$0.360 \pm 0.095_{(3)}$	$0.688 \pm 0.008_{(3)}$	$0.646 \pm 0.008_{(3)}$
RETFound MAE (Shanghai)	$0.564 \pm 0.193_{(3)}$	$0.543 \pm 0.277_{(3)}$	$0.764 \pm 0.009_{(3)}$	$0.724 \pm 0.005_{(3)}$
RETFound Green	$0.411 \pm 0.205_{(3)}$	$0.517 \pm 0.349_{(3)}$	$0.752 \pm 0.004_{(3)}$	$0.728 \pm 0.016_{(3)}$
RETIZero	$0.341 \pm 0.090_{(3)}$	$0.490 \pm 0.268_{(3)}$	$0.747 \pm 0.006_{(3)}$	$0.726 \pm 0.004_{(3)}$
SigLIP	$0.395 \pm 0.125_{(3)}$	$0.577 \pm 0.173_{(3)}$	$0.770 \pm 0.005_{(3)}$	$0.737 \pm 0.000_{(3)}$
SigLIP2	$0.447 \pm 0.259_{(3)}$	$0.641 \pm 0.092_{(3)}$	$0.727 \pm 0.008_{(3)}$	$0.703 \pm 0.008_{(3)}$
URFound	$0.499 \pm 0.278_{(3)}$	$0.600 \pm 0.038_{(3)}$	$0.773 \pm 0.007_{(3)}$	$0.749 \pm 0.005_{(3)}$
ViLRef	$0.652 \pm 0.161_{(3)}$	$0.528 \pm 0.048_{(3)}$	$0.606 \pm 0.020_{(3)}$	$0.536 \pm 0.006_{(3)}$
Virchow	$0.444 \pm 0.122_{(3)}$	$0.617 \pm 0.082_{(3)}$	$0.762 \pm 0.006_{(3)}$	$0.745 \pm 0.007_{(3)}$
Virchow2	$0.377 \pm 0.154_{(3)}$	$0.505 \pm 0.158_{(3)}$	$0.762 \pm 0.002_{(3)}$	$0.742 \pm 0.012_{(3)}$
VisionFM Fundus	$0.534 \pm 0.073_{(3)}$	$0.521 \pm 0.232_{(3)}$	$0.735 \pm 0.006_{(3)}$	$0.709 \pm 0.007_{(3)}$
VisionFM OCT	$0.663 \pm 0.192_{(3)}$	$0.671 \pm 0.207_{(3)}$	$0.780 \pm 0.004_{(3)}$	$0.723 \pm 0.011_{(3)}$
ViT-B/16 CLIP	$0.617 \pm 0.251_{(3)}$	$0.490 \pm 0.281_{(3)}$	$0.764 \pm 0.010_{(3)}$	$0.723 \pm 0.003_{(3)}$
ViT-B/32 CLIP	$0.468 \pm 0.124_{(3)}$	$0.529 \pm 0.252_{(3)}$	$0.755 \pm 0.012_{(3)}$	$0.724 \pm 0.005_{(3)}$
ViT-L/14 CLIP	$0.623 \pm 0.185_{(3)}$	$0.688 \pm 0.085_{(3)}$	$0.771 \pm 0.000_{(3)}$	$0.747 \pm 0.008_{(3)}$
ViT-B (ImageNet)	$0.375 \pm 0.135_{(3)}$	$0.529 \pm 0.158_{(3)}$	$0.758 \pm 0.008_{(3)}$	$0.727 \pm 0.010_{(3)}$

Table A.5: Performance comparison across datasets using full training. Values show test accuracy as mean ± standard deviation. – indicates no data available. Subscript numbers in parentheses indicate sample size.

Model	Harvard FairGenMed Vision Loss	Harvard FairVision AMD	Harvard FairVision DR	Harvard FairVision Glaucoma
BE-DINORET	$0.486 \pm 0.012_{(3)}$	$0.674 \pm 0.032_{(3)}$	$0.537 \pm 0.039_{(3)}$	$0.488 \pm 0.021_{(3)}$
DINORET	$0.451 \pm 0.035_{(3)}$	$0.704 \pm 0.073_{(3)}$	$0.519 \pm 0.263_{(3)}$	$0.501 \pm 0.014_{(3)}$
DINOv2 ViT-B/14	$0.485 \pm 0.009_{(3)}$	$0.668 \pm 0.025_{(3)}$	$0.517 \pm 0.075_{(3)}$	$0.490 \pm 0.013_{(3)}$
DINOv2 ViT-B/14 REG	$0.501 \pm 0.014_{(3)}$	$0.677 \pm 0.029_{(3)}$	$0.473 \pm 0.045_{(3)}$	$0.493 \pm 0.001_{(3)}$
DINOv2 ViT-G/14	$0.487 \pm 0.039_{(3)}$	$0.674 \pm 0.029_{(3)}$	$0.452 \pm 0.154_{(3)}$	$0.507 \pm 0.024_{(3)}$
DINOv2 ViT-G/14 REG	$0.496 \pm 0.031_{(3)}$	$0.687 \pm 0.036_{(3)}$	$0.536 \pm 0.106_{(3)}$	$0.493 \pm 0.003_{(3)}$
DINOv2 ViT-S/14	$0.483 \pm 0.061_{(3)}$	$0.672 \pm 0.055_{(3)}$	$0.364 \pm 0.221_{(3)}$	$0.495 \pm 0.014_{(3)}$
DINOv2 ViT-S/14 REG	$0.500 \pm 0.045_{(3)}$	$0.712 \pm 0.029_{(3)}$	$0.537 \pm 0.088_{(3)}$	$0.508 \pm 0.006_{(3)}$
LoRA-DINORET	$0.525 \pm 0.029_{(3)}$	$0.705 \pm 0.080_{(3)}$	$0.412 \pm 0.256_{(3)}$	$0.504 \pm 0.017_{(3)}$
Prov-GigaPath	$0.487 \pm 0.016_{(3)}$	$0.674 \pm 0.025_{(3)}$	$0.466 \pm 0.201_{(3)}$	$0.495 \pm 0.009_{(3)}$
RadDINO	$0.441 \pm 0.057_{(3)}$	$0.716 \pm 0.023_{(3)}$	$0.512 \pm 0.388_{(3)}$	$0.498 \pm 0.015_{(3)}$
ResNet-50 (ImageNet)	$0.477 \pm 0.003_{(3)}$	$0.675 \pm 0.053_{(3)}$	$0.614 \pm 0.119_{(3)}$	$0.492 \pm 0.014_{(3)}$
ResNet-50 (Random)	$0.459 \pm 0.019_{(3)}$	$0.689 \pm 0.012_{(3)}$	$0.489 \pm 0.213_{(3)}$	$0.499 \pm 0.017_{(3)}$
RET-CLIP	$0.296 \pm 0.002_{(3)}$	$0.200 \pm 0.094_{(3)}$	$0.359 \pm 0.455_{(3)}$	$0.495 \pm 0.011_{(3)}$
RETFound DINOv2 (MEH)	$0.488 \pm 0.011_{(3)}$	$0.574 \pm 0.035_{(3)}$	$0.529 \pm 0.019_{(3)}$	$0.510 \pm 0.012_{(3)}$
RETFound DINOv2 (Shanghai)	$0.454 \pm 0.006_{(3)}$	$0.695 \pm 0.090_{(3)}$	$0.525 \pm 0.192_{(3)}$	$0.479 \pm 0.012_{(3)}$
RETFound MAE (MEH)	$0.516 \pm 0.029_{(3)}$	$0.674 \pm 0.027_{(3)}$	$0.562 \pm 0.319_{(3)}$	$0.491 \pm 0.024_{(3)}$
RETFound MAE (Nature CFP)	$0.492 \pm 0.013_{(3)}$	$0.654 \pm 0.084_{(3)}$	$0.403 \pm 0.371_{(3)}$	$0.485 \pm 0.002_{(3)}$
RETFound MAE (Nature OCT)	$0.438 \pm 0.022_{(3)}$	$0.592 \pm 0.009_{(3)}$	$0.398 \pm 0.123_{(3)}$	$0.498 \pm 0.015_{(3)}$
RETFound MAE (Shanghai)	$0.498 \pm 0.031_{(3)}$	$0.547 \pm 0.026_{(3)}$	$0.502 \pm 0.409_{(3)}$	$0.506 \pm 0.017_{(3)}$
RETFound Green	$0.449 \pm 0.012_{(3)}$	$0.663 \pm 0.063_{(3)}$	$0.464 \pm 0.170_{(3)}$	$0.480 \pm 0.007_{(3)}$
RETIZero	$0.507 \pm 0.040_{(3)}$	$0.624 \pm 0.021_{(3)}$	$0.354 \pm 0.258_{(3)}$	$0.490 \pm 0.003_{(3)}$
SigLIP	$0.533 \pm 0.017_{(3)}$	$0.695 \pm 0.024_{(3)}$	$0.571 \pm 0.178_{(3)}$	$0.495 \pm 0.015_{(3)}$
SigLIP2	$0.473 \pm 0.013_{(3)}$	$0.622 \pm 0.023_{(3)}$	$0.657 \pm 0.334_{(3)}$	$0.498 \pm 0.008_{(3)}$
URFound	$0.533 \pm 0.023_{(3)}$	$0.730 \pm 0.032_{(3)}$	$0.449 \pm 0.338_{(3)}$	$0.500 \pm 0.011_{(3)}$
ViLRef	$0.296 \pm 0.002_{(3)}$	$0.200 \pm 0.094_{(3)}$	$0.359 \pm 0.455_{(3)}$	$0.495 \pm 0.011_{(3)}$
Virchow	$0.493 \pm 0.056_{(3)}$	$0.712 \pm 0.065_{(3)}$	$0.727 \pm 0.214_{(3)}$	$0.491 \pm 0.005_{(3)}$
Virchow2	$0.484 \pm 0.008_{(3)}$	$0.683 \pm 0.053_{(3)}$	$0.522 \pm 0.167_{(3)}$	$0.490 \pm 0.010_{(3)}$
VisionFM Fundus	$0.440 \pm 0.030_{(3)}$	$0.592 \pm 0.066_{(3)}$	$0.550 \pm 0.211_{(3)}$	$0.501 \pm 0.014_{(3)}$
VisionFM OCT	$0.486 \pm 0.023_{(3)}$	$0.684 \pm 0.019_{(3)}$	$0.381 \pm 0.090_{(3)}$	$0.488 \pm 0.001_{(3)}$
ViT-B/16 CLIP	$0.487 \pm 0.020_{(3)}$	$0.664 \pm 0.067_{(3)}$	$0.571 \pm 0.134_{(3)}$	$0.493 \pm 0.005_{(3)}$
ViT-B/32 CLIP	$0.475 \pm 0.015_{(3)}$	$0.666 \pm 0.021_{(3)}$	$0.484 \pm 0.063_{(3)}$	$0.495 \pm 0.006_{(3)}$
ViT-L/14 CLIP	$0.502 \pm 0.025_{(3)}$	$0.636 \pm 0.059_{(3)}$	$0.531 \pm 0.139_{(3)}$	$0.502 \pm 0.018_{(3)}$
ViT-B (ImageNet)	$0.489 \pm 0.029_{(3)}$	$0.668 \pm 0.052_{(3)}$	$0.534 \pm 0.263_{(3)}$	$0.494 \pm 0.013_{(3)}$

Table A.6: Performance comparison across datasets using full training. Values show test accuracy as mean ± standard deviation. – indicates no data available. Subscript numbers in parentheses indicate sample size.

**Model**	**Harvard FairVision Multiclass**	**Harvard GF Glaucoma**	**Harvard GF MD**	**HRF**
BE-DINORET	$0.349 \pm 0.011_{(3)}$	$0.682 \pm 0.010_{(3)}$	$0.434 \pm 0.016_{(3)}$	$0.524 \pm 0.218_{(3)}$
DINORET	$0.373 \pm 0.017_{(3)}$	$0.712 \pm 0.017_{(3)}$	$0.467 \pm 0.036_{(3)}$	$0.524 \pm 0.082_{(3)}$
DINOv2 ViT-B/14	$0.339 \pm 0.008_{(3)}$	$0.683 \pm 0.008_{(3)}$	$0.425 \pm 0.008_{(3)}$	$0.667 \pm 0.082_{(3)}$
DINOv2 ViT-B/14 REG	$0.349 \pm 0.040_{(3)}$	$0.700 \pm 0.023_{(3)}$	$0.424 \pm 0.017_{(3)}$	$0.619 \pm 0.082_{(3)}$
DINOv2 ViT-G/14	$0.326 \pm 0.032_{(3)}$	$0.685 \pm 0.005_{(3)}$	$0.316 \pm 0.104_{(3)}$	$0.571 \pm 0.143_{(3)}$
DINOv2 ViT-G/14 REG	$0.343 \pm 0.028_{(3)}$	$0.692 \pm 0.018_{(3)}$	$0.441 \pm 0.021_{(3)}$	$0.619 \pm 0.218_{(3)}$
DINOv2 ViT-S/14	$0.383 \pm 0.016_{(3)}$	$0.700 \pm 0.010_{(3)}$	$0.386 \pm 0.067_{(3)}$	$0.619 \pm 0.082_{(3)}$
DINOv2 ViT-S/14 REG	$0.373 \pm 0.045_{(3)}$	$0.702 \pm 0.011_{(3)}$	$0.453 \pm 0.030_{(3)}$	$0.524 \pm 0.082_{(3)}$
LoRA-DINORET	$0.344 \pm 0.057_{(3)}$	$0.710 \pm 0.025_{(3)}$	$0.442 \pm 0.022_{(3)}$	$0.714 \pm 0.143_{(3)}$
Prov-GigaPath	$0.347 \pm 0.005_{(3)}$	$0.691 \pm 0.010_{(3)}$	$0.413 \pm 0.013_{(3)}$	$0.714_{(3)}$
RadDINO	$0.347 \pm 0.065_{(3)}$	$0.704 \pm 0.002_{(3)}$	$0.415 \pm 0.013_{(3)}$	$0.571_{(3)}$
ResNet-50 (ImageNet)	$0.361 \pm 0.006_{(3)}$	$0.706 \pm 0.030_{(3)}$	$0.430 \pm 0.018_{(3)}$	$0.524 \pm 0.082_{(3)}$
ResNet-50 (Random)	$0.334 \pm 0.034_{(3)}$	$0.710 \pm 0.011_{(3)}$	$0.430 \pm 0.039_{(3)}$	$0.667 \pm 0.165_{(3)}$
RET-CLIP	$0.305 \pm 0.124_{(3)}$	$0.597 \pm 0.025_{(3)}$	$0.290 \pm 0.019_{(3)}$	$0.286 \pm 0.143_{(3)}$
RETFound DINOv2 (MEH)	$0.323 \pm 0.035_{(3)}$	$0.640 \pm 0.018_{(3)}$	$0.372 \pm 0.047_{(3)}$	$0.762 \pm 0.082_{(3)}$
RETFound DINOv2 (Shanghai)	$0.352 \pm 0.034_{(3)}$	$0.706 \pm 0.013_{(3)}$	$0.440 \pm 0.022_{(3)}$	$0.667 \pm 0.082_{(3)}$
RETFound MAE (MEH)	$0.365 \pm 0.009_{(3)}$	$0.714 \pm 0.015_{(3)}$	$0.456 \pm 0.011_{(3)}$	$0.762 \pm 0.082_{(3)}$
RETFound MAE (Nature CFP)	$0.366 \pm 0.009_{(3)}$	$0.710 \pm 0.013_{(3)}$	$0.466 \pm 0.012_{(3)}$	$0.667 \pm 0.082_{(3)}$
RETFound MAE (Nature OCT)	$0.322 \pm 0.039_{(3)}$	$0.715 \pm 0.021_{(3)}$	$0.439 \pm 0.020_{(3)}$	$0.429 \pm 0.143_{(3)}$
RETFound MAE (Shanghai)	$0.378 \pm 0.004_{(3)}$	$0.704 \pm 0.003_{(3)}$	$0.445 \pm 0.020_{(3)}$	$0.619 \pm 0.082_{(3)}$
RETFound Green	$0.359 \pm 0.028_{(3)}$	$0.692 \pm 0.017_{(3)}$	$0.444 \pm 0.006_{(3)}$	$0.476 \pm 0.082_{(3)}$
RETIZero	$0.361 \pm 0.007_{(3)}$	$0.706 \pm 0.007_{(3)}$	$0.441 \pm 0.018_{(3)}$	$0.714 \pm 0.143_{(3)}$
SigLIP	$0.360 \pm 0.046_{(3)}$	$0.714 \pm 0.008_{(3)}$	$0.440 \pm 0.044_{(3)}$	$0.667 \pm 0.082_{(3)}$
SigLIP2	$0.346 \pm 0.021_{(3)}$	$0.657 \pm 0.016_{(3)}$	$0.392 \pm 0.034_{(3)}$	$0.905 \pm 0.082_{(3)}$
URFound	$0.383 \pm 0.005_{(3)}$	$0.731 \pm 0.002_{(3)}$	$0.499 \pm 0.009_{(3)}$	$0.714_{(3)}$
ViLRef	$0.305 \pm 0.124_{(3)}$	$0.597 \pm 0.025_{(3)}$	$0.290 \pm 0.019_{(3)}$	$0.286 \pm 0.143_{(3)}$
Virchow	$0.373 \pm 0.009_{(3)}$	$0.702 \pm 0.010_{(3)}$	$0.478 \pm 0.020_{(3)}$	$0.571 \pm 0.143_{(3)}$
Virchow2	$0.361 \pm 0.041_{(3)}$	$0.718 \pm 0.008_{(3)}$	$0.464 \pm 0.028_{(3)}$	$0.714 \pm 0.143_{(3)}$
VisionFM Fundus	$0.325 \pm 0.019_{(3)}$	$0.674 \pm 0.015_{(3)}$	$0.387 \pm 0.037_{(3)}$	$0.571_{(3)}$
VisionFM OCT	$0.321 \pm 0.026_{(3)}$	$0.736 \pm 0.002_{(3)}$	$0.458 \pm 0.058_{(3)}$	$0.571 \pm 0.143_{(3)}$
ViT-B/16 CLIP	$0.323 \pm 0.016_{(3)}$	$0.702 \pm 0.025_{(3)}$	$0.413 \pm 0.063_{(3)}$	$0.619 \pm 0.165_{(3)}$
ViT-B/32 CLIP	$0.340 \pm 0.038_{(3)}$	$0.690 \pm 0.008_{(3)}$	$0.431 \pm 0.033_{(3)}$	$0.619 \pm 0.165_{(3)}$
ViT-L/14 CLIP	$0.352 \pm 0.035_{(3)}$	$0.712 \pm 0.028_{(3)}$	$0.410 \pm 0.022_{(3)}$	$0.571 \pm 0.143_{(3)}$
ViT-B (ImageNet)	$0.385 \pm 0.007_{(3)}$	$0.718 \pm 0.019_{(3)}$	$0.439 \pm 0.031_{(3)}$	$0.714 \pm 0.143_{(3)}$

Table A.7: Performance comparison across datasets using full training. Values show test accuracy as mean ± standard deviation. – indicates no data available. Subscript numbers in parentheses indicate sample size.

Model	IDRiD	IDRiD ME	JustRAIGS	MBRSET DR
BE-DINORET	$0.482 \pm 0.077_{(3)}$	$0.699 \pm 0.035_{(3)}$	$0.856 \pm 0.010_{(3)}$	$0.437 \pm 0.030_{(3)}$
DINORET	$0.473 \pm 0.076_{(3)}$	$0.691 \pm 0.026_{(3)}$	$0.883 \pm 0.019_{(3)}$	$0.391 \pm 0.147_{(3)}$
DINOv2 ViT-B/14	$0.507 \pm 0.061_{(3)}$	$0.700 \pm 0.019_{(3)}$	$0.857 \pm 0.005_{(3)}$	$0.418 \pm 0.023_{(3)}$
DINOv2 ViT-B/14 REG	$0.494 \pm 0.062_{(3)}$	$0.691 \pm 0.057_{(3)}$	$0.896 \pm 0.006_{(3)}$	$0.411 \pm 0.089_{(3)}$
DINOv2 ViT-G/14	$0.460 \pm 0.067_{(3)}$	$0.692 \pm 0.044_{(3)}$	$0.897 \pm 0.006_{(3)}$	$0.318 \pm 0.138_{(3)}$
DINOv2 ViT-G/14 REG	$0.447 \pm 0.004_{(3)}$	$0.722 \pm 0.039_{(3)}$	$0.882 \pm 0.006_{(3)}$	$0.352 \pm 0.102_{(3)}$
DINOv2 ViT-S/14	$0.443 \pm 0.073_{(3)}$	$0.699 \pm 0.072_{(3)}$	$0.904 \pm 0.011_{(3)}$	$0.407 \pm 0.069_{(3)}$
DINOv2 ViT-S/14 REG	$0.498 \pm 0.047_{(3)}$	$0.718 \pm 0.059_{(3)}$	$0.906 \pm 0.019_{(3)}$	$0.429 \pm 0.195_{(3)}$
LoRA-DINORET	$0.477 \pm 0.046_{(3)}$	$0.740 \pm 0.027_{(3)}$	$0.920 \pm 0.008_{(3)}$	$0.444 \pm 0.022_{(3)}$
Prov-GigaPath	$0.532 \pm 0.083_{(3)}$	$0.752 \pm 0.041_{(3)}$	$0.816 \pm 0.007_{(3)}$	$0.512 \pm 0.053_{(3)}$
RadDINO	$0.426 \pm 0.026_{(3)}$	$0.691 \pm 0.033_{(3)}$	$0.883 \pm 0.006_{(3)}$	$0.437 \pm 0.113_{(3)}$
ResNet-50 (ImageNet)	$0.410 \pm 0.063_{(3)}$	$0.743 \pm 0.057_{(3)}$	$0.880 \pm 0.008_{(3)}$	$0.455 \pm 0.056_{(3)}$
ResNet-50 (Random)	$0.219 \pm 0.048_{(3)}$	$0.504 \pm 0.064_{(3)}$	$0.816 \pm 0.013_{(3)}$	$0.152 \pm 0.146_{(3)}$
RET-CLIP	$0.261 \pm 0.064_{(3)}$	$0.449 \pm 0.068_{(3)}$	$0.611 \pm 0.035_{(3)}$	$0.200 \pm 0.091_{(3)}$
RETFound DINOv2 (MEH)	$0.439 \pm 0.059_{(3)}$	$0.696 \pm 0.032_{(3)}$	$0.902 \pm 0.003_{(3)}$	$0.468 \pm 0.038_{(3)}$
RETFound DINOv2 (Shanghai)	$0.435 \pm 0.030_{(3)}$	$0.722 \pm 0.015_{(3)}$	$0.922 \pm 0.002_{(3)}$	$0.424 \pm 0.028_{(3)}$
RETFound MAE (MEH)	$0.444 \pm 0.125_{(3)}$	$0.696 \pm 0.059_{(3)}$	$0.882 \pm 0.005_{(3)}$	$0.545 \pm 0.068_{(3)}$
RETFound MAE (Nature CFP)	$0.494 \pm 0.061_{(3)}$	$0.661 \pm 0.052_{(3)}$	$0.889 \pm 0.006_{(3)}$	$0.543 \pm 0.161_{(3)}$
RETFound MAE (Nature OCT)	$0.261 \pm 0.024_{(3)}$	$0.531 \pm 0.086_{(3)}$	$0.668 \pm 0.014_{(3)}$	$0.297 \pm 0.033_{(3)}$
RETFound MAE (Shanghai)	$0.481 \pm 0.067_{(3)}$	$0.705 \pm 0.034_{(3)}$	$0.902 \pm 0.005_{(3)}$	$0.446 \pm 0.051_{(3)}$
RETFound Green	$0.477 \pm 0.097_{(3)}$	$0.717 \pm 0.068_{(3)}$	$0.880 \pm 0.006_{(3)}$	$0.379 \pm 0.066_{(3)}$
RETIZero	$0.540 \pm 0.081_{(3)}$	$0.761 \pm 0.034_{(3)}$	$0.904 \pm 0.012_{(3)}$	$0.574 \pm 0.124_{(3)}$
SigLIP	$0.472 \pm 0.013_{(3)}$	$0.709 \pm 0.032_{(3)}$	$0.895 \pm 0.013_{(3)}$	$0.437 \pm 0.278_{(3)}$
SigLIP2	$0.460 \pm 0.105_{(3)}$	$0.688 \pm 0.059_{(3)}$	$0.842 \pm 0.005_{(3)}$	$0.442 \pm 0.115_{(3)}$
URFound	$0.498 \pm 0.157_{(3)}$	$0.766 \pm 0.028_{(3)}$	$0.900 \pm 0.006_{(3)}$	$0.504 \pm 0.102_{(3)}$
ViLRef	$0.261 \pm 0.064_{(3)}$	$0.449 \pm 0.068_{(3)}$	$0.611 \pm 0.035_{(3)}$	$0.200 \pm 0.091_{(3)}$
Virchow	$0.473 \pm 0.079_{(3)}$	$0.709 \pm 0.026_{(3)}$	$0.915 \pm 0.011_{(3)}$	$0.494 \pm 0.242_{(3)}$
Virchow2	$0.422 \pm 0.042_{(3)}$	$0.707 \pm 0.068_{(3)}$	$0.881 \pm 0.012_{(3)}$	$0.549 \pm 0.085_{(3)}$
VisionFM Fundus	$0.384 \pm 0.049_{(3)}$	$0.626 \pm 0.060_{(3)}$	$0.890 \pm 0.005_{(3)}$	$0.397 \pm 0.051_{(3)}$
VisionFM OCT	$0.384 \pm 0.045_{(3)}$	$0.631 \pm 0.043_{(3)}$	$0.867 \pm 0.024_{(3)}$	$0.197 \pm 0.118_{(3)}$
ViT-B/16 CLIP	$0.481 \pm 0.039_{(3)}$	$0.722 \pm 0.071_{(3)}$	$0.888 \pm 0.002_{(3)}$	$0.494 \pm 0.069_{(3)}$
ViT-B/32 CLIP	$0.447 \pm 0.016_{(3)}$	$0.661 \pm 0.019_{(3)}$	$0.869 \pm 0.006_{(3)}$	$0.432 \pm 0.091_{(3)}$
ViT-L/14 CLIP	$0.431 \pm 0.114_{(3)}$	$0.730 \pm 0.038_{(3)}$	$0.906 \pm 0.012_{(3)}$	$0.389 \pm 0.175_{(3)}$
ViT-B (ImageNet)	$0.490 \pm 0.013_{(3)}$	$0.687 \pm 0.031_{(3)}$	$0.877 \pm 0.024_{(3)}$	$0.399 \pm 0.025_{(3)}$

Table A.8: Performance comparison across datasets using full training. Values show test accuracy as mean ± standard deviation. – indicates no data available. Subscript numbers in parentheses indicate sample size.

Model	MBRSET HT	MBRSET Microvascular	MBRSET Obesity	MESSIDOR
BE-DINORET	$0.506 \pm 0.040_{(3)}$	$0.575 \pm 0.008_{(3)}$	$0.551 \pm 0.079_{(3)}$	$0.427 \pm 0.059_{(3)}$
DINORET	$0.552 \pm 0.116_{(3)}$	$0.538 \pm 0.100_{(3)}$	$0.660 \pm 0.134_{(3)}$	$0.434 \pm 0.113_{(3)}$
DINOv2 ViT-B/14	$0.594 \pm 0.032_{(3)}$	$0.602 \pm 0.007_{(3)}$	$0.601 \pm 0.111_{(3)}$	$0.435 \pm 0.060_{(3)}$
DINOv2 ViT-B/14 REG	$0.550 \pm 0.012_{(3)}$	$0.538 \pm 0.052_{(3)}$	$0.491 \pm 0.158_{(3)}$	$0.344 \pm 0.030_{(3)}$
DINOv2 ViT-G/14	$0.411 \pm 0.120_{(3)}$	$0.592 \pm 0.054_{(3)}$	$0.210 \pm 0.082_{(3)}$	$0.338 \pm 0.088_{(3)}$
DINOv2 ViT-G/14 REG	$0.569 \pm 0.067_{(3)}$	$0.511 \pm 0.082_{(3)}$	$0.444 \pm 0.064_{(3)}$	$0.427 \pm 0.051_{(3)}$
DINOv2 ViT-S/14	$0.605 \pm 0.051_{(3)}$	$0.532 \pm 0.038_{(3)}$	$0.611 \pm 0.254_{(3)}$	$0.434 \pm 0.097_{(3)}$
DINOv2 ViT-S/14 REG	$0.530 \pm 0.030_{(3)}$	$0.553 \pm 0.076_{(3)}$	$0.460 \pm 0.139_{(3)}$	$0.365 \pm 0.023_{(3)}$
LoRA-DINORET	$0.497 \pm 0.058_{(3)}$	$0.527 \pm 0.021_{(3)}$	$0.445 \pm 0.115_{(3)}$	$0.419 \pm 0.066_{(3)}$
Prov-GigaPath	$0.531 \pm 0.025_{(3)}$	$0.556 \pm 0.026_{(3)}$	$0.576 \pm 0.185_{(3)}$	$0.444 \pm 0.043_{(3)}$
RadDINO	$0.532 \pm 0.030_{(3)}$	$0.500 \pm 0.027_{(3)}$	$0.558 \pm 0.082_{(3)}$	$0.326 \pm 0.099_{(3)}$
ResNet-50 (ImageNet)	$0.603 \pm 0.018_{(3)}$	$0.532 \pm 0.018_{(3)}$	$0.482 \pm 0.215_{(3)}$	$0.424 \pm 0.040_{(3)}$
ResNet-50 (Random)	$0.502 \pm 0.120_{(3)}$	$0.426 \pm 0.042_{(3)}$	$0.616 \pm 0.155_{(3)}$	$0.290 \pm 0.088_{(3)}$
RET-CLIP	$0.533 \pm 0.054_{(3)}$	$0.519 \pm 0.077_{(3)}$	$0.380 \pm 0.317_{(3)}$	$0.204 \pm 0.169_{(3)}$
RETFound DINOv2 (MEH)	$0.552 \pm 0.034_{(3)}$	$0.507 \pm 0.024_{(3)}$	$0.462 \pm 0.012_{(3)}$	$0.435 \pm 0.077_{(3)}$
RETFound DINOv2 (Shanghai)	$0.563 \pm 0.018_{(3)}$	$0.514 \pm 0.015_{(3)}$	$0.450 \pm 0.043_{(3)}$	$0.405 \pm 0.067_{(3)}$
RETFound MAE (MEH)	$0.596 \pm 0.034_{(3)}$	$0.554 \pm 0.026_{(3)}$	$0.705 \pm 0.189_{(3)}$	$0.422 \pm 0.078_{(3)}$
RETFound MAE (Nature CFP)	$0.549 \pm 0.092_{(3)}$	$0.503 \pm 0.072_{(3)}$	$0.329 \pm 0.398_{(3)}$	$0.439 \pm 0.103_{(3)}$
RETFound MAE (Nature OCT)	$0.504 \pm 0.033_{(3)}$	$0.497 \pm 0.061_{(3)}$	$0.620 \pm 0.215_{(3)}$	$0.248 \pm 0.054_{(3)}$
RETFound MAE (Shanghai)	$0.620 \pm 0.046_{(3)}$	$0.540 \pm 0.042_{(3)}$	$0.545 \pm 0.134_{(3)}$	$0.486 \pm 0.081_{(3)}$
RETFound Green	$0.543 \pm 0.081_{(3)}$	$0.565 \pm 0.019_{(3)}$	$0.350 \pm 0.173_{(3)}$	$0.449 \pm 0.075_{(3)}$
RETIZero	$0.598 \pm 0.069_{(3)}$	$0.595 \pm 0.027_{(3)}$	$0.612 \pm 0.203_{(3)}$	$0.458 \pm 0.071_{(3)}$
SigLIP	$0.580 \pm 0.171_{(3)}$	$0.530 \pm 0.045_{(3)}$	$0.562 \pm 0.357_{(3)}$	$0.500 \pm 0.080_{(3)}$
SigLIP2	$0.511 \pm 0.031_{(3)}$	$0.554 \pm 0.059_{(3)}$	$0.686 \pm 0.231_{(3)}$	$0.431 \pm 0.099_{(3)}$
URFound	$0.556 \pm 0.043_{(3)}$	$0.522 \pm 0.078_{(3)}$	$0.489 \pm 0.276_{(3)}$	$0.475 \pm 0.057_{(3)}$
ViLRef	$0.533 \pm 0.054_{(3)}$	$0.519 \pm 0.077_{(3)}$	$0.380 \pm 0.317_{(3)}$	$0.204 \pm 0.169_{(3)}$
Virchow	$0.446 \pm 0.021_{(3)}$	$0.509 \pm 0.038_{(3)}$	$0.540 \pm 0.193_{(3)}$	$0.416 \pm 0.099_{(3)}$
Virchow2	$0.479 \pm 0.102_{(3)}$	$0.557 \pm 0.031_{(3)}$	$0.540 \pm 0.191_{(3)}$	$0.453 \pm 0.086_{(3)}$
VisionFM Fundus	$0.517 \pm 0.023_{(3)}$	$0.527 \pm 0.046_{(3)}$	$0.445 \pm 0.044_{(3)}$	$0.338 \pm 0.015_{(3)}$
VisionFM OCT	$0.557 \pm 0.040_{(3)}$	$0.449 \pm 0.096_{(3)}$	$0.405 \pm 0.234_{(3)}$	$0.253 \pm 0.053_{(3)}$
ViT-B/16 CLIP	$0.542 \pm 0.080_{(3)}$	$0.554 \pm 0.047_{(3)}$	$0.451 \pm 0.046_{(3)}$	$0.338 \pm 0.032_{(3)}$
ViT-B/32 CLIP	$0.533 \pm 0.024_{(3)}$	$0.558 \pm 0.035_{(3)}$	$0.405 \pm 0.003_{(3)}$	$0.377 \pm 0.056_{(3)}$
ViT-L/14 CLIP	$0.628 \pm 0.067_{(3)}$	$0.553 \pm 0.047_{(3)}$	$0.630 \pm 0.194_{(3)}$	$0.318 \pm 0.055_{(3)}$
ViT-B (ImageNet)	$0.579 \pm 0.053_{(3)}$	$0.542 \pm 0.062_{(3)}$	$0.449 \pm 0.091_{(3)}$	$0.364 \pm 0.061_{(3)}$

Table A.9: Performance comparison across datasets using full training. Values show test accuracy as mean ± standard deviation. – indicates no data available. Subscript numbers in parentheses indicate sample size.

Model	MESSIDOR DME	OCT X-ray	OCTID	OUWFFI
BE-DINORET	$0.852 \pm 0.048_{(3)}$	$0.886 \pm 0.021_{(3)}$	$0.903 \pm 0.036_{(3)}$	$0.758 \pm 0.005_{(3)}$
DINORET	$0.879 \pm 0.041_{(3)}$	$0.948 \pm 0.015_{(3)}$	$0.903 \pm 0.036_{(3)}$	$0.755 \pm 0.053_{(3)}$
DINOv2 ViT-B/14	$0.864 \pm 0.037_{(3)}$	$0.898 \pm 0.009_{(3)}$	$0.911 \pm 0.024_{(3)}$	$0.774 \pm 0.025_{(3)}$
DINOv2 ViT-B/14 REG	$0.891 \pm 0.018_{(3)}$	$0.936 \pm 0.015_{(3)}$	$0.915 \pm 0.018_{(3)}$	$0.777 \pm 0.052_{(3)}$
DINOv2 ViT-G/14	$0.864 \pm 0.025_{(3)}$	$0.947 \pm 0.017_{(3)}$	$0.922 \pm 0.029_{(3)}$	$0.736 \pm 0.047_{(3)}$
DINOv2 ViT-G/14 REG	$0.846 \pm 0.040_{(3)}$	$0.911 \pm 0.012_{(3)}$	$0.907 \pm 0.042_{(3)}$	$0.774 \pm 0.025_{(3)}$
DINOv2 ViT-S/14	$0.875 \pm 0.056_{(3)}$	$0.945 \pm 0.017_{(3)}$	$0.953 \pm 0.020_{(3)}$	$0.780 \pm 0.022_{(3)}$
DINOv2 ViT-S/14 REG	$0.870 \pm 0.031_{(3)}$	$0.941 \pm 0.018_{(3)}$	$0.938 \pm 0.013_{(3)}$	$0.808 \pm 0.022_{(3)}$
LoRA-DINORET	$0.838 \pm 0.021_{(3)}$	$0.947 \pm 0.016_{(3)}$	$0.946 \pm 0.024_{(3)}$	$0.764 \pm 0.009_{(3)}$
Prov-GigaPath	$0.856 \pm 0.006_{(3)}$	$0.858 \pm 0.021_{(3)}$	$0.911 \pm 0.018_{(3)}$	$0.789 \pm 0.005_{(3)}$
RadDINO	$0.835 \pm 0.020_{(3)}$	$0.926 \pm 0.017_{(3)}$	$0.888 \pm 0.029_{(3)}$	$0.704 \pm 0.047_{(3)}$
ResNet-50 (ImageNet)	$0.873 \pm 0.018_{(3)}$	$0.922 \pm 0.022_{(3)}$	$0.926 \pm 0.036_{(3)}$	$0.761 \pm 0.014_{(3)}$
ResNet-50 (Random)	$0.659 \pm 0.143_{(3)}$	$0.907 \pm 0.016_{(3)}$	$0.810 \pm 0.047_{(3)}$	$0.632 \pm 0.038_{(3)}$
RET-CLIP	$0.599 \pm 0.052_{(3)}$	$0.406 \pm 0.057_{(3)}$	$0.500 \pm 0.012_{(3)}$	$0.327 \pm 0.038_{(3)}$
RETFound DINOv2 (MEH)	$0.860 \pm 0.024_{(3)}$	$0.802 \pm 0.035_{(3)}$	$0.860 \pm 0.012_{(3)}$	$0.755 \pm 0.019_{(3)}$
RETFound DINOv2 (Shanghai)	$0.887 \pm 0.010_{(3)}$	$0.936 \pm 0.021_{(3)}$	$0.946 \pm 0.007_{(3)}$	$0.808 \pm 0.043_{(3)}$
RETFound MAE (MEH)	$0.810 \pm 0.091_{(3)}$	$0.926 \pm 0.017_{(3)}$	$0.907 \pm 0.035_{(3)}$	$0.701 \pm 0.044_{(3)}$
RETFound MAE (Nature CFP)	$0.844 \pm 0.056_{(3)}$	$0.930 \pm 0.019_{(3)}$	$0.922 \pm 0.013_{(3)}$	$0.726 \pm 0.034_{(3)}$
RETFound MAE (Nature OCT)	$0.508 \pm 0.040_{(3)}$	$0.799 \pm 0.029_{(3)}$	$0.891 \pm 0.037_{(3)}$	$0.506 \pm 0.020_{(3)}$
RETFound MAE (Shanghai)	$0.837 \pm 0.042_{(3)}$	$0.932 \pm 0.020_{(3)}$	$0.915 \pm 0.029_{(3)}$	$0.770 \pm 0.014_{(3)}$
RETFound Green	$0.823 \pm 0.014_{(3)}$	$0.924 \pm 0.014_{(3)}$	$0.903 \pm 0.048_{(3)}$	$0.748 \pm 0.014_{(3)}$
RETIZero	$0.841 \pm 0.066_{(3)}$	$0.909 \pm 0.007_{(3)}$	$0.907 \pm 0.023_{(3)}$	$0.777 \pm 0.005_{(3)}$
SigLIP	$0.865 \pm 0.044_{(3)}$	$0.944 \pm 0.018_{(3)}$	$0.934 \pm 0.029_{(3)}$	$0.752 \pm 0.036_{(3)}$
SigLIP2	$0.861 \pm 0.033_{(3)}$	$0.890 \pm 0.015_{(3)}$	$0.903 \pm 0.071_{(3)}$	$0.742 \pm 0.024_{(3)}$
URFound	$0.880 \pm 0.021_{(3)}$	$0.942 \pm 0.023_{(3)}$	$0.919 \pm 0.020_{(3)}$	$0.748 \pm 0.052_{(3)}$
ViLRef	$0.599 \pm 0.052_{(3)}$	$0.406 \pm 0.057_{(3)}$	$0.500 \pm 0.012_{(3)}$	$0.327 \pm 0.038_{(3)}$
Virchow	$0.845 \pm 0.100_{(3)}$	$0.939 \pm 0.018_{(3)}$	$0.922 \pm 0.041_{(3)}$	$0.789 \pm 0.014_{(3)}$
Virchow2	$0.884 \pm 0.023_{(3)}$	$0.938 \pm 0.016_{(3)}$	$0.926 \pm 0.027_{(3)}$	$0.777 \pm 0.029_{(3)}$
VisionFM Fundus	$0.826 \pm 0.058_{(3)}$	$0.840 \pm 0.018_{(3)}$	$0.822 \pm 0.018_{(3)}$	$0.745 \pm 0.033_{(3)}$
VisionFM OCT	$0.630 \pm 0.126_{(3)}$	$0.938 \pm 0.015_{(3)}$	$0.942 \pm 0.020_{(3)}$	$0.591 \pm 0.086_{(3)}$
ViT-B/16 CLIP	$0.836 \pm 0.023_{(3)}$	$0.928 \pm 0.018_{(3)}$	$0.926 \pm 0.027_{(3)}$	$0.786 \pm 0.022_{(3)}$
ViT-B/32 CLIP	$0.796 \pm 0.028_{(3)}$	$0.921 \pm 0.013_{(3)}$	$0.915 \pm 0.047_{(3)}$	$0.758 \pm 0.020_{(3)}$
ViT-L/14 CLIP	$0.851 \pm 0.030_{(3)}$	$0.944 \pm 0.017_{(3)}$	$0.942 \pm 0.031_{(3)}$	$0.777 \pm 0.014_{(3)}$
ViT-B (ImageNet)	$0.793 \pm 0.015_{(3)}$	$0.932 \pm 0.027_{(3)}$	$0.942 \pm 0.031_{(3)}$	$0.758 \pm 0.024_{(3)}$

Table A.10: Performance comparison across datasets using full training. Values show test accuracy as mean ± standard deviation. – indicates no data available. Subscript numbers in parentheses indicate sample size.

Model	PAPILA	RFMiD Binary	RFMiD Multi
BE-DINORET	$0.491 \pm 0.064_{(3)}$	$0.852 \pm 0.013_{(3)}$	$0.487 \pm 0.047_{(3)}$
DINORET	$0.495 \pm 0.021_{(3)}$	$0.875 \pm 0.009_{(3)}$	$0.389 \pm 0.020_{(3)}$
DINOv2 ViT-B/14	$0.563 \pm 0.021_{(3)}$	$0.843 \pm 0.014_{(3)}$	$0.466 \pm 0.033_{(3)}$
DINOv2 ViT-B/14 REG	$0.495 \pm 0.077_{(3)}$	$0.855 \pm 0.018_{(3)}$	$0.452 \pm 0.014_{(3)}$
DINOv2 ViT-G/14	$0.428 \pm 0.195_{(3)}$	$0.867 \pm 0.013_{(3)}$	$0.506 \pm 0.043_{(3)}$
DINOv2 ViT-G/14 REG	$0.446 \pm 0.036_{(3)}$	$0.875 \pm 0.009_{(3)}$	$0.500 \pm 0.058_{(3)}$
DINOv2 ViT-S/14	$0.509 \pm 0.096_{(3)}$	$0.868 \pm 0.021_{(3)}$	$0.479 \pm 0.072_{(3)}$
DINOv2 ViT-S/14 REG	$0.532 \pm 0.055_{(3)}$	$0.857 \pm 0.021_{(3)}$	$0.451 \pm 0.071_{(3)}$
LoRA-DINORET	$0.586 \pm 0.067_{(3)}$	$0.854 \pm 0.014_{(3)}$	$0.533 \pm 0.019_{(3)}$
Prov-GigaPath	$0.486 \pm 0.075_{(3)}$	$0.858 \pm 0.006_{(3)}$	$0.513 \pm 0.064_{(3)}$
RadDINO	$0.541 \pm 0.106_{(3)}$	$0.830 \pm 0.001_{(3)}$	$0.361 \pm 0.033_{(3)}$
ResNet-50 (ImageNet)	$0.527 \pm 0.049_{(3)}$	$0.852 \pm 0.010_{(3)}$	$0.460 \pm 0.040_{(3)}$
ResNet-50 (Random)	$0.428 \pm 0.077_{(3)}$	$0.812 \pm 0.025_{(3)}$	$0.209 \pm 0.022_{(3)}$
RET-CLIP	$0.302 \pm 0.055_{(3)}$	$0.597 \pm 0.054_{(3)}$	$0.153 \pm 0.033_{(3)}$
RETFound DINOv2 (MEH)	$0.568 \pm 0.036_{(3)}$	$0.854 \pm 0.008_{(3)}$	$0.518 \pm 0.041_{(3)}$
RETFound DINOv2 (Shanghai)	$0.586 \pm 0.047_{(3)}$	$0.842 \pm 0.009_{(3)}$	$0.548 \pm 0.028_{(3)}$
RETFound MAE (MEH)	$0.428 \pm 0.068_{(3)}$	$0.850 \pm 0.010_{(3)}$	$0.468 \pm 0.033_{(3)}$
RETFound MAE (Nature CFP)	$0.374 \pm 0.034_{(3)}$	$0.857 \pm 0.022_{(3)}$	$0.414 \pm 0.092_{(3)}$
RETFound MAE (Nature OCT)	$0.324 \pm 0.014_{(3)}$	$0.755 \pm 0.029_{(3)}$	$0.177 \pm 0.042_{(3)}$
RETFound MAE (Shanghai)	$0.459 \pm 0.068_{(3)}$	$0.853 \pm 0.002_{(3)}$	$0.454 \pm 0.058_{(3)}$
RETFound Green	$0.541 \pm 0.106_{(3)}$	$0.871 \pm 0.007_{(3)}$	$0.464 \pm 0.065_{(3)}$
RETIZero	$0.581 \pm 0.118_{(3)}$	$0.859 \pm 0.023_{(3)}$	$0.577 \pm 0.044_{(3)}$
SigLIP	$0.617 \pm 0.096_{(3)}$	$0.852 \pm 0.007_{(3)}$	$0.459 \pm 0.045_{(3)}$
SigLIP2	$0.541 \pm 0.089_{(3)}$	$0.853 \pm 0.002_{(3)}$	$0.462 \pm 0.054_{(3)}$
URFound	$0.464 \pm 0.055_{(3)}$	$0.879 \pm 0.015_{(3)}$	$0.563 \pm 0.103_{(3)}$
ViLRef	$0.302 \pm 0.055_{(3)}$	$0.597 \pm 0.054_{(3)}$	$0.153 \pm 0.033_{(3)}$
Virchow	$0.505 \pm 0.008_{(3)}$	$0.852 \pm 0.019_{(3)}$	$0.475 \pm 0.014_{(3)}$
Virchow2	$0.509 \pm 0.074_{(3)}$	$0.854 \pm 0.013_{(3)}$	$0.464 \pm 0.067_{(3)}$
VisionFM Fundus	$0.532 \pm 0.051_{(3)}$	$0.845 \pm 0.021_{(3)}$	$0.492 \pm 0.041_{(3)}$
VisionFM OCT	$0.392 \pm 0.082_{(3)}$	$0.816 \pm 0.020_{(3)}$	$0.291 \pm 0.003_{(3)}$
ViT-B/16 CLIP	$0.559 \pm 0.056_{(3)}$	$0.859 \pm 0.013_{(3)}$	$0.505 \pm 0.036_{(3)}$
ViT-B/32 CLIP	$0.532 \pm 0.028_{(3)}$	$0.861 \pm 0.009_{(3)}$	$0.463 \pm 0.045_{(3)}$
ViT-L/14 CLIP	$0.586 \pm 0.008_{(3)}$	$0.874 \pm 0.008_{(3)}$	$0.491 \pm 0.090_{(3)}$
ViT-B (ImageNet)	$0.577 \pm 0.021_{(3)}$	$0.850 \pm 0.015_{(3)}$	$0.513 \pm 0.030_{(3)}$

Table A.11: Performance comparison across datasets using full training. Values show test accuracy as mean ± standard deviation. – indicates no data available. Subscript numbers in parentheses indicate sample size.

Appendix A.5.2. Unbalanced Training - Unbalanced Test

**Model**	**AIREADI Grouped Study Group CFP**	**AIREADI Grouped Study Group OCT**	**AIREADI Study Group CFP**	**AIREADI Study Group OCT**
BE-DINORET	$0.573 \pm 0.016_{(3)}$	$0.545 \pm 0.018_{(3)}$	$0.314 \pm 0.006_{(3)}$	$0.289 \pm 0.012_{(3)}$
DINORET	$0.562 \pm 0.024_{(3)}$	$0.589 \pm 0.031_{(3)}$	$0.323 \pm 0.015_{(3)}$	$0.292 \pm 0.042_{(3)}$
DINOv2 ViT-B/14	$0.571 \pm 0.006_{(3)}$	$0.528 \pm 0.015_{(3)}$	$0.321 \pm 0.030_{(3)}$	$0.288 \pm 0.013_{(3)}$
DINOv2 ViT-B/14 REG	$0.584 \pm 0.012_{(3)}$	$0.549 \pm 0.005_{(3)}$	$0.308 \pm 0.010_{(3)}$	$0.300 \pm 0.030_{(3)}$
DINOv2 ViT-G/14	$0.555 \pm 0.009_{(3)}$	$0.570 \pm 0.016_{(3)}$	$0.277 \pm 0.040_{(3)}$	$0.305 \pm 0.028_{(3)}$
DINOv2 ViT-G/14 REG	$0.583 \pm 0.007_{(3)}$	$0.522 \pm 0.025_{(3)}$	$0.308 \pm 0.057_{(3)}$	$0.298 \pm 0.029_{(3)}$
DINOv2 ViT-S/14	$0.586 \pm 0.010_{(3)}$	$0.575 \pm 0.026_{(3)}$	$0.307 \pm 0.037_{(3)}$	$0.293 \pm 0.021_{(3)}$
DINOv2 ViT-S/14 REG	$0.562 \pm 0.012_{(3)}$	$0.544 \pm 0.022_{(3)}$	$0.321 \pm 0.041_{(3)}$	$0.272 \pm 0.023_{(3)}$
LoRA-DINORET	$0.590 \pm 0.017_{(3)}$	$0.570 \pm 0.034_{(3)}$	$0.293 \pm 0.004_{(3)}$	$0.259 \pm 0.005_{(3)}$
Prov-GigaPath	$0.570 \pm 0.004_{(3)}$	$0.547 \pm 0.022_{(3)}$	$0.305 \pm 0.038_{(3)}$	$0.300 \pm 0.016_{(3)}$
RadDINO	$0.586 \pm 0.024_{(3)}$	$0.553 \pm 0.024_{(3)}$	$0.322 \pm 0.033_{(3)}$	$0.298 \pm 0.041_{(3)}$
ResNet-50 (ImageNet)	$0.572 \pm 0.010_{(3)}$	$0.534 \pm 0.032_{(3)}$	$0.315 \pm 0.057_{(3)}$	$0.303 \pm 0.006_{(3)}$
ResNet-50 (Random)	$0.537 \pm 0.024_{(3)}$	$0.517 \pm 0.037_{(3)}$	$0.269 \pm 0.014_{(3)}$	$0.267 \pm 0.026_{(3)}$
RET-CLIP	$0.516 \pm 0.030_{(3)}$	$0.468 \pm 0.034_{(3)}$	$0.249 \pm 0.015_{(3)}$	$0.287 \pm 0.033_{(3)}$
RETFound DINOv2 (MEH)	$0.570 \pm 0.015_{(3)}$	$0.545 \pm 0.033_{(3)}$	$0.323 \pm 0.027_{(3)}$	$0.282 \pm 0.016_{(3)}$
RETFound DINOv2 (Shanghai)	$0.568 \pm 0.013_{(3)}$	$0.547 \pm 0.014_{(3)}$	$0.303 \pm 0.026_{(3)}$	$0.284 \pm 0.022_{(3)}$
RETFound MAE (MEH)	$0.581 \pm 0.009_{(3)}$	$0.530 \pm 0.010_{(3)}$	$0.322 \pm 0.050_{(3)}$	$0.307 \pm 0.056_{(3)}$
RETFound MAE (Nature CFP)	$0.590 \pm 0.031_{(3)}$	$0.557 \pm 0.034_{(3)}$	$0.303 \pm 0.020_{(3)}$	$0.261 \pm 0.053_{(3)}$
RETFound MAE (Nature OCT)	$0.546 \pm 0.011_{(3)}$	$0.556 \pm 0.029_{(3)}$	$0.272 \pm 0.023_{(3)}$	$0.308 \pm 0.037_{(3)}$
RETFound MAE (Shanghai)	$0.589 \pm 0.014_{(3)}$	$0.548 \pm 0.008_{(3)}$	$0.307 \pm 0.023_{(3)}$	$0.303 \pm 0.012_{(3)}$
RETFound Green	$0.554 \pm 0.013_{(3)}$	$0.516 \pm 0.038_{(3)}$	$0.279 \pm 0.056_{(3)}$	$0.279 \pm 0.010_{(3)}$
RETIZero	$0.585 \pm 0.019_{(3)}$	$0.545 \pm 0.025_{(3)}$	$0.334 \pm 0.007_{(3)}$	$0.288 \pm 0.019_{(3)}$
SigLIP	$0.558 \pm 0.021_{(3)}$	$0.566 \pm 0.010_{(3)}$	$0.281 \pm 0.051_{(3)}$	$0.284 \pm 0.042_{(3)}$
SigLIP2	$0.560 \pm 0.008_{(3)}$	$0.465 \pm 0.021_{(3)}$	$0.330 \pm 0.019_{(3)}$	$0.277 \pm 0.034_{(3)}$
URFound	$0.590 \pm 0.011_{(3)}$	$0.576 \pm 0.015_{(3)}$	$0.328 \pm 0.014_{(3)}$	$0.301 \pm 0.039_{(3)}$
ViLRef	$0.516 \pm 0.030_{(3)}$	$0.468 \pm 0.034_{(3)}$	$0.249 \pm 0.015_{(3)}$	$0.287 \pm 0.033_{(3)}$
Virchow	$0.579 \pm 0.019_{(3)}$	$0.571 \pm 0.025_{(3)}$	$0.333 \pm 0.026_{(3)}$	$0.311 \pm 0.023_{(3)}$
Virchow2	$0.559 \pm 0.015_{(3)}$	$0.544 \pm 0.037_{(3)}$	$0.292 \pm 0.035_{(3)}$	$0.312 \pm 0.006_{(3)}$
VisionFM Fundus	$0.585 \pm 0.010_{(3)}$	$0.543 \pm 0.026_{(3)}$	$0.292 \pm 0.024_{(3)}$	$0.276 \pm 0.013_{(3)}$
VisionFM OCT	$0.574 \pm 0.018_{(3)}$	$0.538 \pm 0.037_{(3)}$	$0.323 \pm 0.017_{(3)}$	$0.256 \pm 0.019_{(3)}$
ViT-B/16 CLIP	$0.568 \pm 0.017_{(3)}$	$0.527 \pm 0.032_{(3)}$	$0.315 \pm 0.036_{(3)}$	$0.293 \pm 0.012_{(3)}$
ViT-B/32 CLIP	$0.587 \pm 0.019_{(3)}$	$0.539 \pm 0.017_{(3)}$	$0.312 \pm 0.018_{(3)}$	$0.273 \pm 0.012_{(3)}$
ViT-L/14 CLIP	$0.580 \pm 0.023_{(3)}$	$0.580 \pm 0.016_{(3)}$	$0.294 \pm 0.015_{(3)}$	$0.308 \pm 0.048_{(3)}$
ViT-B (ImageNet)	$0.572 \pm 0.030_{(3)}$	$0.572 \pm 0.010_{(3)}$	$0.321 \pm 0.023_{(3)}$	$0.280 \pm 0.011_{(3)}$

Table A.12: Performance comparison across datasets using full training. Values show test accuracy as mean ± standard deviation. – indicates no data available. Subscript numbers in parentheses indicate sample size.

Model	APTOS	BRSET DR	BRSET HT	DR-TID
BE-DINORET	$0.762 \pm 0.020_{(3)}$	$0.672 \pm 0.044_{(3)}$	$0.699 \pm 0.013_{(3)}$	$0.432 \pm 0.042_{(3)}$
DINORET	$0.733 \pm 0.028_{(3)}$	$0.485 \pm 0.305_{(3)}$	$0.705 \pm 0.013_{(3)}$	$0.438 \pm 0.026_{(3)}$
DINOv2 ViT-B/14	$0.773 \pm 0.030_{(3)}$	$0.660 \pm 0.098_{(3)}$	$0.707 \pm 0.008_{(3)}$	$0.459 \pm 0.024_{(3)}$
DINOv2 ViT-B/14 REG	$0.756 \pm 0.019_{(3)}$	$0.666 \pm 0.024_{(3)}$	$0.708 \pm 0.008_{(3)}$	$0.462 \pm 0.057_{(3)}$
DINOv2 ViT-G/14	$0.743 \pm 0.012_{(3)}$	$0.722 \pm 0.113_{(3)}$	$0.730 \pm 0.010_{(3)}$	$0.426 \pm 0.077_{(3)}$
DINOv2 ViT-G/14 REG	$0.762 \pm 0.007_{(3)}$	$0.716 \pm 0.071_{(3)}$	$0.704 \pm 0.001_{(3)}$	$0.401 \pm 0.071_{(3)}$
DINOv2 ViT-S/14	$0.730 \pm 0.010_{(3)}$	$0.672 \pm 0.033_{(3)}$	$0.702 \pm 0.023_{(3)}$	$0.448 \pm 0.091_{(3)}$
DINOv2 ViT-S/14 REG	$0.755 \pm 0.019_{(3)}$	$0.657 \pm 0.153_{(3)}$	$0.700 \pm 0.006_{(3)}$	$0.379 \pm 0.073_{(3)}$
LoRA-DINORET	$0.767 \pm 0.015_{(3)}$	$0.589 \pm 0.227_{(3)}$	$0.715 \pm 0.020_{(3)}$	$0.498 \pm 0.013_{(3)}$
Prov-GigaPath	$0.755 \pm 0.031_{(3)}$	$0.758 \pm 0.016_{(3)}$	$0.701 \pm 0.011_{(3)}$	$0.416 \pm 0.056_{(3)}$
RadDINO	$0.722 \pm 0.021_{(3)}$	$0.585 \pm 0.052_{(3)}$	$0.694 \pm 0.012_{(3)}$	$0.346 \pm 0.030_{(3)}$
ResNet-50 (ImageNet)	$0.736 \pm 0.024_{(3)}$	$0.644 \pm 0.013_{(3)}$	$0.709 \pm 0.006_{(3)}$	$0.332 \pm 0.022_{(3)}$
ResNet-50 (Random)	$0.636 \pm 0.060_{(3)}$	$0.321 \pm 0.288_{(3)}$	$0.622 \pm 0.015_{(3)}$	$0.190 \pm 0.039_{(3)}$
RET-CLIP	$0.378 \pm 0.038_{(3)}$	$0.259 \pm 0.220_{(3)}$	$0.652 \pm 0.005_{(3)}$	$0.193 \pm 0.061_{(3)}$
RETFound DINOv2 (MEH)	$0.775 \pm 0.019_{(3)}$	$0.688 \pm 0.005_{(3)}$	$0.716 \pm 0.013_{(3)}$	$0.393 \pm 0.022_{(3)}$
RETFound DINOv2 (Shanghai)	$0.748 \pm 0.008_{(3)}$	$0.745 \pm 0.042_{(3)}$	$0.735 \pm 0.015_{(3)}$	$0.348 \pm 0.013_{(3)}$
RETFound MAE (MEH)	$0.757 \pm 0.035_{(3)}$	$0.613 \pm 0.132_{(3)}$	$0.685 \pm 0.010_{(3)}$	$0.398 \pm 0.063_{(3)}$
RETFound MAE (Nature CFP)	$0.765 \pm 0.067_{(3)}$	$0.642 \pm 0.113_{(3)}$	$0.696 \pm 0.034_{(3)}$	$0.406 \pm 0.041_{(3)}$
RETFound MAE (Nature OCT)	$0.614 \pm 0.026_{(3)}$	$0.280 \pm 0.029_{(3)}$	$0.657 \pm 0.008_{(3)}$	$0.199 \pm 0.029_{(3)}$
RETFound MAE (Shanghai)	$0.776 \pm 0.014_{(3)}$	$0.754 \pm 0.163_{(3)}$	$0.713 \pm 0.015_{(3)}$	$0.489 \pm 0.073_{(3)}$
RETFound Green	$0.719 \pm 0.037_{(3)}$	$0.627 \pm 0.188_{(3)}$	$0.712 \pm 0.009_{(3)}$	$0.418 \pm 0.027_{(3)}$
RETIZero	$0.804 \pm 0.029_{(3)}$	$0.746 \pm 0.096_{(3)}$	$0.710 \pm 0.003_{(3)}$	$0.522 \pm 0.039_{(3)}$
SigLIP	$0.780 \pm 0.028_{(3)}$	$0.539 \pm 0.166_{(3)}$	$0.702 \pm 0.018_{(3)}$	$0.402 \pm 0.064_{(3)}$
SigLIP2	$0.756 \pm 0.021_{(3)}$	$0.587 \pm 0.026_{(3)}$	$0.698 \pm 0.008_{(3)}$	$0.396 \pm 0.037_{(3)}$
URFound	$0.786 \pm 0.028_{(3)}$	$0.788 \pm 0.064_{(3)}$	$0.703 \pm 0.012_{(3)}$	$0.479 \pm 0.028_{(3)}$
ViLRef	$0.378 \pm 0.038_{(3)}$	$0.259 \pm 0.220_{(3)}$	$0.652 \pm 0.005_{(3)}$	$0.193 \pm 0.061_{(3)}$
Virchow	$0.755 \pm 0.013_{(3)}$	$0.616 \pm 0.200_{(3)}$	$0.669 \pm 0.015_{(3)}$	$0.376 \pm 0.062_{(3)}$
Virchow2	$0.774 \pm 0.021_{(3)}$	$0.815 \pm 0.022_{(3)}$	$0.704 \pm 0.018_{(3)}$	$0.412 \pm 0.053_{(3)}$
VisionFM Fundus	$0.698 \pm 0.009_{(3)}$	$0.619 \pm 0.044_{(3)}$	$0.712 \pm 0.010_{(3)}$	$0.355 \pm 0.038_{(3)}$
VisionFM OCT	$0.646 \pm 0.039_{(3)}$	$0.433 \pm 0.114_{(3)}$	$0.673 \pm 0.010_{(3)}$	$0.323 \pm 0.052_{(3)}$
ViT-B/16 CLIP	$0.764 \pm 0.028_{(3)}$	$0.604 \pm 0.036_{(3)}$	$0.706 \pm 0.024_{(3)}$	$0.445 \pm 0.022_{(3)}$
ViT-B/32 CLIP	$0.744 \pm 0.010_{(3)}$	$0.578 \pm 0.118_{(3)}$	$0.704 \pm 0.009_{(3)}$	$0.416 \pm 0.007_{(3)}$
ViT-L/14 CLIP	$0.781 \pm 0.029_{(3)}$	$0.674 \pm 0.070_{(3)}$	$0.711 \pm 0.038_{(3)}$	$0.428 \pm 0.071_{(3)}$
ViT-B (ImageNet)	$0.753 \pm 0.012_{(3)}$	$0.605 \pm 0.104_{(3)}$	$0.695 \pm 0.035_{(3)}$	$0.389 \pm 0.084_{(3)}$

Table A.13: Performance comparison across datasets using full training. Values show test accuracy as mean ± standard deviation. – indicates no data available. Subscript numbers in parentheses indicate sample size.

Model	EyePACS	EyePACS Balanced	FIMD	GRAPE MD
BE-DINORET	$0.499 \pm 0.018_{(3)}$	$0.505 \pm 0.018_{(3)}$	$0.455 \pm 0.091_{(3)}$	$0.592 \pm 0.088_{(3)}$
DINORET	$0.519 \pm 0.150_{(3)}$	$0.552 \pm 0.011_{(3)}$	$0.576 \pm 0.262_{(3)}$	$0.720 \pm 0.092_{(3)}$
DINOv2 ViT-B/14	$0.497 \pm 0.018_{(3)}$	$0.500 \pm 0.008_{(3)}$	$0.455 \pm 0.157_{(3)}$	$0.688 \pm 0.063_{(3)}$
DINOv2 ViT-B/14 REG	$0.517 \pm 0.024_{(3)}$	$0.544 \pm 0.022_{(3)}$	$0.515 \pm 0.189_{(3)}$	$0.588 \pm 0.089_{(3)}$
DINOv2 ViT-G/14	$0.513 \pm 0.115_{(3)}$	$0.567 \pm 0.022_{(3)}$	$0.667 \pm 0.189_{(3)}$	$0.369 \pm 0.070_{(3)}$
DINOv2 ViT-G/14 REG	$0.506 \pm 0.028_{(3)}$	$0.510 \pm 0.021_{(3)}$	$0.515 \pm 0.052_{(3)}$	$0.396 \pm 0.222_{(3)}$
DINOv2 ViT-S/14	$0.536 \pm 0.095_{(3)}$	$0.543 \pm 0.010_{(3)}$	$0.667 \pm 0.139_{(3)}$	$0.532 \pm 0.074_{(3)}$
DINOv2 ViT-S/14 REG	$0.548 \pm 0.030_{(3)}$	$0.546 \pm 0.020_{(3)}$	$0.545_{(3)}$	$0.459 \pm 0.015_{(3)}$
LoRA-DINORET	$0.438 \pm 0.054_{(3)}$	$0.555 \pm 0.020_{(3)}$	$0.636 \pm 0.091_{(3)}$	$0.426 \pm 0.225_{(3)}$
Prov-GigaPath	$0.489 \pm 0.011_{(3)}$	$0.494 \pm 0.014_{(3)}$	$0.515 \pm 0.052_{(3)}$	$0.508 \pm 0.071_{(3)}$
RadDINO	$0.486 \pm 0.123_{(3)}$	$0.483 \pm 0.018_{(3)}$	$0.576 \pm 0.105_{(3)}$	$0.558 \pm 0.063_{(3)}$
ResNet-50 (ImageNet)	$0.464 \pm 0.033_{(3)}$	$0.494 \pm 0.013_{(3)}$	$0.606 \pm 0.189_{(3)}$	$0.614 \pm 0.035_{(3)}$
ResNet-50 (Random)	$0.248 \pm 0.045_{(3)}$	$0.266 \pm 0.005_{(3)}$	$0.515 \pm 0.052_{(3)}$	$0.562 \pm 0.294_{(3)}$
RET-CLIP	$0.244 \pm 0.023_{(3)}$	$0.220 \pm 0.016_{(3)}$	$0.606 \pm 0.105_{(3)}$	$0.406 \pm 0.228_{(3)}$
RETFound DINOv2 (MEH)	$0.531 \pm 0.009_{(3)}$	$0.540 \pm 0.008_{(3)}$	$0.606 \pm 0.189_{(3)}$	$0.530 \pm 0.148_{(3)}$
RETFound DINOv2 (Shanghai)	$0.502 \pm 0.042_{(3)}$	$0.553 \pm 0.007_{(3)}$	$0.667 \pm 0.189_{(3)}$	$0.484 \pm 0.138_{(3)}$
RETFound MAE (MEH)	$0.435 \pm 0.076_{(3)}$	$0.510 \pm 0.023_{(3)}$	$0.545 \pm 0.157_{(3)}$	$0.525 \pm 0.225_{(3)}$
RETFound MAE (Nature CFP)	$0.633 \pm 0.044_{(3)}$	$0.492 \pm 0.001_{(3)}$	$0.545 \pm 0.091_{(3)}$	$0.331 \pm 0.264_{(3)}$
RETFound MAE (Nature OCT)	$0.254 \pm 0.032_{(3)}$	$0.261 \pm 0.008_{(3)}$	$0.515 \pm 0.229_{(3)}$	$0.564 \pm 0.109_{(3)}$
RETFound MAE (Shanghai)	$0.560 \pm 0.089_{(3)}$	$0.520 \pm 0.007_{(3)}$	$0.485 \pm 0.105_{(3)}$	$0.598 \pm 0.200_{(3)}$
RETFound Green	$0.518 \pm 0.038_{(3)}$	$0.523 \pm 0.018_{(3)}$	$0.576 \pm 0.189_{(3)}$	$0.606 \pm 0.049_{(3)}$
RETIZero	$0.556 \pm 0.021_{(3)}$	$0.542 \pm 0.001_{(3)}$	$0.576 \pm 0.139_{(3)}$	$0.365 \pm 0.213_{(3)}$
SigLIP	$0.399 \pm 0.128_{(3)}$	$0.532 \pm 0.008_{(3)}$	$0.576 \pm 0.139_{(3)}$	$0.548 \pm 0.131_{(3)}$
SigLIP2	$0.463 \pm 0.024_{(3)}$	$0.491 \pm 0.009_{(3)}$	$0.485 \pm 0.052_{(3)}$	$0.574 \pm 0.044_{(3)}$
URFound	$0.510 \pm 0.073_{(3)}$	$0.553 \pm 0.007_{(3)}$	$0.515 \pm 0.105_{(3)}$	$0.639 \pm 0.251_{(3)}$
ViLRef	$0.244 \pm 0.023_{(3)}$	$0.220 \pm 0.016_{(3)}$	$0.606 \pm 0.105_{(3)}$	$0.406 \pm 0.228_{(3)}$
Virchow	$0.542 \pm 0.150_{(3)}$	$0.557 \pm 0.005_{(3)}$	$0.576 \pm 0.139_{(3)}$	$0.698 \pm 0.047_{(3)}$
Virchow2	$0.552 \pm 0.029_{(3)}$	$0.541 \pm 0.007_{(3)}$	$0.485 \pm 0.052_{(3)}$	$0.459 \pm 0.250_{(3)}$
VisionFM Fundus	$0.496 \pm 0.017_{(3)}$	$0.512 \pm 0.021_{(3)}$	$0.545 \pm 0.091_{(3)}$	$0.447 \pm 0.217_{(3)}$
VisionFM OCT	$0.384 \pm 0.152_{(3)}$	$0.433 \pm 0.016_{(3)}$	$0.485 \pm 0.052_{(3)}$	$0.706 \pm 0.106_{(3)}$
ViT-B/16 CLIP	$0.500 \pm 0.038_{(3)}$	$0.541 \pm 0.017_{(3)}$	$0.485 \pm 0.105_{(3)}$	$0.512 \pm 0.292_{(3)}$
ViT-B/32 CLIP	$0.499 \pm 0.043_{(3)}$	$0.498 \pm 0.013_{(3)}$	$0.485 \pm 0.139_{(3)}$	$0.440 \pm 0.231_{(3)}$
ViT-L/14 CLIP	$0.620 \pm 0.075_{(3)}$	$0.543 \pm 0.024_{(3)}$	$0.576 \pm 0.105_{(3)}$	$0.598 \pm 0.204_{(3)}$
ViT-B (ImageNet)	$0.509 \pm 0.085_{(3)}$	$0.517 \pm 0.003_{(3)}$	$0.606 \pm 0.105_{(3)}$	$0.638 \pm 0.120_{(3)}$

Table A.14: Performance comparison across datasets using full training. Values show test accuracy as mean ± standard deviation. – indicates no data available. Subscript numbers in parentheses indicate sample size.

Model	GRAPE PLR2	GRAPE PLR3	Harvard FairFedMed Glaucoma	Harvard FairGenMed Glaucoma
BE-DINORET	$0.486 \pm 0.128_{(3)}$	$0.472 \pm 0.124_{(3)}$	$0.740 \pm 0.004_{(3)}$	$0.708 \pm 0.016_{(3)}$
DINORET	$0.588 \pm 0.156_{(3)}$	$0.537 \pm 0.063_{(3)}$	$0.764 \pm 0.002_{(3)}$	$0.744 \pm 0.004_{(3)}$
DINOv2 ViT-B/14	$0.491 \pm 0.206_{(3)}$	$0.504 \pm 0.025_{(3)}$	$0.729 \pm 0.005_{(3)}$	$0.708 \pm 0.002_{(3)}$
DINOv2 ViT-B/14 REG	$0.365 \pm 0.151_{(3)}$	$0.352 \pm 0.041_{(3)}$	$0.758 \pm 0.004_{(3)}$	$0.740 \pm 0.004_{(3)}$
DINOv2 ViT-G/14	$0.496 \pm 0.229_{(3)}$	$0.545 \pm 0.145_{(3)}$	$0.767 \pm 0.001_{(3)}$	$0.742 \pm 0.014_{(3)}$
DINOv2 ViT-G/14 REG	$0.427 \pm 0.281_{(3)}$	$0.585 \pm 0.327_{(3)}$	$0.747 \pm 0.002_{(3)}$	$0.716 \pm 0.012_{(3)}$
DINOv2 ViT-S/14	$0.505 \pm 0.181_{(3)}$	$0.569 \pm 0.099_{(3)}$	$0.763 \pm 0.001_{(3)}$	$0.746 \pm 0.010_{(3)}$
DINOv2 ViT-S/14 REG	$0.636 \pm 0.073_{(3)}$	$0.601 \pm 0.192_{(3)}$	$0.769 \pm 0.004_{(3)}$	$0.746 \pm 0.009_{(3)}$
LoRA-DINORET	$0.294 \pm 0.097_{(3)}$	$0.656 \pm 0.121_{(3)}$	$0.770 \pm 0.003_{(3)}$	$0.751 \pm 0.008_{(3)}$
Prov-GigaPath	$0.521 \pm 0.155_{(3)}$	$0.537 \pm 0.103_{(3)}$	$0.740 \pm 0.005_{(3)}$	$0.720 \pm 0.008_{(3)}$
RadDINO	$0.534 \pm 0.088_{(3)}$	$0.560 \pm 0.032_{(3)}$	$0.761 \pm 0.004_{(3)}$	$0.721 \pm 0.002_{(3)}$
ResNet-50 (ImageNet)	$0.620 \pm 0.116_{(3)}$	$0.495 \pm 0.128_{(3)}$	$0.750 \pm 0.006_{(3)}$	$0.720 \pm 0.015_{(3)}$
ResNet-50 (Random)	$0.552 \pm 0.035_{(3)}$	$0.479 \pm 0.232_{(3)}$	$0.745 \pm 0.007_{(3)}$	$0.683 \pm 0.003_{(3)}$
RET-CLIP	$0.652 \pm 0.161_{(3)}$	$0.528 \pm 0.048_{(3)}$	$0.606 \pm 0.020_{(3)}$	$0.536 \pm 0.006_{(3)}$
RETFound DINOv2 (MEH)	$0.428 \pm 0.184_{(3)}$	$0.608 \pm 0.161_{(3)}$	$0.699 \pm 0.012_{(3)}$	$0.720 \pm 0.001_{(3)}$
RETFound DINOv2 (Shanghai)	$0.586 \pm 0.088_{(3)}$	$0.496 \pm 0.079_{(3)}$	$0.767 \pm 0.006_{(3)}$	$0.739 \pm 0.001_{(3)}$
RETFound MAE (MEH)	$0.488 \pm 0.126_{(3)}$	$0.696 \pm 0.016_{(3)}$	$0.758 \pm 0.005_{(3)}$	$0.733 \pm 0.006_{(3)}$
RETFound MAE (Nature CFP)	$0.603 \pm 0.287_{(3)}$	$0.665 \pm 0.226_{(3)}$	$0.768 \pm 0.005_{(3)}$	$0.727 \pm 0.007_{(3)}$
RETFound MAE (Nature OCT)	$0.544 \pm 0.101_{(3)}$	$0.360 \pm 0.095_{(3)}$	$0.688 \pm 0.008_{(3)}$	$0.646 \pm 0.008_{(3)}$
RETFound MAE (Shanghai)	$0.564 \pm 0.193_{(3)}$	$0.543 \pm 0.277_{(3)}$	$0.764 \pm 0.009_{(3)}$	$0.724 \pm 0.005_{(3)}$
RETFound Green	$0.411 \pm 0.205_{(3)}$	$0.517 \pm 0.349_{(3)}$	$0.752 \pm 0.004_{(3)}$	$0.728 \pm 0.016_{(3)}$
RETIZero	$0.341 \pm 0.090_{(3)}$	$0.490 \pm 0.268_{(3)}$	$0.747 \pm 0.006_{(3)}$	$0.726 \pm 0.004_{(3)}$
SigLIP	$0.395 \pm 0.125_{(3)}$	$0.577 \pm 0.173_{(3)}$	$0.770 \pm 0.005_{(3)}$	$0.737 \pm 0.000_{(3)}$
SigLIP2	$0.447 \pm 0.259_{(3)}$	$0.641 \pm 0.092_{(3)}$	$0.727 \pm 0.008_{(3)}$	$0.703 \pm 0.008_{(3)}$
URFound	$0.499 \pm 0.278_{(3)}$	$0.600 \pm 0.038_{(3)}$	$0.773 \pm 0.007_{(3)}$	$0.749 \pm 0.005_{(3)}$
ViLRef	$0.652 \pm 0.161_{(3)}$	$0.528 \pm 0.048_{(3)}$	$0.606 \pm 0.020_{(3)}$	$0.536 \pm 0.006_{(3)}$
Virchow	$0.444 \pm 0.122_{(3)}$	$0.617 \pm 0.082_{(3)}$	$0.762 \pm 0.006_{(3)}$	$0.745 \pm 0.007_{(3)}$
Virchow2	$0.377 \pm 0.154_{(3)}$	$0.505 \pm 0.158_{(3)}$	$0.762 \pm 0.002_{(3)}$	$0.742 \pm 0.012_{(3)}$
VisionFM Fundus	$0.534 \pm 0.073_{(3)}$	$0.521 \pm 0.232_{(3)}$	$0.735 \pm 0.006_{(3)}$	$0.709 \pm 0.007_{(3)}$
VisionFM OCT	$0.663 \pm 0.192_{(3)}$	$0.671 \pm 0.207_{(3)}$	$0.780 \pm 0.004_{(3)}$	$0.723 \pm 0.011_{(3)}$
ViT-B/16 CLIP	$0.617 \pm 0.251_{(3)}$	$0.490 \pm 0.281_{(3)}$	$0.764 \pm 0.010_{(3)}$	$0.723 \pm 0.003_{(3)}$
ViT-B/32 CLIP	$0.468 \pm 0.124_{(3)}$	$0.529 \pm 0.252_{(3)}$	$0.755 \pm 0.012_{(3)}$	$0.724 \pm 0.005_{(3)}$
ViT-L/14 CLIP	$0.623 \pm 0.185_{(3)}$	$0.688 \pm 0.085_{(3)}$	$0.771 \pm 0.000_{(3)}$	$0.747 \pm 0.008_{(3)}$
ViT-B (ImageNet)	$0.375 \pm 0.135_{(3)}$	$0.529 \pm 0.158_{(3)}$	$0.758 \pm 0.008_{(3)}$	$0.727 \pm 0.010_{(3)}$

Table A.15: Performance comparison across datasets using full training. Values show test accuracy as mean ± standard deviation. – indicates no data available. Subscript numbers in parentheses indicate sample size.

Model	Harvard FairGenMed Vision Loss	Harvard FairVision AMD	Harvard FairVision DR	Harvard FairVision Glaucoma
BE-DINORET	$0.486 \pm 0.012_{(3)}$	$0.674 \pm 0.032_{(3)}$	$0.537 \pm 0.039_{(3)}$	$0.488 \pm 0.021_{(3)}$
DINORET	$0.451 \pm 0.035_{(3)}$	$0.704 \pm 0.073_{(3)}$	$0.519 \pm 0.263_{(3)}$	$0.501 \pm 0.014_{(3)}$
DINOv2 ViT-B/14	$0.485 \pm 0.009_{(3)}$	$0.668 \pm 0.025_{(3)}$	$0.517 \pm 0.075_{(3)}$	$0.490 \pm 0.013_{(3)}$
DINOv2 ViT-B/14 REG	$0.501 \pm 0.014_{(3)}$	$0.677 \pm 0.029_{(3)}$	$0.473 \pm 0.045_{(3)}$	$0.493 \pm 0.001_{(3)}$
DINOv2 ViT-G/14	$0.487 \pm 0.039_{(3)}$	$0.674 \pm 0.029_{(3)}$	$0.452 \pm 0.154_{(3)}$	$0.507 \pm 0.024_{(3)}$
DINOv2 ViT-G/14 REG	$0.496 \pm 0.031_{(3)}$	$0.687 \pm 0.036_{(3)}$	$0.536 \pm 0.106_{(3)}$	$0.493 \pm 0.003_{(3)}$
DINOv2 ViT-S/14	$0.483 \pm 0.061_{(3)}$	$0.672 \pm 0.055_{(3)}$	$0.364 \pm 0.221_{(3)}$	$0.495 \pm 0.014_{(3)}$
DINOv2 ViT-S/14 REG	$0.500 \pm 0.045_{(3)}$	$0.712 \pm 0.029_{(3)}$	$0.537 \pm 0.088_{(3)}$	$0.508 \pm 0.006_{(3)}$
LoRA-DINORET	$0.525 \pm 0.029_{(3)}$	$0.705 \pm 0.080_{(3)}$	$0.412 \pm 0.256_{(3)}$	$0.504 \pm 0.017_{(3)}$
Prov-GigaPath	$0.487 \pm 0.016_{(3)}$	$0.674 \pm 0.025_{(3)}$	$0.466 \pm 0.201_{(3)}$	$0.495 \pm 0.009_{(3)}$
RadDINO	$0.441 \pm 0.057_{(3)}$	$0.716 \pm 0.023_{(3)}$	$0.512 \pm 0.388_{(3)}$	$0.498 \pm 0.015_{(3)}$
ResNet-50 (ImageNet)	$0.477 \pm 0.003_{(3)}$	$0.675 \pm 0.053_{(3)}$	$0.614 \pm 0.119_{(3)}$	$0.492 \pm 0.014_{(3)}$
ResNet-50 (Random)	$0.459 \pm 0.019_{(3)}$	$0.689 \pm 0.012_{(3)}$	$0.489 \pm 0.213_{(3)}$	$0.499 \pm 0.017_{(3)}$
RET-CLIP	$0.296 \pm 0.002_{(3)}$	$0.200 \pm 0.094_{(3)}$	$0.359 \pm 0.455_{(3)}$	$0.495 \pm 0.011_{(3)}$
RETFound DINOv2 (MEH)	$0.488 \pm 0.011_{(3)}$	$0.574 \pm 0.035_{(3)}$	$0.529 \pm 0.019_{(3)}$	$0.510 \pm 0.012_{(3)}$
RETFound DINOv2 (Shanghai)	$0.454 \pm 0.006_{(3)}$	$0.695 \pm 0.090_{(3)}$	$0.525 \pm 0.192_{(3)}$	$0.479 \pm 0.012_{(3)}$
RETFound MAE (MEH)	$0.516 \pm 0.029_{(3)}$	$0.674 \pm 0.027_{(3)}$	$0.562 \pm 0.319_{(3)}$	$0.491 \pm 0.024_{(3)}$
RETFound MAE (Nature CFP)	$0.492 \pm 0.013_{(3)}$	$0.654 \pm 0.084_{(3)}$	$0.403 \pm 0.371_{(3)}$	$0.485 \pm 0.002_{(3)}$
RETFound MAE (Nature OCT)	$0.438 \pm 0.022_{(3)}$	$0.592 \pm 0.009_{(3)}$	$0.398 \pm 0.123_{(3)}$	$0.498 \pm 0.015_{(3)}$
RETFound MAE (Shanghai)	$0.498 \pm 0.031_{(3)}$	$0.547 \pm 0.026_{(3)}$	$0.502 \pm 0.409_{(3)}$	$0.506 \pm 0.017_{(3)}$
RETFound Green	$0.449 \pm 0.012_{(3)}$	$0.663 \pm 0.063_{(3)}$	$0.464 \pm 0.170_{(3)}$	$0.480 \pm 0.007_{(3)}$
RETIZero	$0.507 \pm 0.040_{(3)}$	$0.624 \pm 0.021_{(3)}$	$0.354 \pm 0.258_{(3)}$	$0.490 \pm 0.003_{(3)}$
SigLIP	$0.533 \pm 0.017_{(3)}$	$0.695 \pm 0.024_{(3)}$	$0.571 \pm 0.178_{(3)}$	$0.495 \pm 0.015_{(3)}$
SigLIP2	$0.473 \pm 0.013_{(3)}$	$0.622 \pm 0.023_{(3)}$	$0.657 \pm 0.334_{(3)}$	$0.498 \pm 0.008_{(3)}$
URFound	$0.533 \pm 0.023_{(3)}$	$0.730 \pm 0.032_{(3)}$	$0.449 \pm 0.338_{(3)}$	$0.500 \pm 0.011_{(3)}$
ViLRef	$0.296 \pm 0.002_{(3)}$	$0.200 \pm 0.094_{(3)}$	$0.359 \pm 0.455_{(3)}$	$0.495 \pm 0.011_{(3)}$
Virchow	$0.493 \pm 0.056_{(3)}$	$0.712 \pm 0.065_{(3)}$	$0.727 \pm 0.214_{(3)}$	$0.491 \pm 0.005_{(3)}$
Virchow2	$0.484 \pm 0.008_{(3)}$	$0.683 \pm 0.053_{(3)}$	$0.522 \pm 0.167_{(3)}$	$0.490 \pm 0.010_{(3)}$
VisionFM Fundus	$0.440 \pm 0.030_{(3)}$	$0.592 \pm 0.066_{(3)}$	$0.550 \pm 0.211_{(3)}$	$0.501 \pm 0.014_{(3)}$
VisionFM OCT	$0.486 \pm 0.023_{(3)}$	$0.684 \pm 0.019_{(3)}$	$0.381 \pm 0.090_{(3)}$	$0.488 \pm 0.001_{(3)}$
ViT-B/16 CLIP	$0.487 \pm 0.020_{(3)}$	$0.664 \pm 0.067_{(3)}$	$0.571 \pm 0.134_{(3)}$	$0.493 \pm 0.005_{(3)}$
ViT-B/32 CLIP	$0.475 \pm 0.015_{(3)}$	$0.666 \pm 0.021_{(3)}$	$0.484 \pm 0.063_{(3)}$	$0.495 \pm 0.006_{(3)}$
ViT-L/14 CLIP	$0.502 \pm 0.025_{(3)}$	$0.636 \pm 0.059_{(3)}$	$0.531 \pm 0.139_{(3)}$	$0.502 \pm 0.018_{(3)}$
ViT-B (ImageNet)	$0.489 \pm 0.029_{(3)}$	$0.668 \pm 0.052_{(3)}$	$0.534 \pm 0.263_{(3)}$	$0.494 \pm 0.013_{(3)}$

Table A.16: Performance comparison across datasets using full training. Values show test accuracy as mean ± standard deviation. – indicates no data available. Subscript numbers in parentheses indicate sample size.

Model	Harvard FairVision Multiclass	Harvard GF Glaucoma	Harvard GF MD	HRF
BE-DINORET	$0.349 \pm 0.011_{(3)}$	$0.682 \pm 0.010_{(3)}$	$0.434 \pm 0.016_{(3)}$	$0.524 \pm 0.218_{(3)}$
DINORET	$0.373 \pm 0.017_{(3)}$	$0.712 \pm 0.017_{(3)}$	$0.467 \pm 0.036_{(3)}$	$0.524 \pm 0.082_{(3)}$
DINOv2 ViT-B/14	$0.339 \pm 0.008_{(3)}$	$0.683 \pm 0.008_{(3)}$	$0.425 \pm 0.008_{(3)}$	$0.667 \pm 0.082_{(3)}$
DINOv2 ViT-B/14 REG	$0.349 \pm 0.040_{(3)}$	$0.700 \pm 0.023_{(3)}$	$0.424 \pm 0.017_{(3)}$	$0.619 \pm 0.082_{(3)}$
DINOv2 ViT-G/14	$0.326 \pm 0.032_{(3)}$	$0.685 \pm 0.005_{(3)}$	$0.316 \pm 0.104_{(3)}$	$0.571 \pm 0.143_{(3)}$
DINOv2 ViT-G/14 REG	$0.343 \pm 0.028_{(3)}$	$0.692 \pm 0.018_{(3)}$	$0.441 \pm 0.021_{(3)}$	$0.619 \pm 0.218_{(3)}$
DINOv2 ViT-S/14	$0.383 \pm 0.016_{(3)}$	$0.700 \pm 0.010_{(3)}$	$0.386 \pm 0.067_{(3)}$	$0.619 \pm 0.082_{(3)}$
DINOv2 ViT-S/14 REG	$0.373 \pm 0.045_{(3)}$	$0.702 \pm 0.011_{(3)}$	$0.453 \pm 0.030_{(3)}$	$0.524 \pm 0.082_{(3)}$
LoRA-DINORET	$0.344 \pm 0.057_{(3)}$	$0.710 \pm 0.025_{(3)}$	$0.442 \pm 0.022_{(3)}$	$0.714 \pm 0.143_{(3)}$
Prov-GigaPath	$0.347 \pm 0.005_{(3)}$	$0.691 \pm 0.010_{(3)}$	$0.413 \pm 0.013_{(3)}$	$0.714_{(3)}$
RadDINO	$0.347 \pm 0.065_{(3)}$	$0.704 \pm 0.002_{(3)}$	$0.415 \pm 0.013_{(3)}$	$0.571_{(3)}$
ResNet-50 (ImageNet)	$0.361 \pm 0.006_{(3)}$	$0.706 \pm 0.030_{(3)}$	$0.430 \pm 0.018_{(3)}$	$0.524 \pm 0.082_{(3)}$
ResNet-50 (Random)	$0.334 \pm 0.034_{(3)}$	$0.710 \pm 0.011_{(3)}$	$0.430 \pm 0.039_{(3)}$	$0.667 \pm 0.165_{(3)}$
RET-CLIP	$0.305 \pm 0.124_{(3)}$	$0.597 \pm 0.025_{(3)}$	$0.290 \pm 0.019_{(3)}$	$0.286 \pm 0.143_{(3)}$
RETFound DINOv2 (MEH)	$0.323 \pm 0.035_{(3)}$	$0.640 \pm 0.018_{(3)}$	$0.372 \pm 0.047_{(3)}$	$0.762 \pm 0.082_{(3)}$
RETFound DINOv2 (Shanghai)	$0.352 \pm 0.034_{(3)}$	$0.706 \pm 0.013_{(3)}$	$0.440 \pm 0.022_{(3)}$	$0.667 \pm 0.082_{(3)}$
RETFound MAE (MEH)	$0.365 \pm 0.009_{(3)}$	$0.714 \pm 0.015_{(3)}$	$0.456 \pm 0.011_{(3)}$	$0.762 \pm 0.082_{(3)}$
RETFound MAE (Nature CFP)	$0.366 \pm 0.009_{(3)}$	$0.710 \pm 0.013_{(3)}$	$0.466 \pm 0.012_{(3)}$	$0.667 \pm 0.082_{(3)}$
RETFound MAE (Nature OCT)	$0.322 \pm 0.039_{(3)}$	$0.715 \pm 0.021_{(3)}$	$0.439 \pm 0.020_{(3)}$	$0.429 \pm 0.143_{(3)}$
RETFound MAE (Shanghai)	$0.378 \pm 0.004_{(3)}$	$0.704 \pm 0.003_{(3)}$	$0.445 \pm 0.020_{(3)}$	$0.619 \pm 0.082_{(3)}$
RETFound Green	$0.359 \pm 0.028_{(3)}$	$0.692 \pm 0.017_{(3)}$	$0.444 \pm 0.006_{(3)}$	$0.476 \pm 0.082_{(3)}$
RETIZero	$0.361 \pm 0.007_{(3)}$	$0.706 \pm 0.007_{(3)}$	$0.441 \pm 0.018_{(3)}$	$0.714 \pm 0.143_{(3)}$
SigLIP	$0.360 \pm 0.046_{(3)}$	$0.714 \pm 0.008_{(3)}$	$0.440 \pm 0.044_{(3)}$	$0.667 \pm 0.082_{(3)}$
SigLIP2	$0.346 \pm 0.021_{(3)}$	$0.657 \pm 0.016_{(3)}$	$0.392 \pm 0.034_{(3)}$	$0.905 \pm 0.082_{(3)}$
URFound	$0.383 \pm 0.005_{(3)}$	$0.731 \pm 0.002_{(3)}$	$0.499 \pm 0.009_{(3)}$	$0.714_{(3)}$
ViLRef	$0.305 \pm 0.124_{(3)}$	$0.597 \pm 0.025_{(3)}$	$0.290 \pm 0.019_{(3)}$	$0.286 \pm 0.143_{(3)}$
Virchow	$0.373 \pm 0.009_{(3)}$	$0.702 \pm 0.010_{(3)}$	$0.478 \pm 0.020_{(3)}$	$0.571 \pm 0.143_{(3)}$
Virchow2	$0.361 \pm 0.041_{(3)}$	$0.718 \pm 0.008_{(3)}$	$0.464 \pm 0.028_{(3)}$	$0.714 \pm 0.143_{(3)}$
VisionFM Fundus	$0.325 \pm 0.019_{(3)}$	$0.674 \pm 0.015_{(3)}$	$0.387 \pm 0.037_{(3)}$	$0.571_{(3)}$
VisionFM OCT	$0.321 \pm 0.026_{(3)}$	$0.736 \pm 0.002_{(3)}$	$0.458 \pm 0.058_{(3)}$	$0.571 \pm 0.143_{(3)}$
ViT-B/16 CLIP	$0.323 \pm 0.016_{(3)}$	$0.702 \pm 0.025_{(3)}$	$0.413 \pm 0.063_{(3)}$	$0.619 \pm 0.165_{(3)}$
ViT-B/32 CLIP	$0.340 \pm 0.038_{(3)}$	$0.690 \pm 0.008_{(3)}$	$0.431 \pm 0.033_{(3)}$	$0.619 \pm 0.165_{(3)}$
ViT-L/14 CLIP	$0.352 \pm 0.035_{(3)}$	$0.712 \pm 0.028_{(3)}$	$0.410 \pm 0.022_{(3)}$	$0.571 \pm 0.143_{(3)}$
ViT-B (ImageNet)	$0.385 \pm 0.007_{(3)}$	$0.718 \pm 0.019_{(3)}$	$0.439 \pm 0.031_{(3)}$	$0.714 \pm 0.143_{(3)}$

Table A.17: Performance comparison across datasets using full training. Values show test accuracy as mean ± standard deviation. – indicates no data available. Subscript numbers in parentheses indicate sample size.

Model	IDRiD	IDRiD ME	JustRAIGS	MBRSET DR
BE-DINORET	$0.482 \pm 0.077_{(3)}$	$0.699 \pm 0.035_{(3)}$	$0.856 \pm 0.010_{(3)}$	$0.437 \pm 0.030_{(3)}$
DINORET	$0.473 \pm 0.076_{(3)}$	$0.691 \pm 0.026_{(3)}$	$0.883 \pm 0.019_{(3)}$	$0.391 \pm 0.147_{(3)}$
DINOv2 ViT-B/14	$0.507 \pm 0.061_{(3)}$	$0.700 \pm 0.019_{(3)}$	$0.857 \pm 0.005_{(3)}$	$0.418 \pm 0.023_{(3)}$
DINOv2 ViT-B/14 REG	$0.494 \pm 0.062_{(3)}$	$0.691 \pm 0.057_{(3)}$	$0.896 \pm 0.006_{(3)}$	$0.411 \pm 0.089_{(3)}$
DINOv2 ViT-G/14	$0.460 \pm 0.067_{(3)}$	$0.692 \pm 0.044_{(3)}$	$0.897 \pm 0.006_{(3)}$	$0.318 \pm 0.138_{(3)}$
DINOv2 ViT-G/14 REG	$0.447 \pm 0.004_{(3)}$	$0.722 \pm 0.039_{(3)}$	$0.882 \pm 0.006_{(3)}$	$0.352 \pm 0.102_{(3)}$
DINOv2 ViT-S/14	$0.443 \pm 0.073_{(3)}$	$0.699 \pm 0.072_{(3)}$	$0.904 \pm 0.011_{(3)}$	$0.407 \pm 0.069_{(3)}$
DINOv2 ViT-S/14 REG	$0.498 \pm 0.047_{(3)}$	$0.718 \pm 0.059_{(3)}$	$0.906 \pm 0.019_{(3)}$	$0.429 \pm 0.195_{(3)}$
LoRA-DINORET	$0.477 \pm 0.046_{(3)}$	$0.740 \pm 0.027_{(3)}$	$0.920 \pm 0.008_{(3)}$	$0.444 \pm 0.022_{(3)}$
Prov-GigaPath	$0.532 \pm 0.083_{(3)}$	$0.752 \pm 0.041_{(3)}$	$0.816 \pm 0.007_{(3)}$	$0.512 \pm 0.053_{(3)}$
RadDINO	$0.426 \pm 0.026_{(3)}$	$0.691 \pm 0.033_{(3)}$	$0.883 \pm 0.006_{(3)}$	$0.437 \pm 0.113_{(3)}$
ResNet-50 (ImageNet)	$0.410 \pm 0.063_{(3)}$	$0.743 \pm 0.057_{(3)}$	$0.880 \pm 0.008_{(3)}$	$0.455 \pm 0.056_{(3)}$
ResNet-50 (Random)	$0.219 \pm 0.048_{(3)}$	$0.504 \pm 0.064_{(3)}$	$0.816 \pm 0.013_{(3)}$	$0.152 \pm 0.146_{(3)}$
RET-CLIP	$0.261 \pm 0.064_{(3)}$	$0.449 \pm 0.068_{(3)}$	$0.611 \pm 0.035_{(3)}$	$0.200 \pm 0.091_{(3)}$
RETFound DINOv2 (MEH)	$0.439 \pm 0.059_{(3)}$	$0.696 \pm 0.032_{(3)}$	$0.902 \pm 0.003_{(3)}$	$0.468 \pm 0.038_{(3)}$
RETFound DINOv2 (Shanghai)	$0.435 \pm 0.030_{(3)}$	$0.722 \pm 0.015_{(3)}$	$0.922 \pm 0.002_{(3)}$	$0.424 \pm 0.028_{(3)}$
RETFound MAE (MEH)	$0.444 \pm 0.125_{(3)}$	$0.696 \pm 0.059_{(3)}$	$0.882 \pm 0.005_{(3)}$	$0.545 \pm 0.068_{(3)}$
RETFound MAE (Nature CFP)	$0.494 \pm 0.061_{(3)}$	$0.661 \pm 0.052_{(3)}$	$0.889 \pm 0.006_{(3)}$	$0.543 \pm 0.161_{(3)}$
RETFound MAE (Nature OCT)	$0.261 \pm 0.024_{(3)}$	$0.531 \pm 0.086_{(3)}$	$0.668 \pm 0.014_{(3)}$	$0.297 \pm 0.033_{(3)}$
RETFound MAE (Shanghai)	$0.481 \pm 0.067_{(3)}$	$0.705 \pm 0.034_{(3)}$	$0.902 \pm 0.005_{(3)}$	$0.446 \pm 0.051_{(3)}$
RETFound Green	$0.477 \pm 0.097_{(3)}$	$0.717 \pm 0.068_{(3)}$	$0.880 \pm 0.006_{(3)}$	$0.379 \pm 0.066_{(3)}$
RETIZero	$0.540 \pm 0.081_{(3)}$	$0.761 \pm 0.034_{(3)}$	$0.904 \pm 0.012_{(3)}$	$0.574 \pm 0.124_{(3)}$
SigLIP	$0.472 \pm 0.013_{(3)}$	$0.709 \pm 0.032_{(3)}$	$0.895 \pm 0.013_{(3)}$	$0.437 \pm 0.278_{(3)}$
SigLIP2	$0.460 \pm 0.105_{(3)}$	$0.688 \pm 0.059_{(3)}$	$0.842 \pm 0.005_{(3)}$	$0.442 \pm 0.115_{(3)}$
URFound	$0.498 \pm 0.157_{(3)}$	$0.766 \pm 0.028_{(3)}$	$0.900 \pm 0.006_{(3)}$	$0.504 \pm 0.102_{(3)}$
ViLRef	$0.261 \pm 0.064_{(3)}$	$0.449 \pm 0.068_{(3)}$	$0.611 \pm 0.035_{(3)}$	$0.200 \pm 0.091_{(3)}$
Virchow	$0.473 \pm 0.079_{(3)}$	$0.709 \pm 0.026_{(3)}$	$0.915 \pm 0.011_{(3)}$	$0.494 \pm 0.242_{(3)}$
Virchow2	$0.422 \pm 0.042_{(3)}$	$0.707 \pm 0.068_{(3)}$	$0.881 \pm 0.012_{(3)}$	$0.549 \pm 0.085_{(3)}$
VisionFM Fundus	$0.384 \pm 0.049_{(3)}$	$0.626 \pm 0.060_{(3)}$	$0.890 \pm 0.005_{(3)}$	$0.397 \pm 0.051_{(3)}$
VisionFM OCT	$0.384 \pm 0.045_{(3)}$	$0.631 \pm 0.043_{(3)}$	$0.867 \pm 0.024_{(3)}$	$0.197 \pm 0.118_{(3)}$
ViT-B/16 CLIP	$0.481 \pm 0.039_{(3)}$	$0.722 \pm 0.071_{(3)}$	$0.888 \pm 0.002_{(3)}$	$0.494 \pm 0.069_{(3)}$
ViT-B/32 CLIP	$0.447 \pm 0.016_{(3)}$	$0.661 \pm 0.019_{(3)}$	$0.869 \pm 0.006_{(3)}$	$0.432 \pm 0.091_{(3)}$
ViT-L/14 CLIP	$0.431 \pm 0.114_{(3)}$	$0.730 \pm 0.038_{(3)}$	$0.906 \pm 0.012_{(3)}$	$0.389 \pm 0.175_{(3)}$
ViT-B (ImageNet)	$0.490 \pm 0.013_{(3)}$	$0.687 \pm 0.031_{(3)}$	$0.877 \pm 0.024_{(3)}$	$0.399 \pm 0.025_{(3)}$

Table A.18: Performance comparison across datasets using full training. Values show test accuracy as mean ± standard deviation. – indicates no data available. Subscript numbers in parentheses indicate sample size.

Model	MBRSET HT	MBRSET Microvascular	MBRSET Obesity	MESSIDOR
BE-DINORET	$0.506 \pm 0.040_{(3)}$	$0.575 \pm 0.008_{(3)}$	$0.551 \pm 0.079_{(3)}$	$0.427 \pm 0.059_{(3)}$
DINORET	$0.552 \pm 0.116_{(3)}$	$0.538 \pm 0.100_{(3)}$	$0.660 \pm 0.134_{(3)}$	$0.434 \pm 0.113_{(3)}$
DINOv2 ViT-B/14	$0.594 \pm 0.032_{(3)}$	$0.602 \pm 0.007_{(3)}$	$0.601 \pm 0.111_{(3)}$	$0.435 \pm 0.060_{(3)}$
DINOv2 ViT-B/14 REG	$0.550 \pm 0.012_{(3)}$	$0.538 \pm 0.052_{(3)}$	$0.491 \pm 0.158_{(3)}$	$0.344 \pm 0.030_{(3)}$
DINOv2 ViT-G/14	$0.411 \pm 0.120_{(3)}$	$0.592 \pm 0.054_{(3)}$	$0.210 \pm 0.082_{(3)}$	$0.338 \pm 0.088_{(3)}$
DINOv2 ViT-G/14 REG	$0.569 \pm 0.067_{(3)}$	$0.511 \pm 0.082_{(3)}$	$0.444 \pm 0.064_{(3)}$	$0.427 \pm 0.051_{(3)}$
DINOv2 ViT-S/14	$0.605 \pm 0.051_{(3)}$	$0.532 \pm 0.038_{(3)}$	$0.611 \pm 0.254_{(3)}$	$0.434 \pm 0.097_{(3)}$
DINOv2 ViT-S/14 REG	$0.530 \pm 0.030_{(3)}$	$0.553 \pm 0.076_{(3)}$	$0.460 \pm 0.139_{(3)}$	$0.365 \pm 0.023_{(3)}$
LoRA-DINORET	$0.497 \pm 0.058_{(3)}$	$0.527 \pm 0.021_{(3)}$	$0.445 \pm 0.115_{(3)}$	$0.419 \pm 0.066_{(3)}$
Prov-GigaPath	$0.531 \pm 0.025_{(3)}$	$0.556 \pm 0.026_{(3)}$	$0.576 \pm 0.185_{(3)}$	$0.444 \pm 0.043_{(3)}$
RadDINO	$0.532 \pm 0.030_{(3)}$	$0.500 \pm 0.027_{(3)}$	$0.558 \pm 0.082_{(3)}$	$0.326 \pm 0.099_{(3)}$
ResNet-50 (ImageNet)	$0.603 \pm 0.018_{(3)}$	$0.532 \pm 0.018_{(3)}$	$0.482 \pm 0.215_{(3)}$	$0.424 \pm 0.040_{(3)}$
ResNet-50 (Random)	$0.502 \pm 0.120_{(3)}$	$0.426 \pm 0.042_{(3)}$	$0.616 \pm 0.155_{(3)}$	$0.290 \pm 0.088_{(3)}$
RET-CLIP	$0.533 \pm 0.054_{(3)}$	$0.519 \pm 0.077_{(3)}$	$0.380 \pm 0.317_{(3)}$	$0.204 \pm 0.169_{(3)}$
RETFound DINOv2 (MEH)	$0.552 \pm 0.034_{(3)}$	$0.507 \pm 0.024_{(3)}$	$0.462 \pm 0.012_{(3)}$	$0.435 \pm 0.077_{(3)}$
RETFound DINOv2 (Shanghai)	$0.563 \pm 0.018_{(3)}$	$0.514 \pm 0.015_{(3)}$	$0.450 \pm 0.043_{(3)}$	$0.405 \pm 0.067_{(3)}$
RETFound MAE (MEH)	$0.596 \pm 0.034_{(3)}$	$0.554 \pm 0.026_{(3)}$	$0.705 \pm 0.189_{(3)}$	$0.422 \pm 0.078_{(3)}$
RETFound MAE (Nature CFP)	$0.549 \pm 0.092_{(3)}$	$0.503 \pm 0.072_{(3)}$	$0.329 \pm 0.398_{(3)}$	$0.439 \pm 0.103_{(3)}$
RETFound MAE (Nature OCT)	$0.504 \pm 0.033_{(3)}$	$0.497 \pm 0.061_{(3)}$	$0.620 \pm 0.215_{(3)}$	$0.248 \pm 0.054_{(3)}$
RETFound MAE (Shanghai)	$0.620 \pm 0.046_{(3)}$	$0.540 \pm 0.042_{(3)}$	$0.545 \pm 0.134_{(3)}$	$0.486 \pm 0.081_{(3)}$
RETFound Green	$0.543 \pm 0.081_{(3)}$	$0.565 \pm 0.019_{(3)}$	$0.350 \pm 0.173_{(3)}$	$0.449 \pm 0.075_{(3)}$
RETIZero	$0.598 \pm 0.069_{(3)}$	$0.595 \pm 0.027_{(3)}$	$0.612 \pm 0.203_{(3)}$	$0.458 \pm 0.071_{(3)}$
SigLIP	$0.580 \pm 0.171_{(3)}$	$0.530 \pm 0.045_{(3)}$	$0.562 \pm 0.357_{(3)}$	$0.500 \pm 0.080_{(3)}$
SigLIP2	$0.511 \pm 0.031_{(3)}$	$0.554 \pm 0.059_{(3)}$	$0.686 \pm 0.231_{(3)}$	$0.431 \pm 0.099_{(3)}$
URFound	$0.556 \pm 0.043_{(3)}$	$0.522 \pm 0.078_{(3)}$	$0.489 \pm 0.276_{(3)}$	$0.475 \pm 0.057_{(3)}$
ViLRef	$0.533 \pm 0.054_{(3)}$	$0.519 \pm 0.077_{(3)}$	$0.380 \pm 0.317_{(3)}$	$0.204 \pm 0.169_{(3)}$
Virchow	$0.446 \pm 0.021_{(3)}$	$0.509 \pm 0.038_{(3)}$	$0.540 \pm 0.193_{(3)}$	$0.416 \pm 0.099_{(3)}$
Virchow2	$0.479 \pm 0.102_{(3)}$	$0.557 \pm 0.031_{(3)}$	$0.540 \pm 0.191_{(3)}$	$0.453 \pm 0.086_{(3)}$
VisionFM Fundus	$0.517 \pm 0.023_{(3)}$	$0.527 \pm 0.046_{(3)}$	$0.445 \pm 0.044_{(3)}$	$0.338 \pm 0.015_{(3)}$
VisionFM OCT	$0.557 \pm 0.040_{(3)}$	$0.449 \pm 0.096_{(3)}$	$0.405 \pm 0.234_{(3)}$	$0.253 \pm 0.053_{(3)}$
ViT-B/16 CLIP	$0.542 \pm 0.080_{(3)}$	$0.554 \pm 0.047_{(3)}$	$0.451 \pm 0.046_{(3)}$	$0.338 \pm 0.032_{(3)}$
ViT-B/32 CLIP	$0.533 \pm 0.024_{(3)}$	$0.558 \pm 0.035_{(3)}$	$0.405 \pm 0.003_{(3)}$	$0.377 \pm 0.056_{(3)}$
ViT-L/14 CLIP	$0.628 \pm 0.067_{(3)}$	$0.553 \pm 0.047_{(3)}$	$0.630 \pm 0.194_{(3)}$	$0.318 \pm 0.055_{(3)}$
ViT-B (ImageNet)	$0.579 \pm 0.053_{(3)}$	$0.542 \pm 0.062_{(3)}$	$0.449 \pm 0.091_{(3)}$	$0.364 \pm 0.061_{(3)}$

Table A.19: Performance comparison across datasets using full training. Values show test accuracy as mean ± standard deviation. – indicates no data available. Subscript numbers in parentheses indicate sample size.

Model	MESSIDOR DME	OCT X-ray	OCTID	OUWFFI
BE-DINORET	$0.852 \pm 0.048_{(3)}$	$0.886 \pm 0.021_{(3)}$	$0.903 \pm 0.036_{(3)}$	$0.758 \pm 0.005_{(3)}$
DINORET	$0.879 \pm 0.041_{(3)}$	$0.948 \pm 0.015_{(3)}$	$0.903 \pm 0.036_{(3)}$	$0.755 \pm 0.053_{(3)}$
DINOv2 ViT-B/14	$0.864 \pm 0.037_{(3)}$	$0.898 \pm 0.009_{(3)}$	$0.911 \pm 0.024_{(3)}$	$0.774 \pm 0.025_{(3)}$
DINOv2 ViT-B/14 REG	$0.891 \pm 0.018_{(3)}$	$0.936 \pm 0.015_{(3)}$	$0.915 \pm 0.018_{(3)}$	$0.777 \pm 0.052_{(3)}$
DINOv2 ViT-G/14	$0.864 \pm 0.025_{(3)}$	$0.947 \pm 0.017_{(3)}$	$0.922 \pm 0.029_{(3)}$	$0.736 \pm 0.047_{(3)}$
DINOv2 ViT-G/14 REG	$0.846 \pm 0.040_{(3)}$	$0.911 \pm 0.012_{(3)}$	$0.907 \pm 0.042_{(3)}$	$0.774 \pm 0.025_{(3)}$
DINOv2 ViT-S/14	$0.875 \pm 0.056_{(3)}$	$0.945 \pm 0.017_{(3)}$	$0.953 \pm 0.020_{(3)}$	$0.780 \pm 0.022_{(3)}$
DINOv2 ViT-S/14 REG	$0.870 \pm 0.031_{(3)}$	$0.941 \pm 0.018_{(3)}$	$0.938 \pm 0.013_{(3)}$	$0.808 \pm 0.022_{(3)}$
LoRA-DINORET	$0.838 \pm 0.021_{(3)}$	$0.947 \pm 0.016_{(3)}$	$0.946 \pm 0.024_{(3)}$	$0.764 \pm 0.009_{(3)}$
Prov-GigaPath	$0.856 \pm 0.006_{(3)}$	$0.858 \pm 0.021_{(3)}$	$0.911 \pm 0.018_{(3)}$	$0.789 \pm 0.005_{(3)}$
RadDINO	$0.835 \pm 0.020_{(3)}$	$0.926 \pm 0.017_{(3)}$	$0.888 \pm 0.029_{(3)}$	$0.704 \pm 0.047_{(3)}$
ResNet-50 (ImageNet)	$0.873 \pm 0.018_{(3)}$	$0.922 \pm 0.022_{(3)}$	$0.926 \pm 0.036_{(3)}$	$0.761 \pm 0.014_{(3)}$
ResNet-50 (Random)	$0.659 \pm 0.143_{(3)}$	$0.907 \pm 0.016_{(3)}$	$0.810 \pm 0.047_{(3)}$	$0.632 \pm 0.038_{(3)}$
RET-CLIP	$0.599 \pm 0.052_{(3)}$	$0.406 \pm 0.057_{(3)}$	$0.500 \pm 0.012_{(3)}$	$0.327 \pm 0.038_{(3)}$
RETFound DINOv2 (MEH)	$0.860 \pm 0.024_{(3)}$	$0.802 \pm 0.035_{(3)}$	$0.860 \pm 0.012_{(3)}$	$0.755 \pm 0.019_{(3)}$
RETFound DINOv2 (Shanghai)	$0.887 \pm 0.010_{(3)}$	$0.936 \pm 0.021_{(3)}$	$0.946 \pm 0.007_{(3)}$	$0.808 \pm 0.043_{(3)}$
RETFound MAE (MEH)	$0.810 \pm 0.091_{(3)}$	$0.926 \pm 0.017_{(3)}$	$0.907 \pm 0.035_{(3)}$	$0.701 \pm 0.044_{(3)}$
RETFound MAE (Nature CFP)	$0.844 \pm 0.056_{(3)}$	$0.930 \pm 0.019_{(3)}$	$0.922 \pm 0.013_{(3)}$	$0.726 \pm 0.034_{(3)}$
RETFound MAE (Nature OCT)	$0.508 \pm 0.040_{(3)}$	$0.799 \pm 0.029_{(3)}$	$0.891 \pm 0.037_{(3)}$	$0.506 \pm 0.020_{(3)}$
RETFound MAE (Shanghai)	$0.837 \pm 0.042_{(3)}$	$0.932 \pm 0.020_{(3)}$	$0.915 \pm 0.029_{(3)}$	$0.770 \pm 0.014_{(3)}$
RETFound Green	$0.823 \pm 0.014_{(3)}$	$0.924 \pm 0.014_{(3)}$	$0.903 \pm 0.048_{(3)}$	$0.748 \pm 0.014_{(3)}$
RETIZero	$0.841 \pm 0.066_{(3)}$	$0.909 \pm 0.007_{(3)}$	$0.907 \pm 0.023_{(3)}$	$0.777 \pm 0.005_{(3)}$
SigLIP	$0.865 \pm 0.044_{(3)}$	$0.944 \pm 0.018_{(3)}$	$0.934 \pm 0.029_{(3)}$	$0.752 \pm 0.036_{(3)}$
SigLIP2	$0.861 \pm 0.033_{(3)}$	$0.890 \pm 0.015_{(3)}$	$0.903 \pm 0.071_{(3)}$	$0.742 \pm 0.024_{(3)}$
URFound	$0.880 \pm 0.021_{(3)}$	$0.942 \pm 0.023_{(3)}$	$0.919 \pm 0.020_{(3)}$	$0.748 \pm 0.052_{(3)}$
ViLRef	$0.599 \pm 0.052_{(3)}$	$0.406 \pm 0.057_{(3)}$	$0.500 \pm 0.012_{(3)}$	$0.327 \pm 0.038_{(3)}$
Virchow	$0.845 \pm 0.100_{(3)}$	$0.939 \pm 0.018_{(3)}$	$0.922 \pm 0.041_{(3)}$	$0.789 \pm 0.014_{(3)}$
Virchow2	$0.884 \pm 0.023_{(3)}$	$0.938 \pm 0.016_{(3)}$	$0.926 \pm 0.027_{(3)}$	$0.777 \pm 0.029_{(3)}$
VisionFM Fundus	$0.826 \pm 0.058_{(3)}$	$0.840 \pm 0.018_{(3)}$	$0.822 \pm 0.018_{(3)}$	$0.745 \pm 0.033_{(3)}$
VisionFM OCT	$0.630 \pm 0.126_{(3)}$	$0.938 \pm 0.015_{(3)}$	$0.942 \pm 0.020_{(3)}$	$0.591 \pm 0.086_{(3)}$
ViT-B/16 CLIP	$0.836 \pm 0.023_{(3)}$	$0.928 \pm 0.018_{(3)}$	$0.926 \pm 0.027_{(3)}$	$0.786 \pm 0.022_{(3)}$
ViT-B/32 CLIP	$0.796 \pm 0.028_{(3)}$	$0.921 \pm 0.013_{(3)}$	$0.915 \pm 0.047_{(3)}$	$0.758 \pm 0.020_{(3)}$
ViT-L/14 CLIP	$0.851 \pm 0.030_{(3)}$	$0.944 \pm 0.017_{(3)}$	$0.942 \pm 0.031_{(3)}$	$0.777 \pm 0.014_{(3)}$
ViT-B (ImageNet)	$0.793 \pm 0.015_{(3)}$	$0.932 \pm 0.027_{(3)}$	$0.942 \pm 0.031_{(3)}$	$0.758 \pm 0.024_{(3)}$

Table A.20: Performance comparison across datasets using full training. Values show test accuracy as mean ± standard deviation. – indicates no data available. Subscript numbers in parentheses indicate sample size.

Model	PAPILA	RFMiD Binary	RFMiD Multi
BE-DINORET	$0.491 \pm 0.064_{(3)}$	$0.852 \pm 0.013_{(3)}$	$0.487 \pm 0.047_{(3)}$
DINORET	$0.495 \pm 0.021_{(3)}$	$0.875 \pm 0.009_{(3)}$	$0.389 \pm 0.020_{(3)}$
DINOv2 ViT-B/14	$0.563 \pm 0.021_{(3)}$	$0.843 \pm 0.014_{(3)}$	$0.466 \pm 0.033_{(3)}$
DINOv2 ViT-B/14 REG	$0.495 \pm 0.077_{(3)}$	$0.855 \pm 0.018_{(3)}$	$0.452 \pm 0.014_{(3)}$
DINOv2 ViT-G/14	$0.428 \pm 0.195_{(3)}$	$0.867 \pm 0.013_{(3)}$	$0.506 \pm 0.043_{(3)}$
DINOv2 ViT-G/14 REG	$0.446 \pm 0.036_{(3)}$	$0.875 \pm 0.009_{(3)}$	$0.500 \pm 0.058_{(3)}$
DINOv2 ViT-S/14	$0.509 \pm 0.096_{(3)}$	$0.868 \pm 0.021_{(3)}$	$0.479 \pm 0.072_{(3)}$
DINOv2 ViT-S/14 REG	$0.532 \pm 0.055_{(3)}$	$0.857 \pm 0.021_{(3)}$	$0.451 \pm 0.071_{(3)}$
LoRA-DINORET	$0.586 \pm 0.067_{(3)}$	$0.854 \pm 0.014_{(3)}$	$0.533 \pm 0.019_{(3)}$
Prov-GigaPath	$0.486 \pm 0.075_{(3)}$	$0.858 \pm 0.006_{(3)}$	$0.513 \pm 0.064_{(3)}$
RadDINO	$0.541 \pm 0.106_{(3)}$	$0.830 \pm 0.001_{(3)}$	$0.361 \pm 0.033_{(3)}$
ResNet-50 (ImageNet)	$0.527 \pm 0.049_{(3)}$	$0.852 \pm 0.010_{(3)}$	$0.460 \pm 0.040_{(3)}$
ResNet-50 (Random)	$0.428 \pm 0.077_{(3)}$	$0.812 \pm 0.025_{(3)}$	$0.209 \pm 0.022_{(3)}$
RET-CLIP	$0.302 \pm 0.055_{(3)}$	$0.597 \pm 0.054_{(3)}$	$0.153 \pm 0.033_{(3)}$
RETFound DINOv2 (MEH)	$0.568 \pm 0.036_{(3)}$	$0.854 \pm 0.008_{(3)}$	$0.518 \pm 0.041_{(3)}$
RETFound DINOv2 (Shanghai)	$0.586 \pm 0.047_{(3)}$	$0.842 \pm 0.009_{(3)}$	$0.548 \pm 0.028_{(3)}$
RETFound MAE (MEH)	$0.428 \pm 0.068_{(3)}$	$0.850 \pm 0.010_{(3)}$	$0.468 \pm 0.033_{(3)}$
RETFound MAE (Nature CFP)	$0.374 \pm 0.034_{(3)}$	$0.857 \pm 0.022_{(3)}$	$0.414 \pm 0.092_{(3)}$
RETFound MAE (Nature OCT)	$0.324 \pm 0.014_{(3)}$	$0.755 \pm 0.029_{(3)}$	$0.177 \pm 0.042_{(3)}$
RETFound MAE (Shanghai)	$0.459 \pm 0.068_{(3)}$	$0.853 \pm 0.002_{(3)}$	$0.454 \pm 0.058_{(3)}$
RETFound Green	$0.541 \pm 0.106_{(3)}$	$0.871 \pm 0.007_{(3)}$	$0.464 \pm 0.065_{(3)}$
RETIZero	$0.581 \pm 0.118_{(3)}$	$0.859 \pm 0.023_{(3)}$	$0.577 \pm 0.044_{(3)}$
SigLIP	$0.617 \pm 0.096_{(3)}$	$0.852 \pm 0.007_{(3)}$	$0.459 \pm 0.045_{(3)}$
SigLIP2	$0.541 \pm 0.089_{(3)}$	$0.853 \pm 0.002_{(3)}$	$0.462 \pm 0.054_{(3)}$
URFound	$0.464 \pm 0.055_{(3)}$	$0.879 \pm 0.015_{(3)}$	$0.563 \pm 0.103_{(3)}$
ViLRef	$0.302 \pm 0.055_{(3)}$	$0.597 \pm 0.054_{(3)}$	$0.153 \pm 0.033_{(3)}$
Virchow	$0.505 \pm 0.008_{(3)}$	$0.852 \pm 0.019_{(3)}$	$0.475 \pm 0.014_{(3)}$
Virchow2	$0.509 \pm 0.074_{(3)}$	$0.854 \pm 0.013_{(3)}$	$0.464 \pm 0.067_{(3)}$
VisionFM Fundus	$0.532 \pm 0.051_{(3)}$	$0.845 \pm 0.021_{(3)}$	$0.492 \pm 0.041_{(3)}$
VisionFM OCT	$0.392 \pm 0.082_{(3)}$	$0.816 \pm 0.020_{(3)}$	$0.291 \pm 0.003_{(3)}$
ViT-B/16 CLIP	$0.559 \pm 0.056_{(3)}$	$0.859 \pm 0.013_{(3)}$	$0.505 \pm 0.036_{(3)}$
ViT-B/32 CLIP	$0.532 \pm 0.028_{(3)}$	$0.861 \pm 0.009_{(3)}$	$0.463 \pm 0.045_{(3)}$
ViT-L/14 CLIP	$0.586 \pm 0.008_{(3)}$	$0.874 \pm 0.008_{(3)}$	$0.491 \pm 0.090_{(3)}$
ViT-B (ImageNet)	$0.577 \pm 0.021_{(3)}$	$0.850 \pm 0.015_{(3)}$	$0.513 \pm 0.030_{(3)}$

Table A.21: Performance comparison across datasets using full training. Values show test accuracy as mean ± standard deviation. – indicates no data available. Subscript numbers in parentheses indicate sample size.
